# *Pseudomonas aeruginosa* aggregation and Psl expression in sputum is associated with antibiotic eradication failure in children with cystic fibrosis

**DOI:** 10.1038/s41598-022-25889-6

**Published:** 2022-12-12

**Authors:** Amanda J. Morris, Yvonne C. W. Yau, Subin Park, Shafinaz Eisha, Nancy McDonald, Matthew R. Parsek, P. Lynne Howell, Lucas R. Hoffman, Dao Nguyen, Antonio DiGiandomenico, Ashley M. Rooney, Bryan Coburn, Lucia Grana-Miraglia, Pauline Wang, David S. Guttman, Daniel J. Wozniak, Valerie J. Waters

**Affiliations:** 1 Translational Medicine, Research Institute, Hospital for Sick Children, Toronto, ON Canada; 2 Division of Microbiology, Department of Pediatric Laboratory Medicine, Hospital for Sick Children, University of Toronto, Toronto, ON Canada; 3 Division of Respiratory Medicine, Hospital for Sick Children, Toronto, ON Canada; 4 Department of Microbiology, University of Washington, Seattle, WA USA; 5 Program in Molecular Medicine, Research Institute, Hospital for Sick Children, Toronto, ON Canada; 6 Department of Biochemistry, University of Toronto, Toronto, ON Canada; 7 Departments of Pediatrics and Microbiology, University of Washington, Seattle, WA USA; 8 Meakins-Christie Laboratories, Research Institute of the McGill University Health Centre, Montreal, QC Canada; 9 Department of Medicine, McGill University, Montreal, QC Canada; 10 Vaccines and Immune Therapies, Biopharmaceuticals R&D, AstraZeneca, Gaithersburg, MD USA; 11 Division of Infectious Diseases, Department of Medicine, University of Toronto, Toronto, ON Canada; 12 Department of Cell and Systems Biology, University of Toronto, Toronto, ON Canada; 13 Departments of Microbial Infection and Immunity, Microbiology, Ohio State University, Columbus, OH USA; 14 Division of Infectious Diseases, Department of Pediatrics, Hospital for Sick Children, 555 University Avenue, Toronto, ON M5G 1X8 Canada

**Keywords:** Microbial communities, Infectious-disease diagnostics

## Abstract

We previously demonstrated that *P. aeruginosa* isolates that persisted in children with cystic fibrosis (CF) despite inhaled tobramycin treatment had increased anti-Psl antibody binding in vitro compared to those successfully eradicated. We aimed to validate these findings by directly visualizing *P. aeruginosa* in CF sputum. This was a prospective observational study of children with CF with new-onset *P. aeruginosa* infection who underwent inhaled tobramycin eradication treatment. Using microbial identification passive clarity technique (MiPACT), *P. aeruginosa* was visualized in sputum samples obtained before treatment and classified as persistent or eradicated based on outcomes. Pre-treatment isolates were also grown as biofilms in vitro. Of 11 patients enrolled, 4 developed persistent infection and 7 eradicated infection. *P. aeruginosa* biovolume and the number as well as size of *P. aeruginosa* aggregates were greater in the sputum of those with persistent compared with eradicated infections (p < 0.01). The amount of Psl antibody binding in sputum was also greater overall (p < 0.05) in samples with increased *P. aeruginosa* biovolume. When visualized in sputum, *P. aeruginosa* had a greater biovolume, with more expressed Psl, and formed more numerous, larger aggregates in CF children who failed eradication therapy compared to those who successfully cleared their infection.

## Introduction

Individuals with cystic fibrosis (CF) are susceptible to pulmonary infection with *Pseudomonas aeruginosa* due to defects in the cystic fibrosis transmembrane conductance regulator (CFTR) that result in impaired mucociliary clearance^1^. Without antimicrobial treatment, *P. aeruginosa* can establish chronic infection in the airways which is associated with more rapid lung function decline and premature death in patients with CF^2,3^. Early antibiotic eradication treatment (AET) of initial *P. aeruginosa* colonization is thus standard clinical care of children with CF and increasing the success rate of AET is key in improving long-term outcomes^4,5^.

There are few explanations as to why AET may fail in a patient with CF. We previously showed, using a biofilm model visualized by confocal microscopy, that initial *P. aeruginosa* isolates from children who failed AET had significantly increased anti-Psl antibody binding compared to isolates from those who cleared *P. aeruginosa*^6^. Increased anti-Psl antibody binding was associated with bacterial aggregation and tobramycin tolerance. Psl is a *P. aeruginosa* exopolysaccharide (EPS) that has been well described for its role in cell–cell and cell-substrate attachment adhesion and biofilm formation in vitro^7–12^*.* Although our findings were confirmed in separate collections of early *P. aeruginosa* clinical isolates from two distinct AET trials, these results were based on in vitro studies of *P. aeruginosa* in a glass slide chamber system.

Despite the use of clinical isolates and bacterial growth conditions mimicking CF airways, lab-based models examining the behaviour of CF pathogens and the effects of antibiotics may not accurately reflect conditions in patient airways^13^. Decisions on incubation times, growth media and antimicrobial concentrations, for example, can significantly alter experimental outcomes and it is very challenging to fully capture the complexities of the CF lung environment. With these challenges, it is perhaps not surprising that there is a disconnect between antimicrobial susceptibility testing results and clinical outcomes in CF^14^.

Recent advancements in the processing and imaging of respiratory tract specimens, however, have allowed the direct visualization and interaction of microbial species in patient samples. The microbial identification passive clarity technique (MiPACT), developed by DePas et al., fixes and visualizes bacteria using hybridization chain reaction to detect rRNA in CF sputum^15^. These methodologies obviate the need to make inferences in translating in vitro to in vivo results. The goal of this study was to validate our previous findings of increased anti-Psl antibody binding and bacterial aggregation in persistent *P. aeruginosa* isolates by directly visualizing *P. aeruginosa* biofilms in sputum in a prospective, observational trial of children with CF undergoing AET. We also aimed to determine whether we could, in fact, replicate in situ results from clinical specimens using our in vitro biofilm slide chamber model.

## Results

### Patient characteristics

The characteristics of enrolled patients are presented in Table 1. A total of 11 CF patients with new onset *P. aeruginosa* infection produced a sputum sample before treatment initiation and were included in the study; 7 successfully eradicated *P. aeruginosa* after 1 month of inhaled tobramycin (no *P. aeruginosa* positive sputum culture for at least 6 months following the initial positive culture) whereas 4 developed persistent infection despite therapy (*P. aeruginosa* positive sputum culture in the 6 months following the initial positive culture) (see Methods). Of the 4 who developed persistent infection, 3 had follow up *P. aeruginosa* isolates available for whole genome sequencing demonstrating persistence of the same strain (less than 10 single nucleotide polymorphism difference between the initial and follow up strain). There were no significant differences between the two groups in terms of demographics such as age, sex, baseline lung function,nutritional status or proportion of patients on CFTR modulator therapy. Of note, there was no significant difference in the proportion of patients with an initial mucoid *P. aeruginosa* isolate who eradicated compared to developed persistent infection.

**Table 1:** Baseline patient characteristics.

	Eradicated (N = 7)	Persistent (N = 4)	p value
Age at incident infection, mean in years (SD)	12.7 (4.2)	15.9 (2.3)	0.23
Number of sputum samples/patient, median (range)^a^	4 (3–7)	6 (4–8)	0.12
Time from previous PA infection, mean in years (SD)	2.9 (3.1)	5.7 (2.5)	0.18
Mucoid PA infection, n (%)	3 (43%)	1 (25%)	0.57
Female, n (%)	4 (57%)	2 (50%)	0.81
DeltaF508 homozygous, n (%)	3 (43%)	1 (25%)	0.55
Pancreatic Insufficient, n (%)	7 (100%)	3 (75%)	0.36
Baseline FEV_1_% pred, median (range)	91 (77–120)	83 (67–103)	0.45
Baseline BMI percentile, median (range)	41 (8–94)	51 (23–86)	0.92
CFTR modulator therapy, n (%)	3 (43%)	1 (25%)	0.55

PA, *Pseudomonas aeruginosa*; *n*, number; %, percentage; FEV_1_% pred, predicted force expiratory volume in 1 s; BMI, body mass index; CFTR, cystic fibrosis transmembrane conductance regulator.

Baseline was defined as the FEV_1_% pred and BMI percentile value at the time of the new onset PA infection.

^a^In the 6 months following new onset PA infection.

### Pseudomonas aeruginosa visualization within sputum

**Figure 1:**
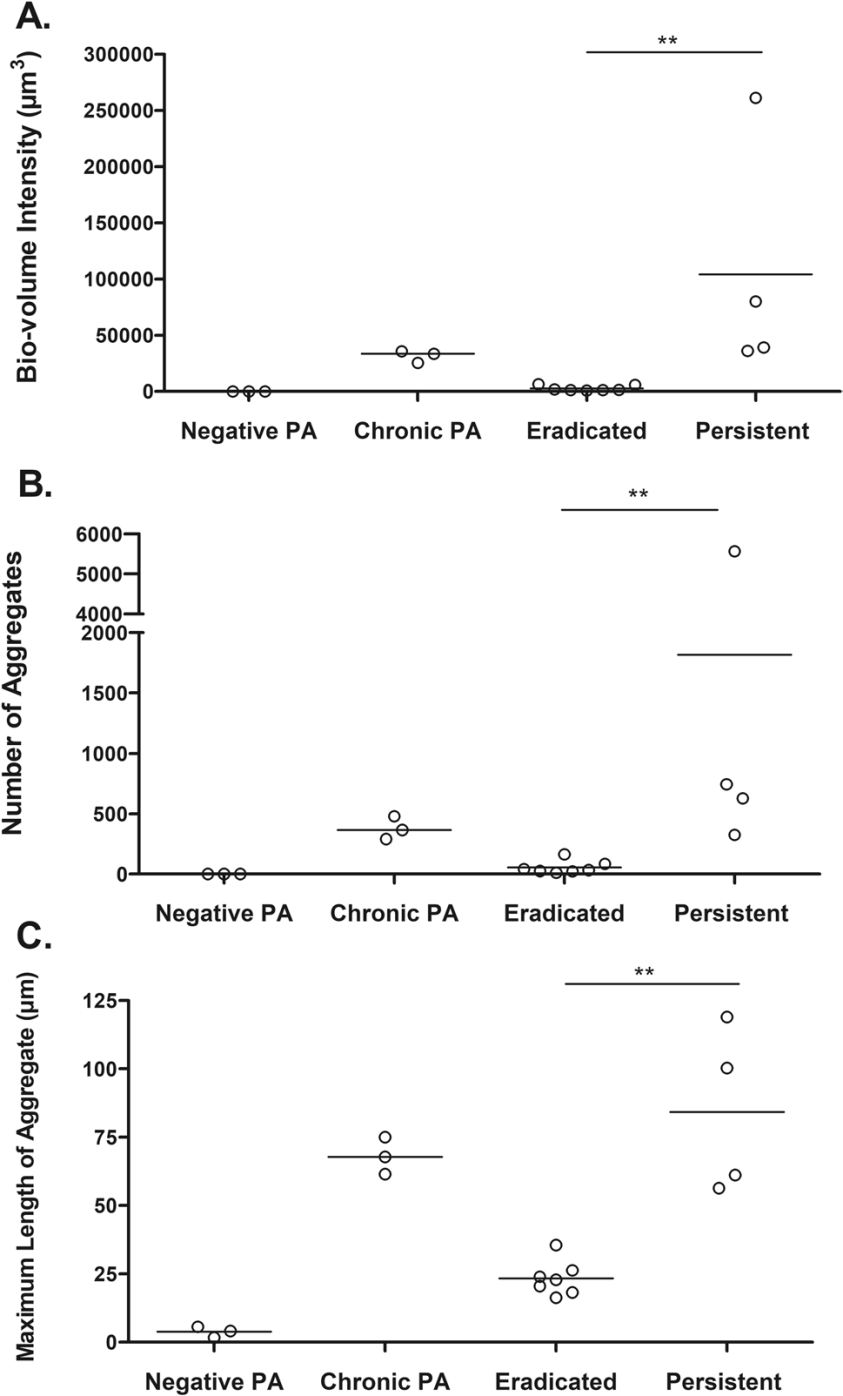
Patients with persistent infection had greater *P. aeruginosa* biovolume and greater number of larger aggregates compared to those with eradicated infection. *P. aeruginosa* visualization in initial sputum sample from negative controls (Negative PA, n = 3), positive controls (Chronic PA, n = 3), CF children who eradicated infection (Eradicated, n = 7) and CF children who developed persistent infection (Persistent, n = 4). Represented median (horizontal line) *P. aeruginosa* (**A**) average biovolume intensity (µm^3^)/sputum slice, (**B**) average number of aggregates/sputum slice, (**C**) average maximum aggregate size (µm)/sputum slice. **p < 0.01 using Mann–Whitney test.

**Figure 2:**
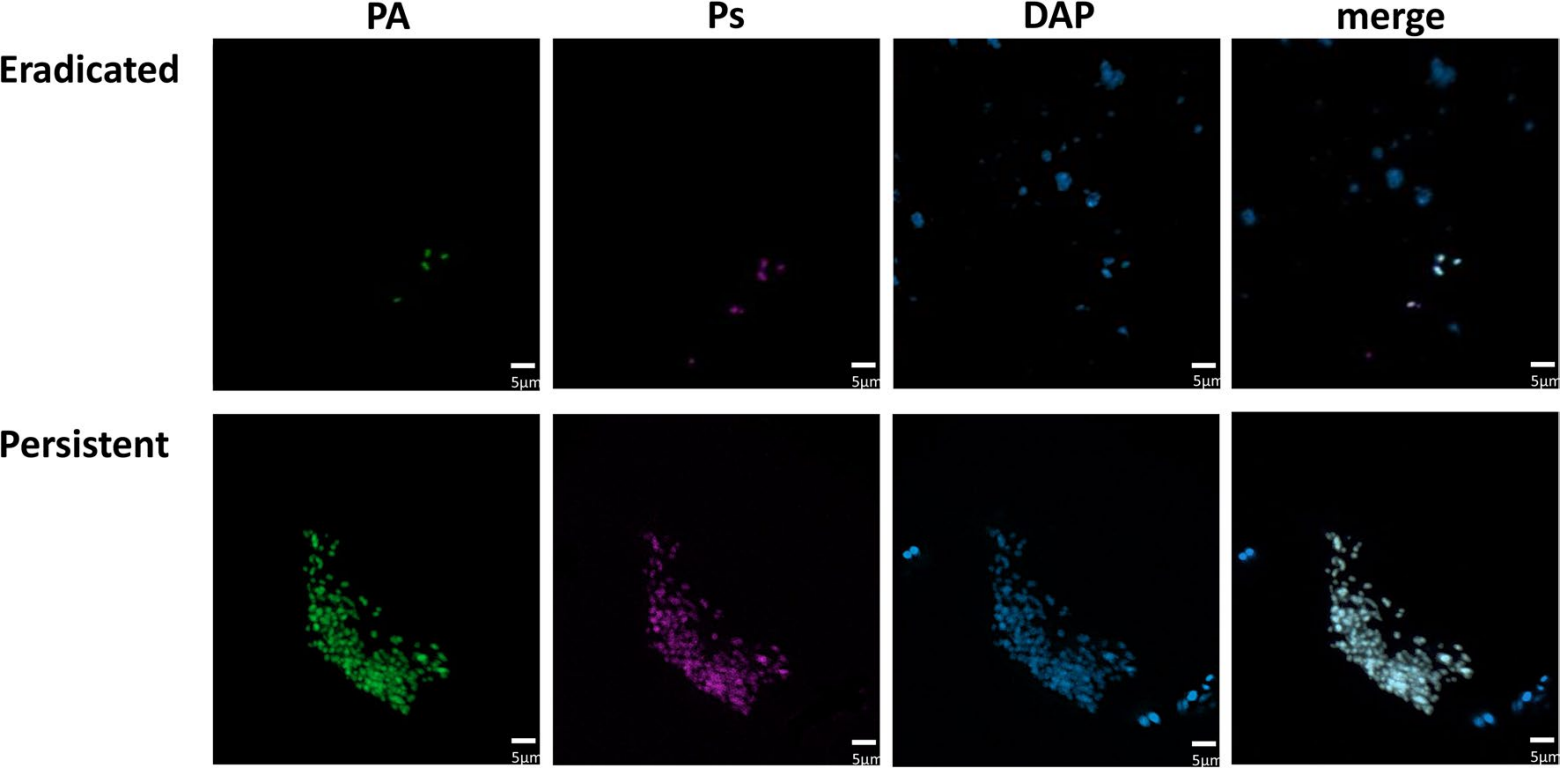
Representative 3D confocal images of *P. aeruginosa* in the sputum of CF patients showing more numerous, larger aggregates in those with persistent compared to eradicated infection. *P. aeruginosa* structural differences are shown using a fluorescently-labelled molecular probe specific for *P. aeruginosa* (green), anti-Psl mAb (magenta), DAPI nucleic acid stain (blue) molecular probes and merged image, with ×100 oil objective lens.

A sputum sample was obtained from each enrolled patient prior to initiation of inhaled tobramycin treatment, and *P. aeruginosa* was labelled using fluorescence in situ hybridization (FISH) and visualized using confocal microscopy. There was an increased *P. aeruginosa* biovolume (per sputum slice) as well as an increased number of larger *P. aeruginosa* aggregates in the sputum of CF children who subsequently failed eradication therapy compared to those who successfully cleared infection (Fig. 1). Representative images are shown in Fig. 2. The amount of *P. aeruginosa* in each sputum sample was also quantified using quantitative polymerase chain reaction (qPCR); *P. aeruginosa* concentrations as measured by qPCR did not correlate with *P. aeruginosa* biovolume visualized directly in fixed sputum specimens (Supplementary Fig. 1). Similarly, there was no correlation between sputum *P. aeruginosa* biovolume and semi-quantitative measures of *P. aeruginosa* growth on plating of the original sputum sample in the clinical microbiology laboratory (data not shown).

**Figure 3:**
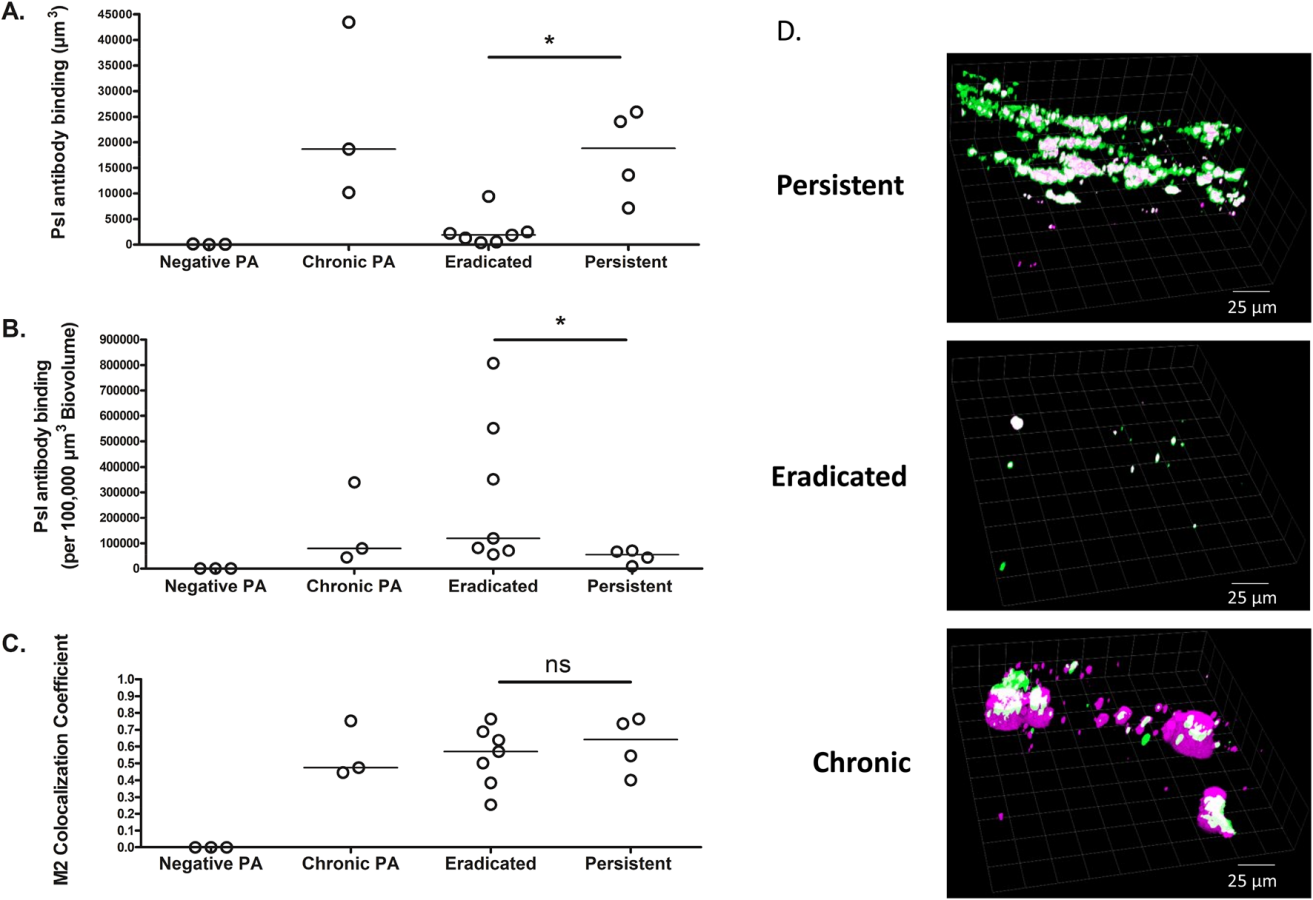
Greater overall anti-Psl antibody staining of *P. aeruginosa* in sputum from patients with persistent compared to eradicated infection. Initial sputum sample from negative controls (Negative PA, n = 3), positive controls (Chronic PA, n = 3), children with CF for whom infection was eradicated (Eradicated, n = 7) and children with CF who developed persistent infection (Persistent, n = 4). Horizontal line indicates the median. (**A**) average anti-Psl antibody binding intensity (µm^3^)/sputum slice, (**B**) average Psl antibody binding per 100,000 µm^3^ *P. aeruginosa* biovolume/sputum slice, (**C**) average proportion of anti-Psl antibody co-localized with *P. aeruginosa* represented by the M2 coefficient, (**D**) representative images from sputum from a patient with Persistent, Eradicated and Chronic infection, green: *P. aeruginosa*, magenta: anti-Psl antibody, white: Psl antibody co-localized with *P. aeruginosa.* *p < 0.05, ns: not significant using Mann–Whitney test.

*Pseudomonas aeruginosa* Psl was also visualized within sputum using fluorescently labelled anti-Psl antibody. Figure 3A shows that the amount of Psl antibody binding in sputum was greater in those who developed persistent infection compared to those who eradicated infection. However, once corrected for increased *P. aeruginosa* biovolume, Psl antibody binding per 100,000 µm^3^ *P. aeruginosa* biovolume was slightly higher in the eradicated compared to persistent group (p = 0.04, Fig. 3B). The proportion of anti-Psl antibody co-localized with *P. aeruginosa* (represented by the M2 coefficient) was similar in both the eradicated and persistent group (Fig. 3C). Figure 3D shows representative images of each category of sputum sample.

### Aggregation and antimicrobial resistance of P. aeruginosa isolates in glass slide chamber

**Figure 4:**
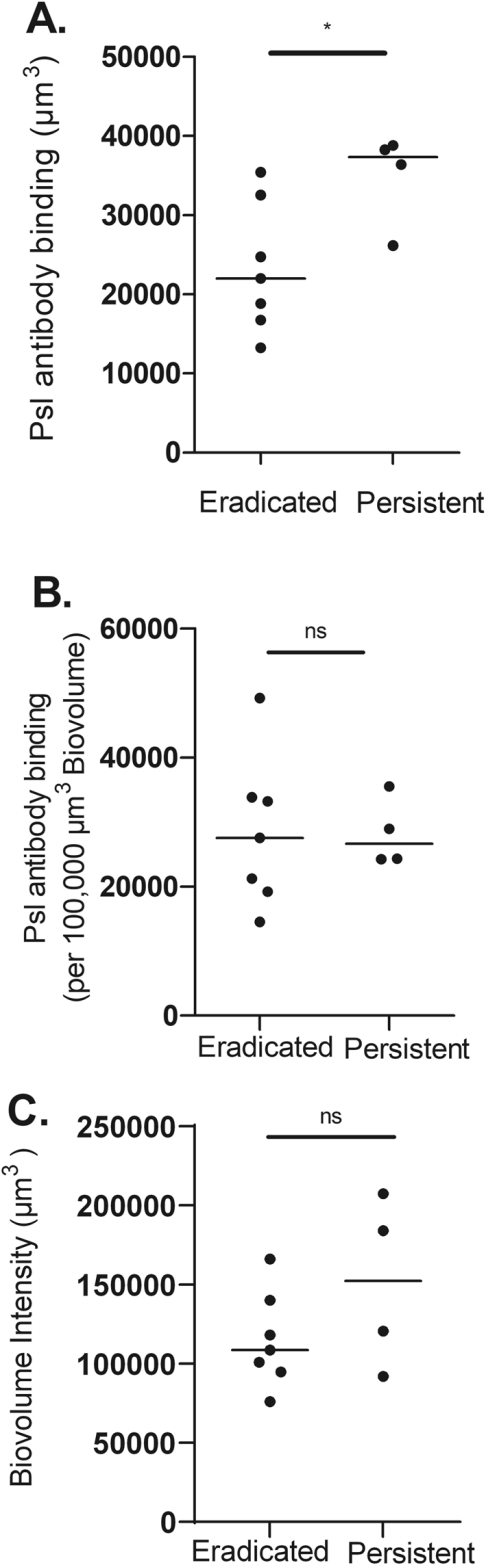
Greater overall anti-Psl antibody staining in glass slide chamber of *P. aeruginosa* isolates from patients with persistent compared to eradicated infection. Eradicated (n = 7) and persistent (n = 4) *P. aeruginosa* isolates grown for 48 h as biofilms in glass slide chamber and then labelled with fluorescent anti-Psl antibody for 3 h then visualized with confocal microscopy. Horizontal line, median. (**A**) average anti-Psl antibody binding intensity (µm^3^)/well, (**B**) average Psl antibody binding per 100,000 µm^3^ *P. aeruginosa* biovolume/well, (**C**) average *P. aeruginosa* biovolume intensity (µm^3^)/well. *p < 0.05, ns: not significant using Mann–Whitney test.

To determine whether these *P. aeruginosa* characteristics observed in children with CF could be replicated in vitro, we then grew the initial *P. aeruginosa* isolates obtained from these patients for 48 h as biofilms in our glass slide chamber model. As with *P. aeruginosa* visualized within sputum, Psl antibody binding (per well) was greater in isolates from children who developed persistent infection compared to isolates from those who eradicated infection (Fig. 4A). However, once corrected for *P. aeruginosa* biovolume, Psl antibody binding per 100,000 µm^3^ *P. aeruginosa* biovolume was not different between persistent and eradicated isolates (Fig. 4B). Although the median *P. aeruginosa* biovolume in the slide chambers after 48 h of growth was greater for persistent compared to eradicated isolates, this difference was not statistically significant (Fig. 4C).

**Figure 5:**
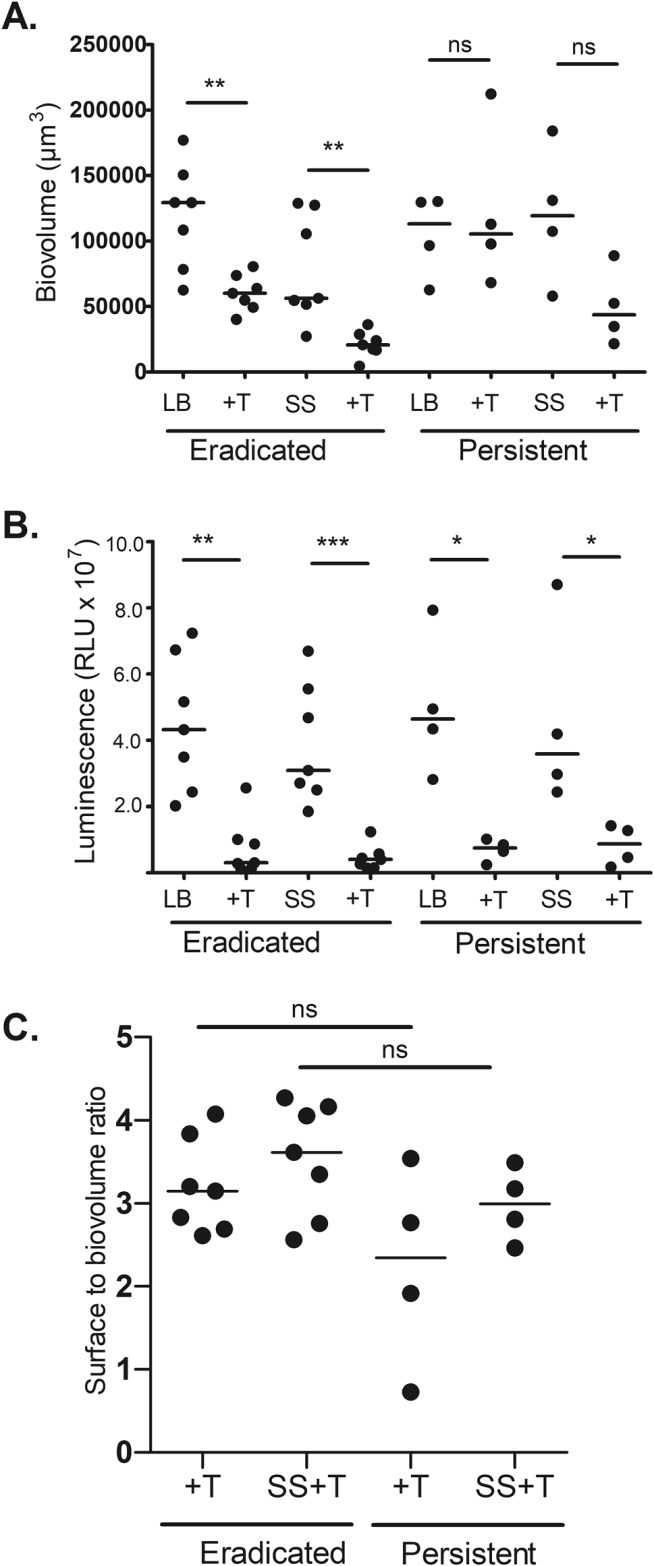
Differences in tobramycin response and aggregation between persistent and eradicated *P. aeruginosa* isolates in glass slide chamber. Eradicated (n = 7) and persistent (n = 4) *P. aeruginosa* isolates grown for 24 h as biofilms in glass slide chamber and then treated with 1000 µg/mL tobramycin for an additional 24 h then visualized with confocal microscopy. LB: Lysogeny Broth alone; + T: plus Tobramycin; SS: in presence of 5% pooled CF Sputum Supernatant. Horizontal line, median. (**A**) average *P. aeruginosa* biovolume intensity (µm^3^)/well, (**B**) average luminescence in relative light units (RLU) of ATP assay/well, (**C**) average degree of aggregation measured by surface to biovolume ratio/well. *p < 0.05, **p < 0.01, ***p < 0.001, ns: not significant using Mann–Whitney test.

Given that persistent isolates failed to be cleared by tobramycin therapy in our patients, we aimed to determine whether this tobramycin tolerance could be measured in our in vitro model. Initial *P. aeruginosa* isolates from patients who subsequently developed persistent (persistent isolates) or eradicated infection (eradicated isolates) were grown for 24 h as biofilms in glass slide chambers and then exposed to high-dose (1000 µg/ml) tobramycin for 24 h. Tobramycin significantly reduced the biovolume of eradicated isolates but not persistent isolates (Fig. 5A). To replicate the CF lung environment, isolates were also grown in the presence of 5% pooled CF sputum supernatant and then treated with tobramycin; again, the biovolume of eradicated isolates was significantly reduced and there was a nonsignificant trend (p = 0.05) to significant reduction in the persistent isolates (Fig. 5A). The metabolic activity of isolates was also measured using an ATP assay and demonstrated significant reduction with tobramycin treatment for eradicated and persistent isolates in both growth conditions (Fig. 5B). The degree of bacterial aggregation (as measured by surface to biovolume ratio) was not different for eradicated and persistent isolates (Fig. 5C).

## Discussion

To our knowledge, this study is the first to demonstrate that children with CF who fail to eradicate new-onset *P. aeruginosa* infection with tobramycin therapy are infected with more numerous, larger aggregates of *P. aeruginosa* expressing increased Psl due to the increased biovolume of *P. aeruginosa*. In vitro*,* increased anti-Psl antibody binding was also seen in persistent compared to eradicated isolates but once corrected for biovolume, this difference was not significant; tobramycin resistance and greater bacterial aggregation was not consistently observed in persistent isolates in our glass slide chamber model, highlighting the importance of direct in vivo observation of *P. aeruginosa* pulmonary infections in CF patients.

Prior studies, including ours, have attempted to identify *P. aeruginosa* characteristics associated with failure of AET in CF. Phenotypes associated with chronic infection, such as mucoidy status and wrinkly phenotypes typical of biofilm producers, have previously been shown to have a weak association with failure of AET^16,17^. Surprisingly, an increased amount of *P. aeruginosa*, as measured by sputum bacterial density counts, for instance, has not been identified as a risk factor for inhaled tobramycin eradication treatment failure. Sputum bacterial density measurements are not performed routinely by the clinical microbiology laboratory for unexpected incident infections and more importantly, are based on planktonic growth of homogenized, serially diluted and plated sputum samples. The increased *P. aeruginosa* biovolume identified in our patients who went on to develop persistent infection was only noted on direct visualization of their embedded and fixed sputum samples. Sputum *P. aeruginosa* biovolume did not correlate with either semi-quantitative measures of growth on plates or *P. aeruginosa* concentrations measured in sputum by PCR.

Through direct visualization of *P. aeruginosa* within sputum, we were also able to observe more numerous, larger aggregates in those who failed AET. Bacterial aggregation is a well-recognized mechanism of antibiotic tolerance and immune evasion^18^. There are many factors that contribute to *P. aeruginosa* aggregation within CF airways. Intrinsic bacterial characteristics include the expression of Psl. Our collaborators have previously shown that *P. aeruginosa* aggregates in CF sputum express Psl and that such aggregation likely occurs via an EPS-mediated polymer-bridging mechanism^19^. We noted increased anti-Psl antibody binding both in vivo (in patient sputum) as well as in vitro (isolates cultured in the glass slide chamber model) for patients with CF with persistent compared to eradicated infection. However, in contrast to our previous publication, once adjusted per 100,000 µm^3^ of *P. aeruginosa* biovolume, this difference was no longer significant, suggesting that persistent isolates do not have increased expression of Psl per cell, rather there were more bacterial cells, each expressing the same amount of Psl, in persistent compared to eradicated isolates^6^. In addition to specific *P. aeruginosa* characteristics, there are extrinsic factors, conditions in the CF airway, that promote bacterial aggregation. For example, Singh et al. demonstrated that secreted neutrophil elastase in sputum supernatant can cleave *P. aeruginosa* flagella, decreasing motility and triggering aggregation^20^. Of note, this aggregation can occur despite mutations in biofilm related genes, underscoring the difference between aggregation and biofilm formation. The composition of sputum itself can also affect aggregation as mucin has been shown to enhance aggregation as has the presence of host polymers, via a depletion mechanism^21,22^. Despite growing our persistent isolates in vitro in pooled CF sputum supernatant (comprising of excreted cellular products but without host or other bacterial cells), we could not replicate the increased aggregation observed in the sputum of patients who developed persistent infection. Similarly, exposure of both eradicated and persistent isolates to tobramycin led to a decrease in metabolic activity, which may represent a bacterial protective response^23^. Direct analysis of the composition of the sputum from our enrolled patients was beyond the scope of this study, however, future investigation in this area is warranted.

This study used MiPACT to directly visualize bacterial communities in airways^24^. Other investigators have used these methods to describe the composition of airway infections in patients with CF, with chronic obstructive lung disease (COPD) and with ventilator associated pneumonia (VAP)^15,25^. The current study, however, is the first to use MiPACT to identify differences in bacterial characteristics that correlate with response to antibiotic therapy. This is of particular importance to CF patients as there has been no test developed to date that can predict clinical outcomes of antibiotic treatment^14^. The complexities of the CF lung environment, with its unique growth conditions, polymicrobial interactions and drug delivery challenges, are very difficult, if not impossible, to replicate in the laboratory^13^. Even in our glass slide chamber model based on biofilm growth and incorporating host factors in the form of CF sputum supernatant, we were unable to consistently demonstrate tobramycin tolerance and bacterial aggregation by persistent isolates in vitro. Our prior in vitro studies demonstrating tobramycin tolerance and greater aggregation in persistent *P. aeruginosa* isolates only did so with the addition of *Staphylococcus aureus* filtrate, SpA or anti-Psl antibody, the clinical relevance of which is unclear^6,26^. The use of the MiPACT methodology to visualize other bacterial species and host immune cells as well as their geospatial interaction has the potential to further our understanding of more complicated CF infections such as those associated with pulmonary exacerbations^15,27^.

There were several limitations to this study. The patient sample size was small and included only older patients who could produce sputum, limiting the generalizability of the findings to CF children with new-onset *P. aeruginosa* infection detected from throat swabs. Furthermore, obtaining sputum samples has become more difficult with the introduction of CFTR modulator therapy, reducing the ability of CF patients to expectorate^28^. Despite the small number of patients enrolled, however, statistically significant differences in *P. aeruginosa* aggregate formation were demonstrated in sputum between the patient groups. In addition, due to the smaller volume of sputum produced by children, there was not enough sample to perform serial dilutions to calculate sputum bacterial density [in colony forming units (CFU)/ml]. However, semi-quantitative measures of *P. aeruginosa* load identified by plating of the original sputum sample in the clinical microbiology laboratory were available for comparison. Finally, this study focused on characteristics of only *P. aeruginosa* itself associated with failure of AET but there are likely other factors, such as interactions with other organisms and host responses, that play a role^26^. Other investigators have demonstrated that polymorphonuclear leukocytes respond aggressively toward a biofilm matrix consisting of both Psl and alginate exopolysaccharides, responses not considered in our current study^29^.

In conclusion, this study demonstrated that children with CF who failed to eradicate *P. aeruginosa* respiratory infection with inhaled tobramycin were infected with Psl-expressing *P. aeruginosa* that form more numerous and larger aggregates than in those who successfully clear their infection. These groups of patients could not reliably be distinguished using standard in vitro methods, highlighting the need for direct visualizations of infections within airways to predict antibiotic response in CF.

## Methods

### Study design and patient population

This was a prospective, observational study conducted at SickKids (Toronto, Canada) from March 2020–2022. Children with CF were enrolled if they had a new onset *P. aeruginosa* infection defined as a sputum culture positive for *P. aeruginosa* with at least 3 preceding negative cultures in the prior 12 months^16,30^. After enrollment, a sputum culture was obtained prior to starting inhaled tobramycin for 28 days. Per the SickKids AET protocol, if the sputum culture obtained 1 week following the end of inhaled tobramycin treatment was still positive for *P. aeruginosa*, an additional 28 days of inhaled tobramycin was prescribed; if the sputum culture was still positive for *P. aeruginosa* following the second round of inhaled tobramycin, the patient was admitted to hospital and intravenous tobramycin and ceftazidime was prescribed for 2 weeks followed by an additional 28 days of inhaled tobramycin^31^.

AET failure was defined as a *P. aeruginosa* positive sputum culture in the 6 months following the initial positive culture; all enrolled patients had a minimum of 6 months of follow up time in the study. If a patient failed AET, the initial *P. aeruginosa* isolate (obtained prior to commencing inhaled tobramycin) was defined as persistent; otherwise, the initial isolate was defined as eradicated.

Negative sputum controls that were negative for *P. aeruginosa* by qPCR were included from CF children who had never grown *P. aeruginosa* in their respiratory tract. Positive sputum controls that were positive for *P. aeruginosa* by qPCR were included from CF children with chronic *P. aeruginosa* infection^32^. DNA extraction and qPCR was performed as described previously^30^.

### Sputum processing and imaging

Sputum processing was performed using an adapted MiPACT (tissue clearing) technique, previously described^15,24^. In brief, expectorated sputum specimens were collected from enrolled patients in a sterile cup and immediately stored at 4 °C. Within 24 h of collection, sputum plugs (approximately 0.2 g) were fixed in 4% paraformaldehyde (PFA), washed with 1× phosphate-buffered saline (PBS) then sectioned into small slices (approximately 5 mm diameter). Individual sputum slices were added to separate wells of a Nunc™ Lab-Tek™ eight-chamber cover glass slide (Thermo Fisher Scientific, Mississauga, ON), consisting of 250 µL hydrogel mixture. The sputum-hydrogel mixture (within the chamber wells) was sealed inside a BD GasPak™ EZ container (VWR, Mississauga, ON) with an anaerobic pack, then allowed to polymerize for 3 h, at 37 °C. Following polymerization, the solidified sample was cleared in 8% sodium dodecyl sulfate (SDS) solution (adjusted to pH 8) for 3–5 days, at 37 °C. After clearance, the sputum hydrogels were washed with 1× PBS and stored in 0.01% (wt/vol) sodium azide solution, at 4 °C until imaging.

To visualize *P. aeruginosa* and the exopolysaccharide, Psl, within the sputum hydrogels, a fluorescent in situ hybridization (FISH) technique was performed, as previously described^24^ with minor adjustments. In brief, sputum hydrogels were sectioned into three thin slices (approximately 1 mm thickness ea.), then added to 500 µL of hybridization buffer with 4 µL of 100 µM PsearA-Alexa488 (green) probe. The PsearA probe, targeting the 16S rRNA gene fragment of *P. aeruginosa*, was allowed to incubate with the sputum hydrogels for 18–24 h, at 46 °C. The sputum hydrogels were rinsed in a wash buffer, then incubated in 1 mL of fresh wash buffer for 6 h, at 48 °C. Following DNA-probe hybridization, sputum hydrogels were washed with 1× PBS and incubated in 2% bovine serum albumin (BSA) solution for 18–24 h, in dark, at room temperature. The next day, sputum hydrogels were removed and added to 500 µL of fresh 2% BSA solution with 4 µL of 2 mg/mL Psl0096-Alexa594 (red) anti-Psl antibody, then incubated for 6 h, in dark, at room temperature. The sputum hydrogels were washed with 1× PBS, then incubated in 250 µL of refractive index matching solution (RIMS) with 4 µL of 10 µg/mL DAPI, in dark, at room temperature with gentle rotation. The sputum hydrogel slices were mounted on a microscope slide and sealed with a 1.7 mm CoverWell™ Perfusion Chamber (Sigma-Aldrich, Oakville, ON).

Images used for analysis were acquired using a Quorum Wave FX Borealis Spinning Disk Confocal Microscope, based on a Yokohama CSU-10 scan head. Fluorescently labeled (red) Psl0096 antibody, PsearA *P. aeruginosa* (green) probe and DAPI (blue) nucleic acid stain were excited using 561, 491 and 405 nm excitation lines, respectively. The sputum hydrogels were visualized with a 20×/0.75 Zeiss lens, coupled to a 1.6 × magnification coupler. Image acquisition was performed using the Quorum Volocity 6.3 software. A total of 6 z-stack images were acquired per slide in increments of 0.3 µm. All image processing of hydrogel z-stack images was performed using Volocity. *P. aeruginosa* colonization within sputum was quantified using the biovolume intensity, maximum object size (minimum measurement considered as an aggregate set as 2 µm^3^) and population number functions. Psl antibody binding within *P. aeruginosa* biofilms was calculated using the total voxel ratio of the red to green channel. Last, the Manders (M2) coefficient was selected to calculate the degree of Psl to PA co-localization. All images are included in the Supplemental materials as Supplementary Fig. 2.

Three dimensional representative images at 100 × oil immersion were acquired using a Leica SP8 Lightning Confocal STED microscope. The PsearA *P. aeruginosa* (green) probe, Psl0096 antibody (magenta) and DAPI (blue) nucleic acid stain were excited using 592, 488 and 405 nm excitation lines, respectively. The sputum hydrogels were visualized with a 100×/1.4 (STED) oil lens. Image acquisition and processing was performed using the Leica LAS, Lightning Module, Navigator software. Representative z-stack images of eradicated and persistent *P. aeruginosa* isolates were captured in increments of 0.25 µm.

### Pseudomonas aeruginosa isolate recovery from sputum

A portion of the sputum collected from the study patients was used for *P. aeruginosa* isolate recovery. Sputum specimens were spread plated on MacConkey agar (with 8% crystal violet), then incubated for 48 h at 42 °C. Individual morphotypes were then grown on blood agar for an additional 24 h at 37 °C. Identification of *P. aeruginosa* was confirmed by Matrix-assisted desorption/ionization-time of flight (MALDI-ToF) and the isolates were frozen at − 80 °C until subsequent examination via glass slide chamber model and ATP assay. If the isolate could not be recovered from the sputum sample obtained for MiPACT, the initial *P. aeruginosa* isolate identified by the clinical microbiology laboratory in the initial sputum sample from the patient was recovered for the following experiments.

### Pseudomonas aeruginosa biofilm growth in glass slide chamber and imaging

*Pseudomonas aeruginosa* isolates recovered from the sputum specimens were grown as biofilms using a Nunc Lab-Tek II, 8-chambered cover glass slide (Thermo Fisher Scientific, Mississauga, ON), previously described^33^. In brief, *P. aeruginosa* was grown in LB (optimal for growing biofilms in the glass slide chamber) for 24 h at 37 °C with shaking (225 rpm). The culture was diluted 1:100, then grown to an early log phase of 0.1 OD at 600 nm. Of this culture, 200 µL was added to the wells of a glass slide chamber, then incubated for 24 h at 37 °C. The next day, media was removed and either 200 µL of LB alone or LB with tobramycin (final concentration 1000 µg/mL) was added to designated wells, then incubated for 24 h at 37 °C. Following incubation, media was removed and 200 µL of Syto-9™ live-cell fluorescent stain in LB was added to the wells, then incubated for 45 min. The wells were washed 2× with LB and LB was added back into the wells prior to visualization via confocal laser scanning microscopy (CLSM). This procedure was also replicated in the presence of 5% pooled CF sputum supernatant, based on a modified prior protocol^34,35^; sputum supernatant comprised of excreted cellular products but not host or bacterial cells, given the non-specific binding of Syto-9™ live-cell fluorescent stain. This procedure was repeated to attain 3 biological replicates for each *P. aeruginosa* isolate.

To measure Psl antibody binding, *P. aeruginosa* was grown in the same manner to an early log phase of 0.1 OD at 600 nm, of which 220 µL was added to the wells of an 8-chambered cover glass slide and incubated for 24 h at 37 °C. Following incubation, media was removed and fresh LB was added back into the wells, then allowed to incubate for an additional 24 h. The next day, media was removed and either 200 µL of LB alone or LB with Psl0096-Alexa594 (red) anti-Psl antibody^36^ (final concentration 56 µg/mL) was added to designated wells, then incubated for 90 min at RT. Following incubation, media was removed and 200 µL of Syto-9™ in LB was added to the wells, then incubated for an additional 45 min. The wells were washed 2× with LB before CLSM in LB.

Quantification of biovolume intensity and Psl antibody binding within *P. aeruginosa* biofilms was calculated, as previously described herein, using Volocity. To measure *P. aeruginosa* aggregation, surface-to-biovolume ratio was quantified using Comstat2 software as a plugin to ImageJ, as previously described^33^.

### ATP assays of P. aeruginosa biofilms

Cell metabolic activity of *P. aeruginosa* biofilms was assessed using an adapted ATP assay, previous described^6,35^. In brief, *P. aeruginosa* was grown overnight in cation-adjusted Muller-Hinton broth (CAMHB) (standardized media for antimicrobial susceptibility testing) at 37 °C with shaking (225 rpm). The overnight culture was diluted 1:100 in CAMHB, then grown to an early log phase of 0.1 OD at 600 nm. Of this culture, 220 µL was added to the wells of a white Greiner Medium Binding 96-well plate (Sigma-Aldrich, Oakville, ON) in absence or presence of 5% sputum supernatant, then incubated for 24 h at 37 °C without shaking. Following incubation, media was removed and 200 µL of CAMHB in absence or presence of sputum supernatant (final sputum dilution, 5%) and tobramycin (final concentration 1000 µg/mL) was added to designated wells, then incubated for 24 h at 37 °C. The next day, media was removed and the wells were washed 2X with CAMHB and 100 µL of CAMHB was added back into the wells. To measure ATP in the cell-attached fraction of the wells, the base of each well was disrupted by scraping and 100 µL of Bac Titer-Glo™ reagent (Promega, Madison, WI) was added. The plates were gently mixed on an orbital shaker for 10 min in the dark before luminescence reading as per the manufacturer's protocol. This procedure was repeated to attain three biological replicates for each *P. aeruginosa* isolate*.*

### Genomic analysis

Whole genome sequencing raw data was trimmed using TRIMMOMATIC with parameters LEADING:3 TRAILING:3 SLIDINGWINDOW:4:15 MINLEN:80. We used SKA to compare samples within patients, before and after treatment. To identify the multi-locus sequence typing (MLST) profiles we used ska *type* function and for single nucleotide polymorphism (SNP) detection and distance estimation between samples, we used ska *fastq*, *compare*, *merge* and *distance* functions. Default parameters were used in every case^37,38^.

### Statistical analysis and institutional approvals

All statistical analyses were done using GraphPad 5.0. Continuous variables were compared using non-parametric Mann–Whitney test; p-value of < 0.05 was considered significant. This study was approved by the SickKids Research Ethics Board (REB#1000079038); written informed consent was obtained from all participants and research conducted in accordance with all relevant guidelines and regulations.

## Supplementary Information

Supplementary Figure 1.

Supplementary Figure 2.

## Data Availability

All images that support the findings of this study are available in the Supplemental materials as Supplementary Fig. 2. All genomic data are available through BioProject ID: PRJNA867399 (http://www.ncbi.nlm.nih.gov/bioproject/867399).
