# Supplementary material for: Pseudomonas aeruginosa aggregation and Psl expression in sputum is associated with antibiotic eradication failure in children with cystic fibrosis
Source: Sci Rep. 2022 Dec 12;12:21444. doi: 10.1038/s41598-022-25889-6 (PMC9744911; doi:10.1038/s41598-022-25889-6)
Supplement: Supplementary file 1 — Supplementary Figure 1. [file 41598_2022_25889_MOESM1_ESM.pdf]

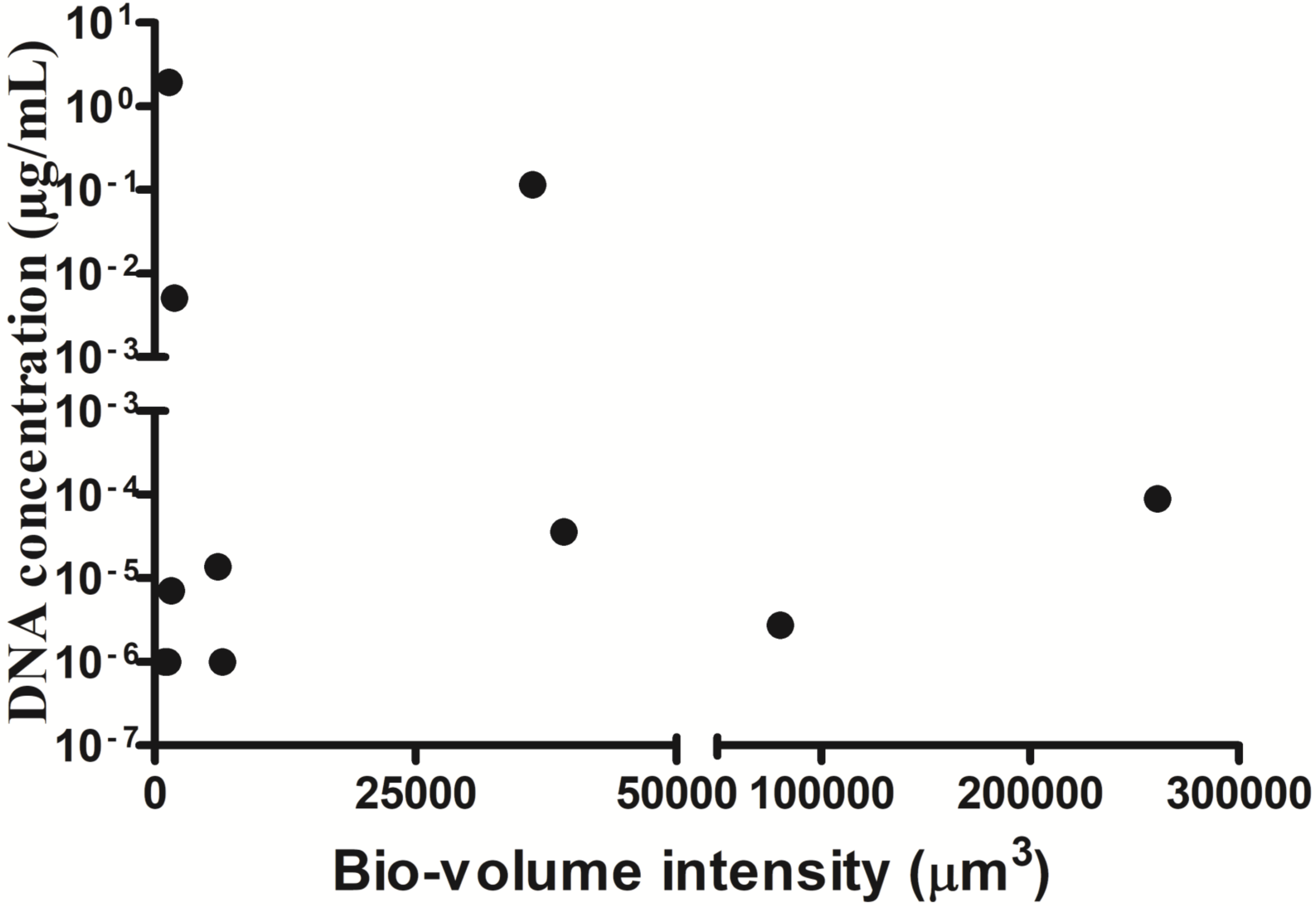

Supplemental Figure 1. Relationship between *P. aeruginosa* biovolume measured in sputum (µm^3) and *P. aeruginosa* DNA concentrations in sputum as measured by qPCR (µg/mL).
