# Supplementary material for: Pseudomonas aeruginosa aggregation and Psl expression in sputum is associated with antibiotic eradication failure in children with cystic fibrosis
Source: Sci Rep. 2022 Dec 12;12:21444. doi: 10.1038/s41598-022-25889-6 (PMC9744911; doi:10.1038/s41598-022-25889-6)

Supplementary Figure 2

Hydrogel model confocal images of eradicated and persistent PA infection, and positive (chronic PA) and negative controls. Images were visualized within the sputum of new onset infected patients with CF prior to AET. PA structural differences are shown in separated channels using fluorescently labelled PA (green), anti-Psl mAb (magenta) and DAPI nucleic acid stain (blue), with 20X objective lens. The white in the combine channels indicates areas of colocalization.

PC001 Slice #1 (Chronic PA Control)

20X Objective

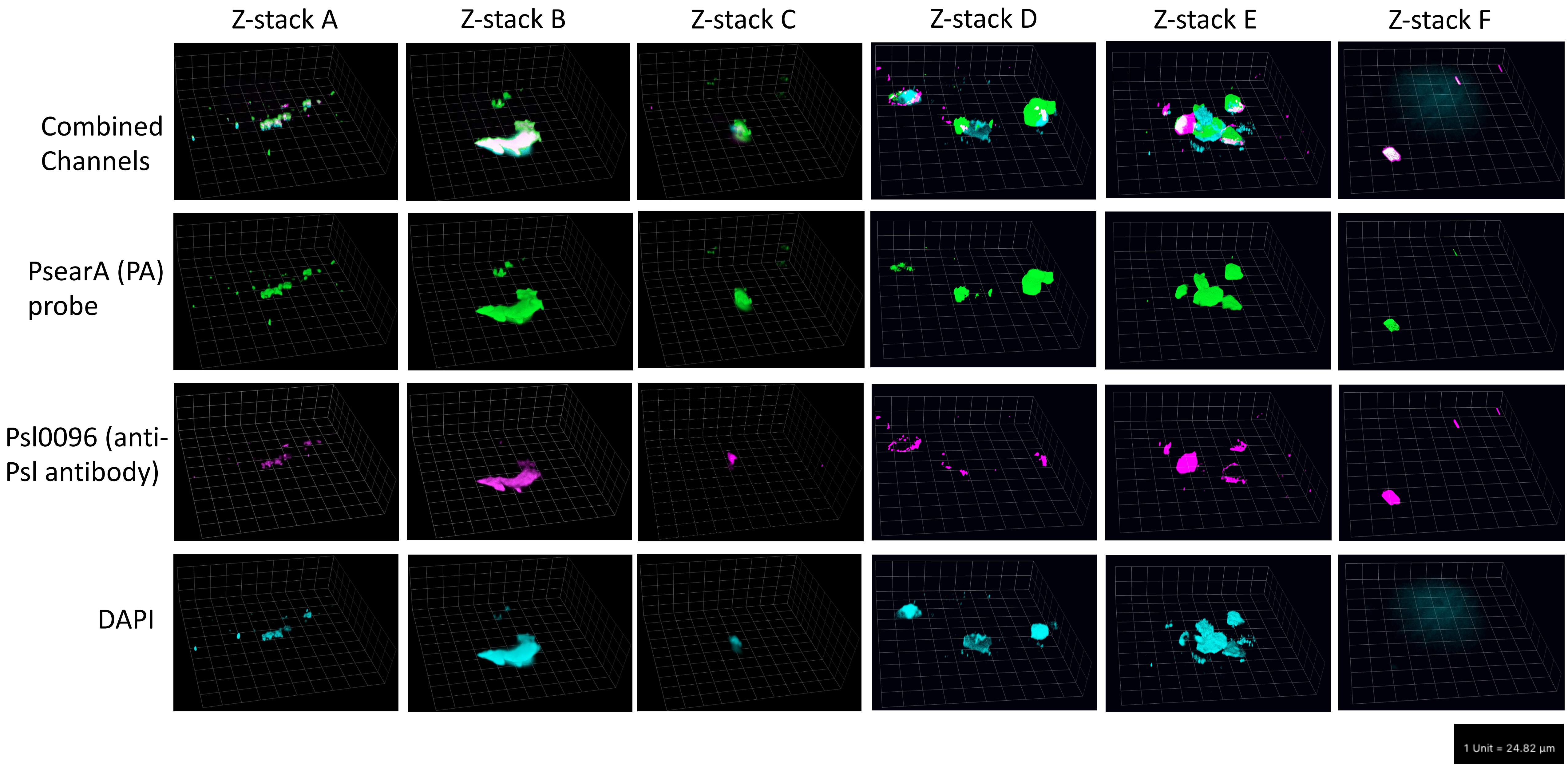

PC001 Slice #2 (Chronic PA Control)

20X Objective

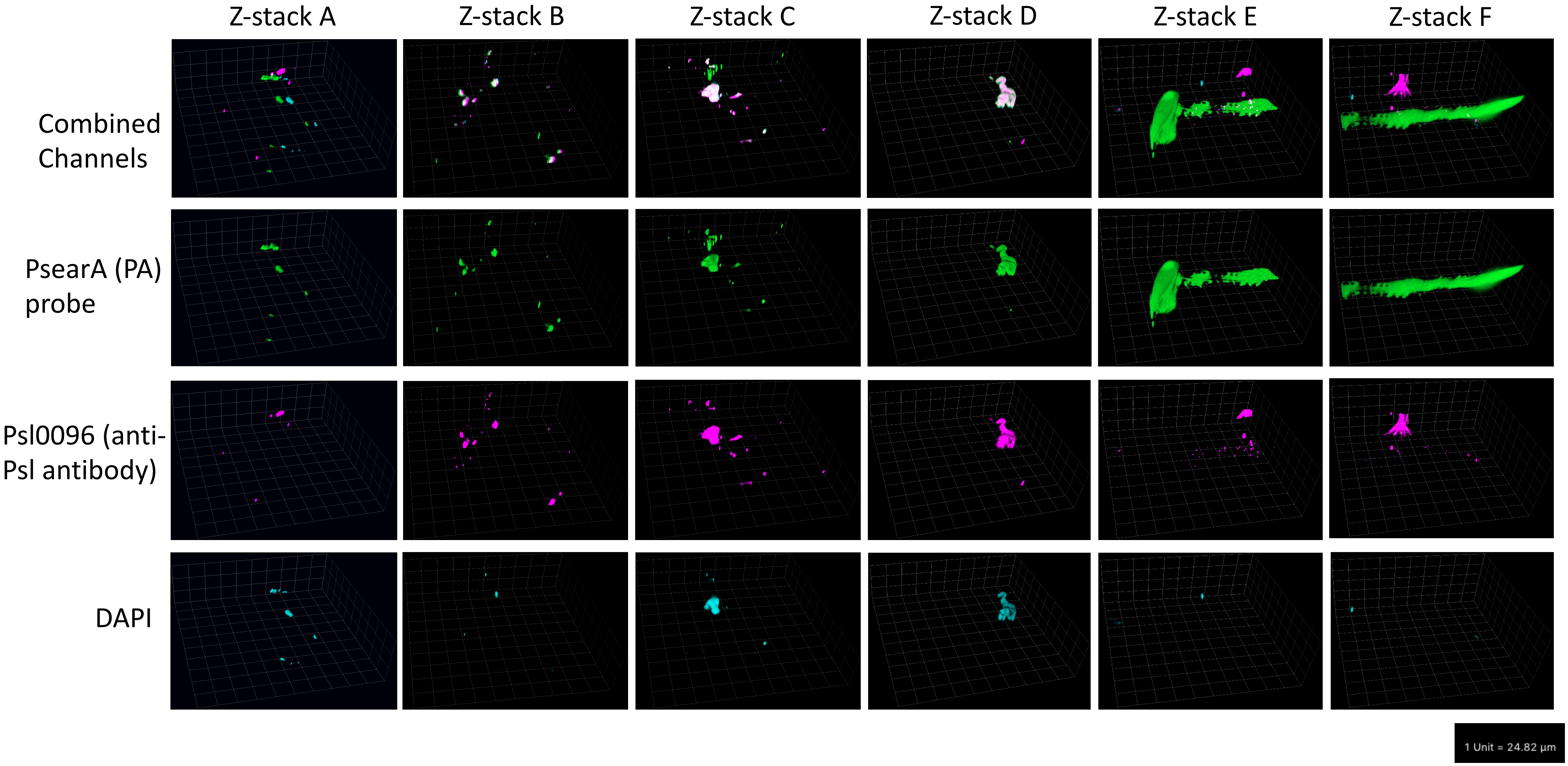

PC001 Slice #3 (Chronic PA Control) 20X Objective

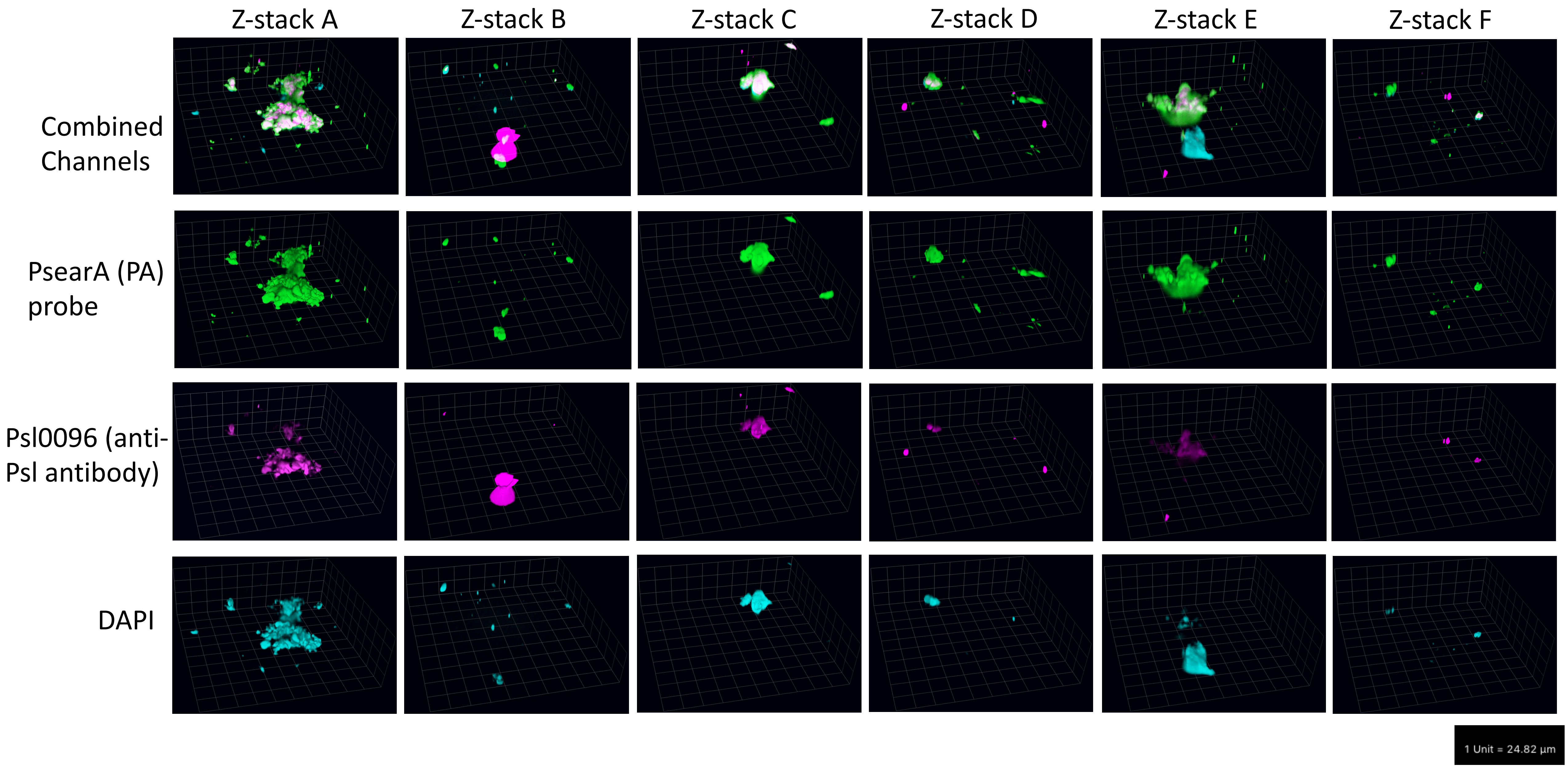

PC004 Slice #1 (Chronic PA Control)

20X Objective

Z-stack A | Z-stack B | Z-stack C | Z-stack D | Z-stack E | Z-stack F

Combined Channels

PsearA (PA) probe

Psl0096 (anti-Psl antibody)

DAPI

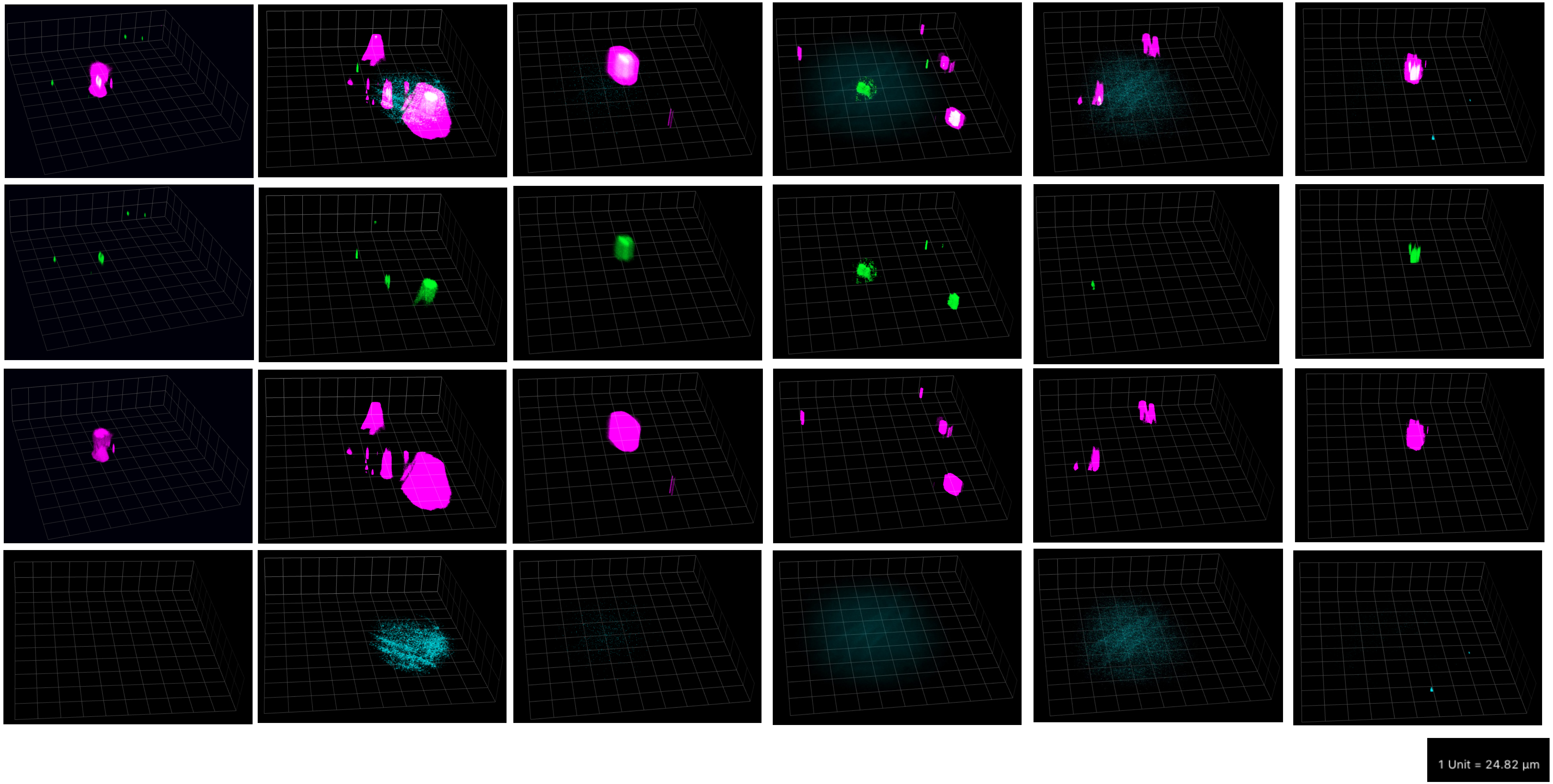

PC004 Slice #2 (Chronic PA Control)

20X Objective

Z-stack A | Z-stack B | Z-stack C | Z-stack D | Z-stack E | Z-stack F

Combined Channels

PsearA (PA) probe

Psl0096 (anti-Psl antibody)

DAPI

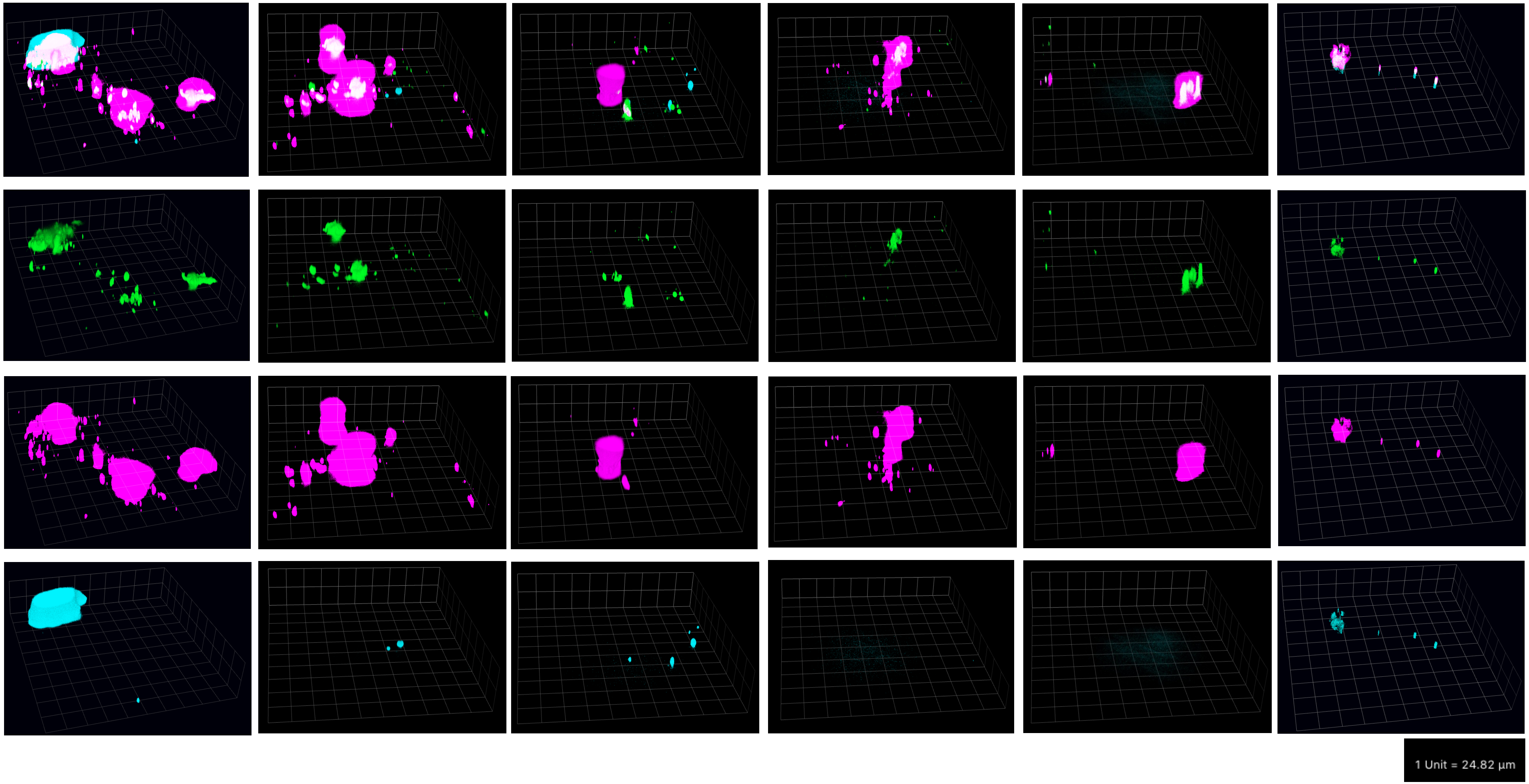

PC004 Slice #3 (Chronic PA Control)

20X Objective

Z-stack A | Z-stack B | Z-stack C | Z-stack D | Z-stack E | Z-stack F

Combined Channels

PsearA (PA) probe

Psl0096 (anti-Psl antibody)

DAPI

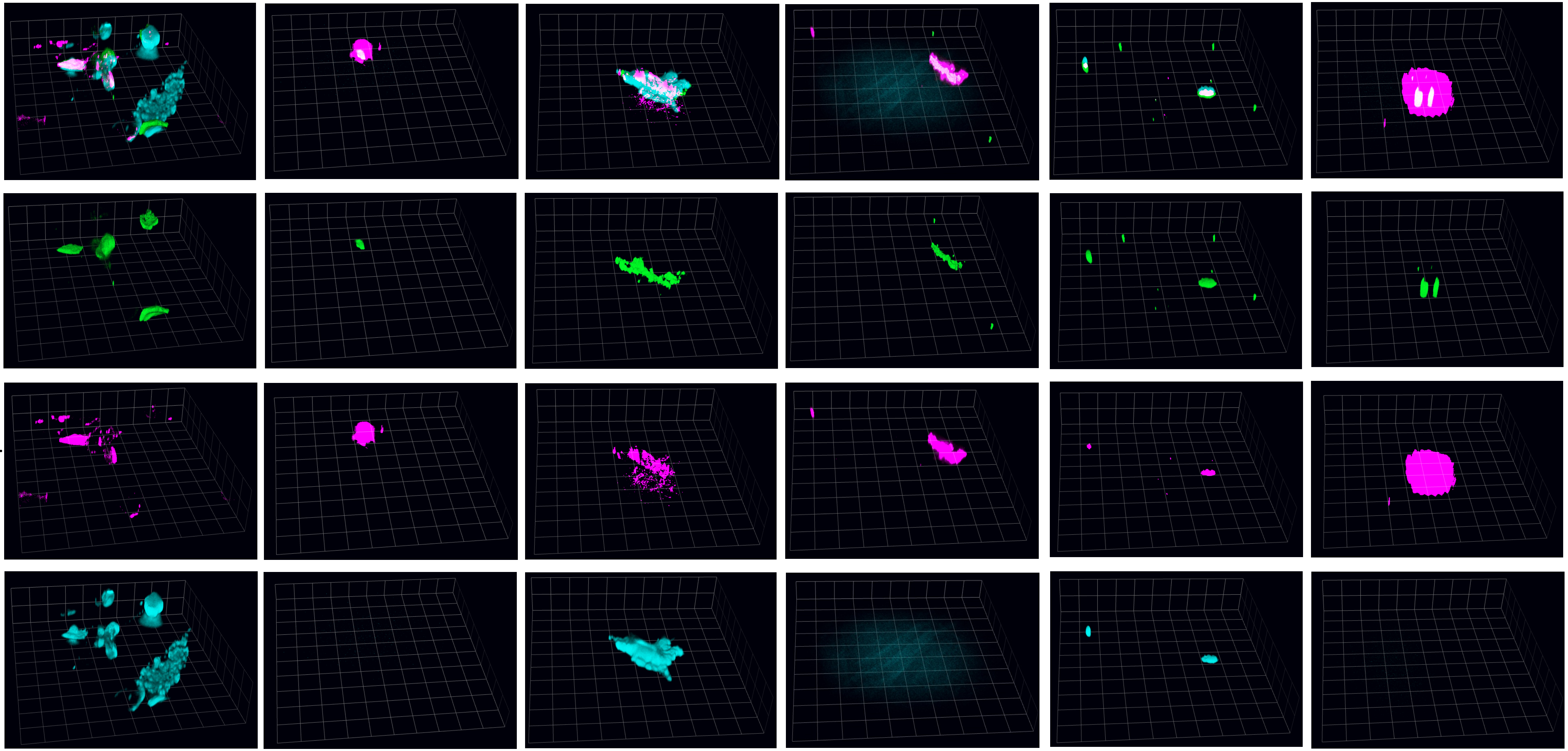

1 Unit = 24.82 µm

PC006 Slice #1 (Chronic PA Control)

20X Objective

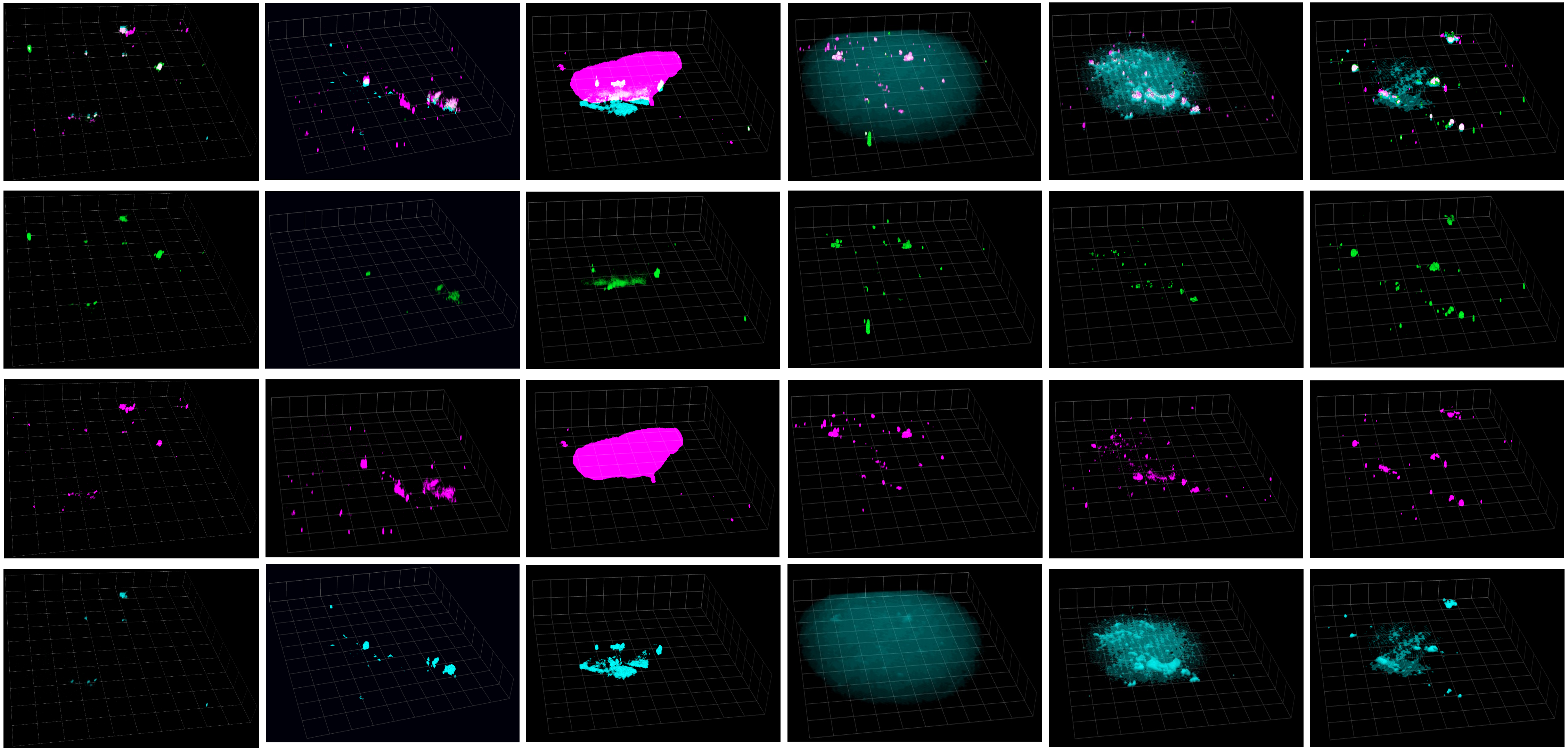

PC006 Slice #2 (Chronic PA Control)

20X Objective

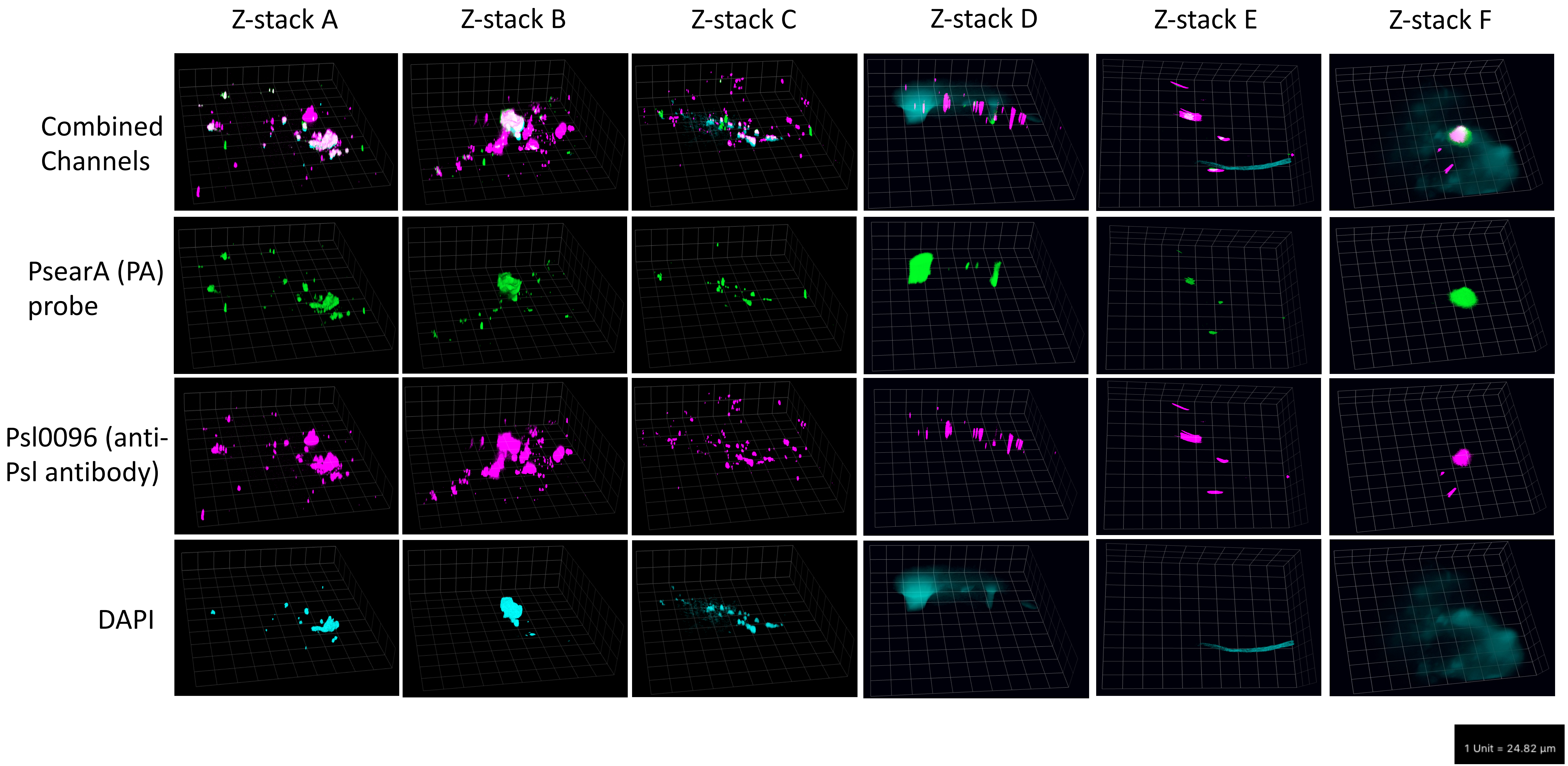

PC006 Slice #3 (Chronic PA Control)

20X Objective

Z-stack A | Z-stack B | Z-stack C | Z-stack D | Z-stack E | Z-stack F

Combined Channels

PsearA (PA) probe

Psl0096 (anti-Psl antibody)

DAPI

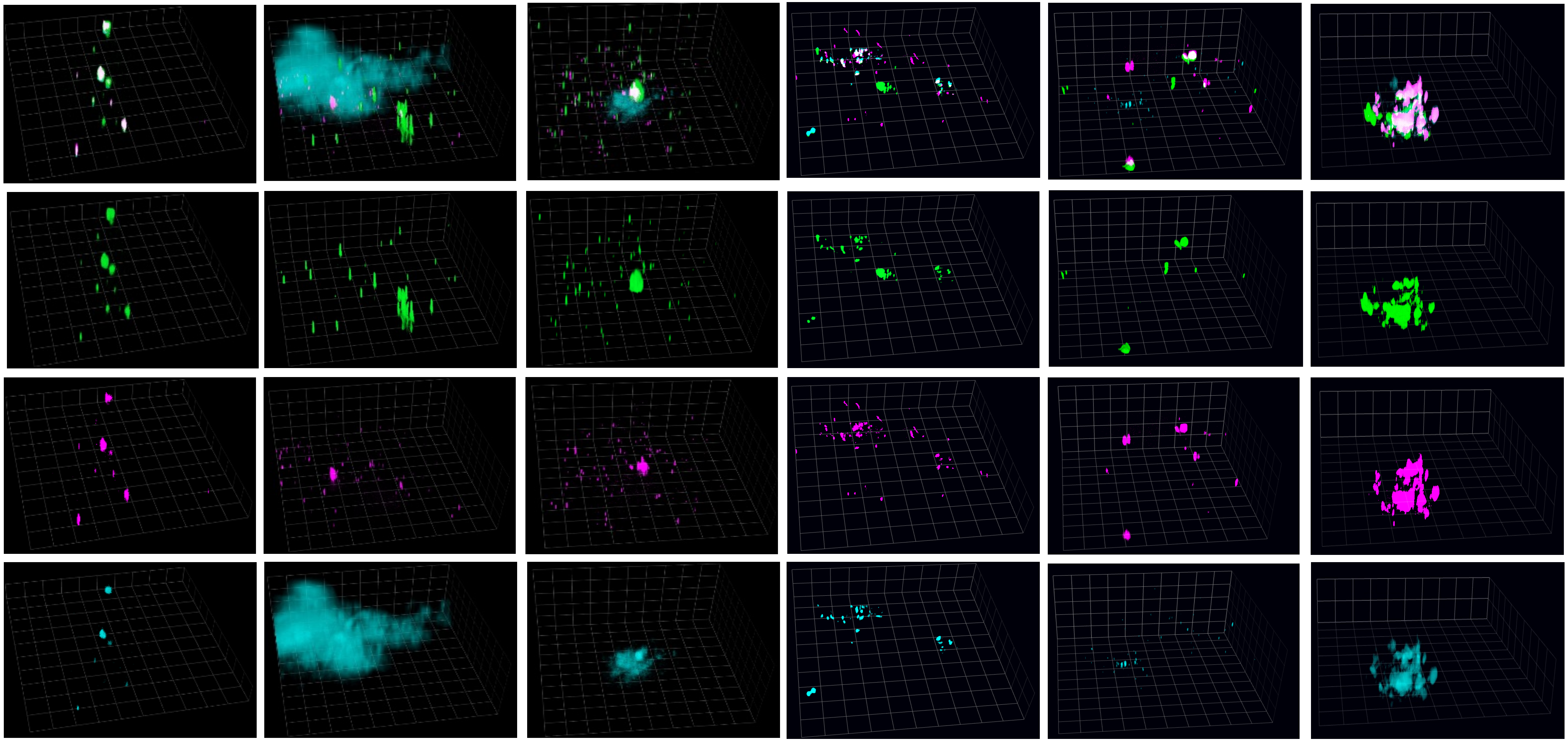

1 Unit = 24.82 µm

NC007 Slice #1 (Negative PA Control)

20X Objective

Z-stack A | Z-stack B | Z-stack C | Z-stack D | Z-stack E | Z-stack F

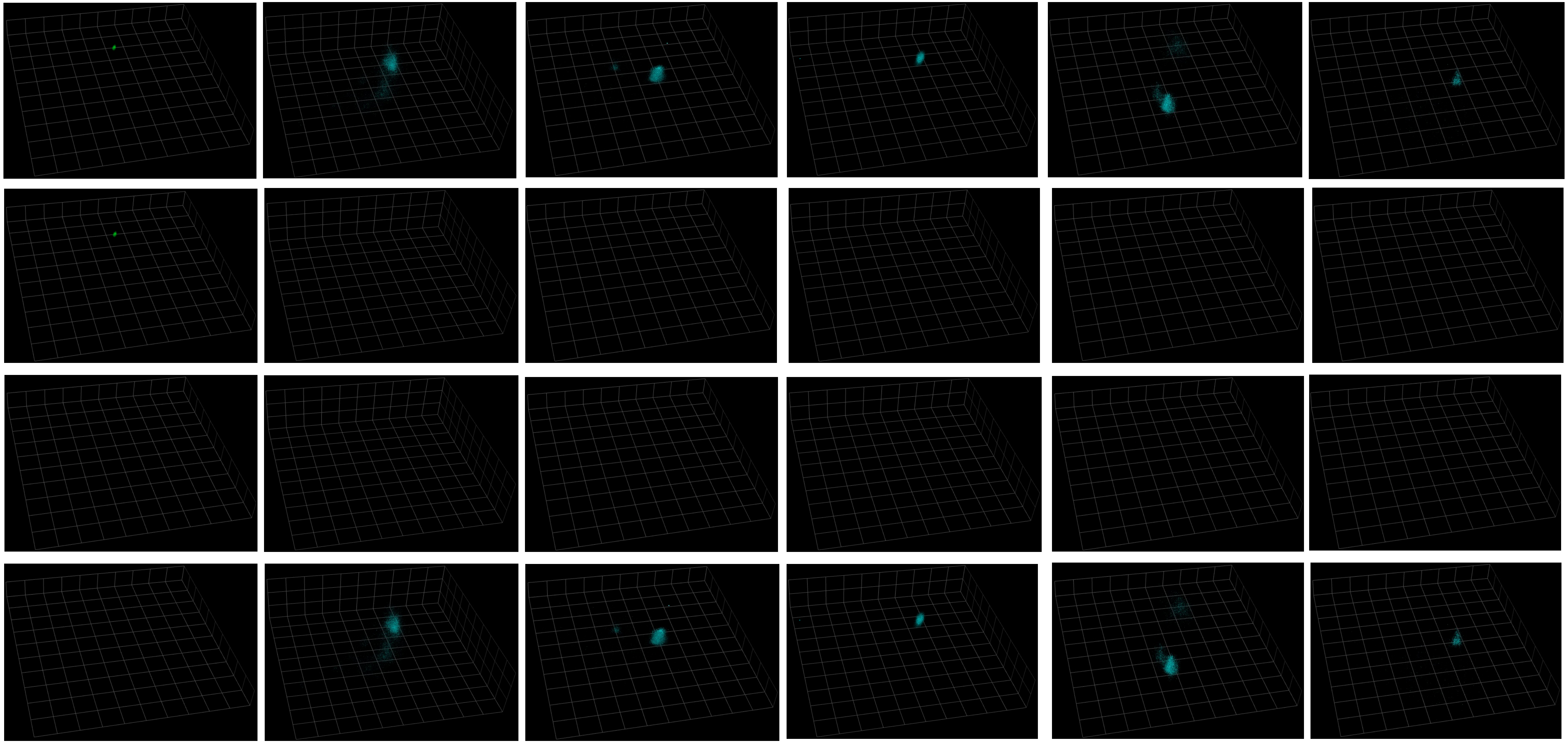

Combined Channels

PsearA (PA) probe

Psl0096 (anti-Psl antibody)

DAPI

1 Unit = 24.82 µm

NC007 Slice #2 (Negative PA Control)

20X Objective

Z-stack A | Z-stack B | Z-stack C | Z-stack D | Z-stack E | Z-stack F

Combined Channels

PsearA (PA) probe

Psl0096 (anti-Psl antibody)

DAPI

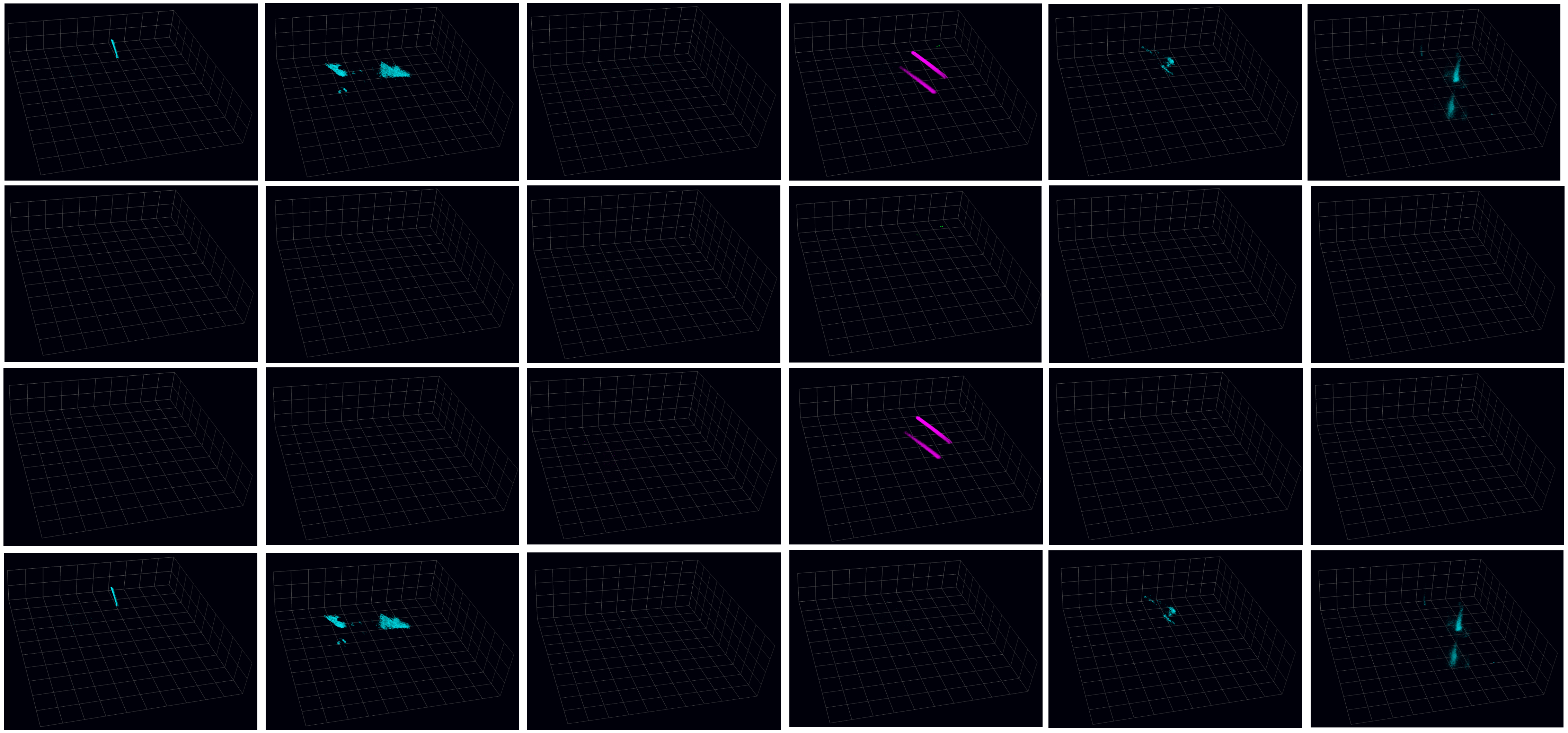

1 Unit = 24.82 µm

NC007 Slice #3 (Negative PA Control)

20X Objective

Z-stack A | Z-stack B | Z-stack C | Z-stack D | Z-stack E | Z-stack F

Combined Channels

PsearA (PA) probe

Psl0096 (anti-Psl antibody)

DAPI

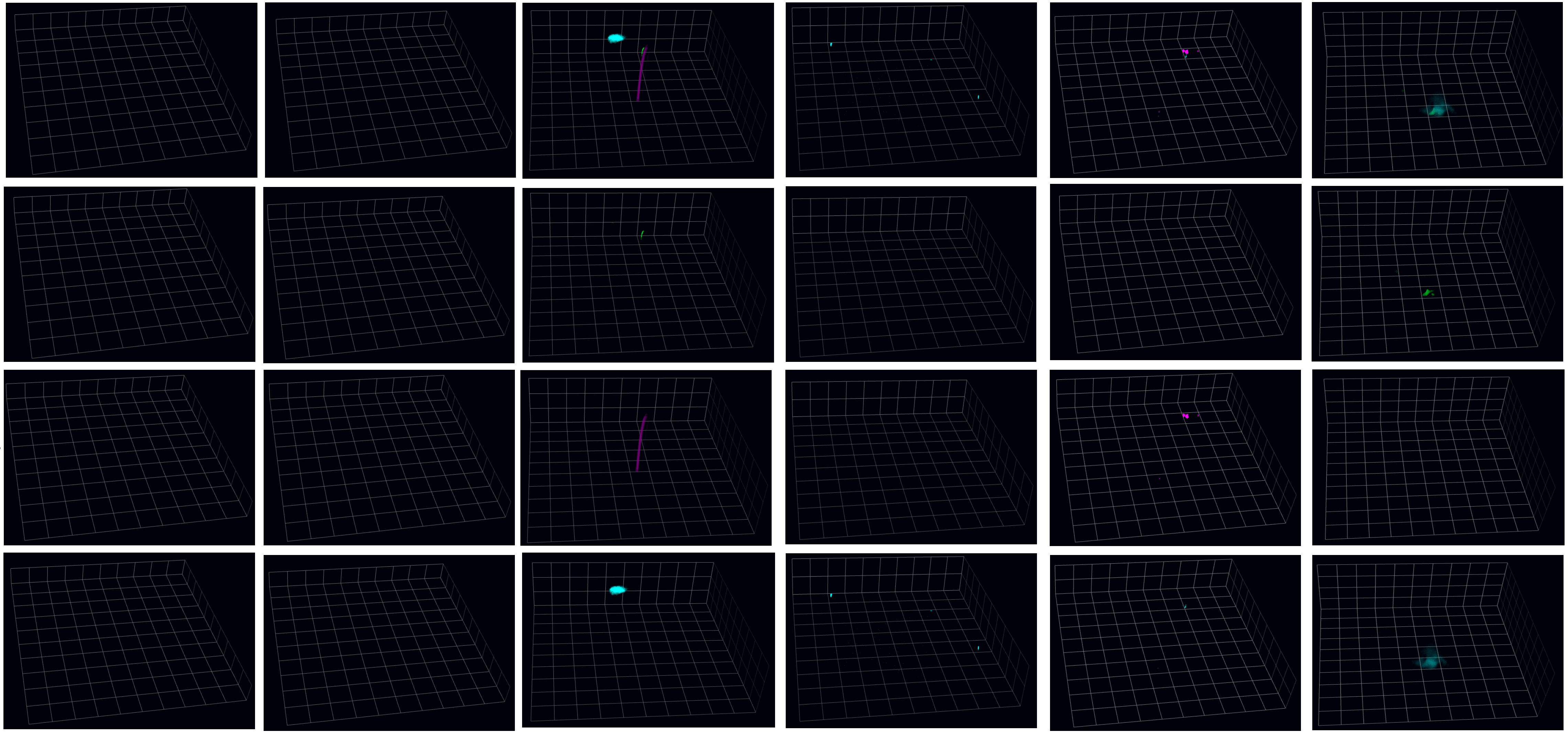

1 Unit = 24.82 μm

NC008 Slice #1 (Negative PA Control)

20X Objective

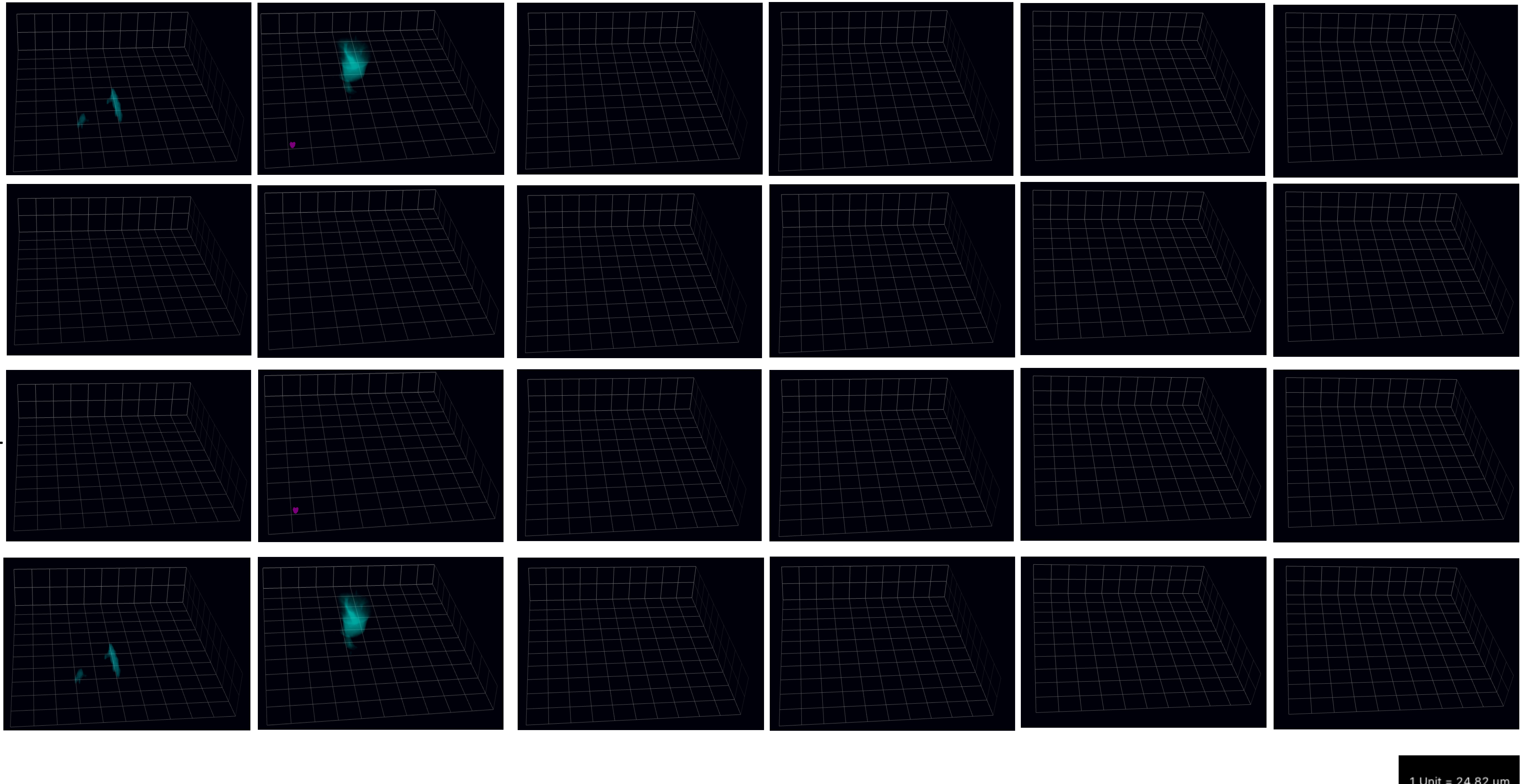

NC008 Slice #2 (Negative PA Control)

20X Objective

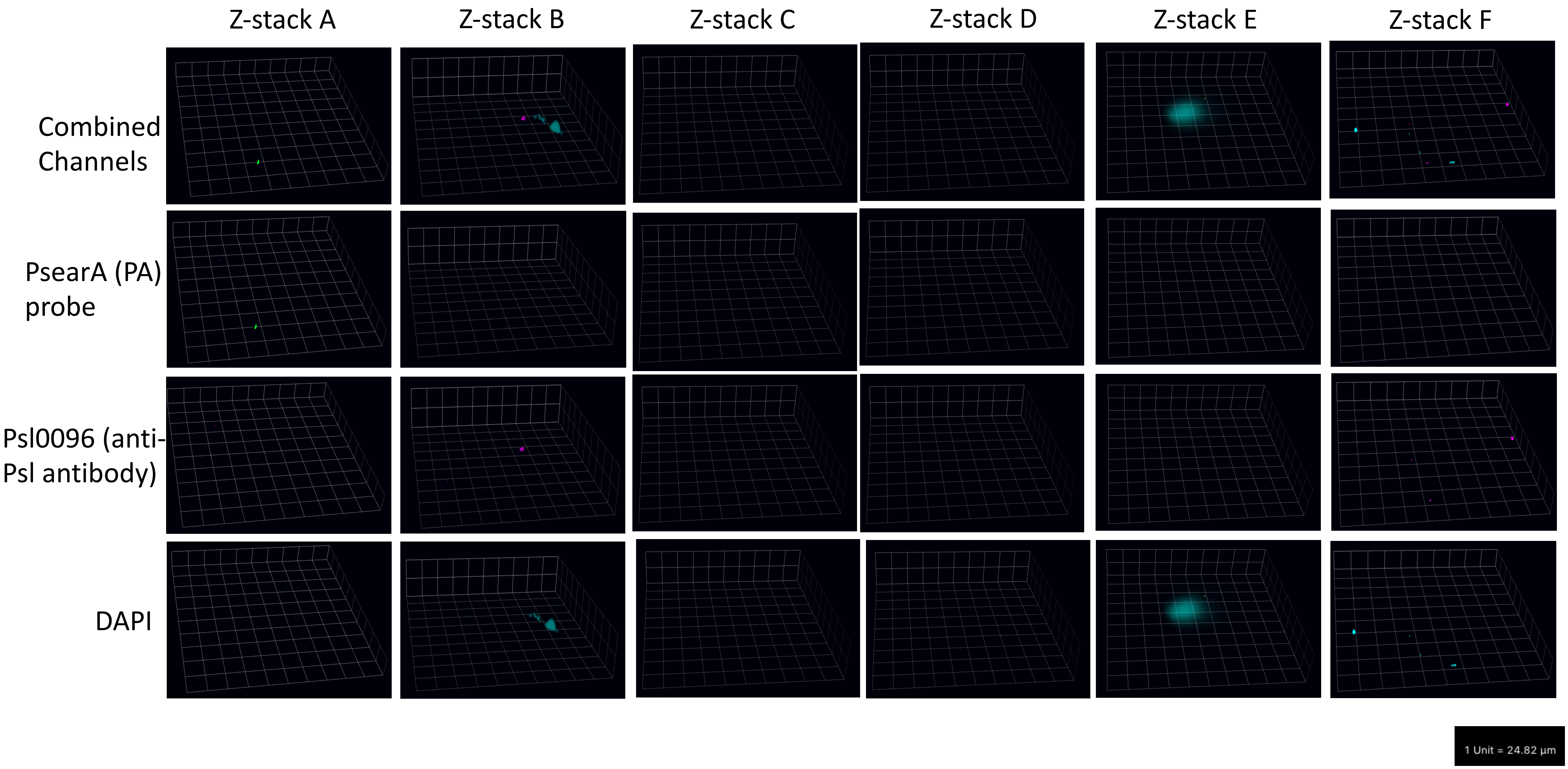

NC008 Slice #3 (Negative PA Control) 20X Objective

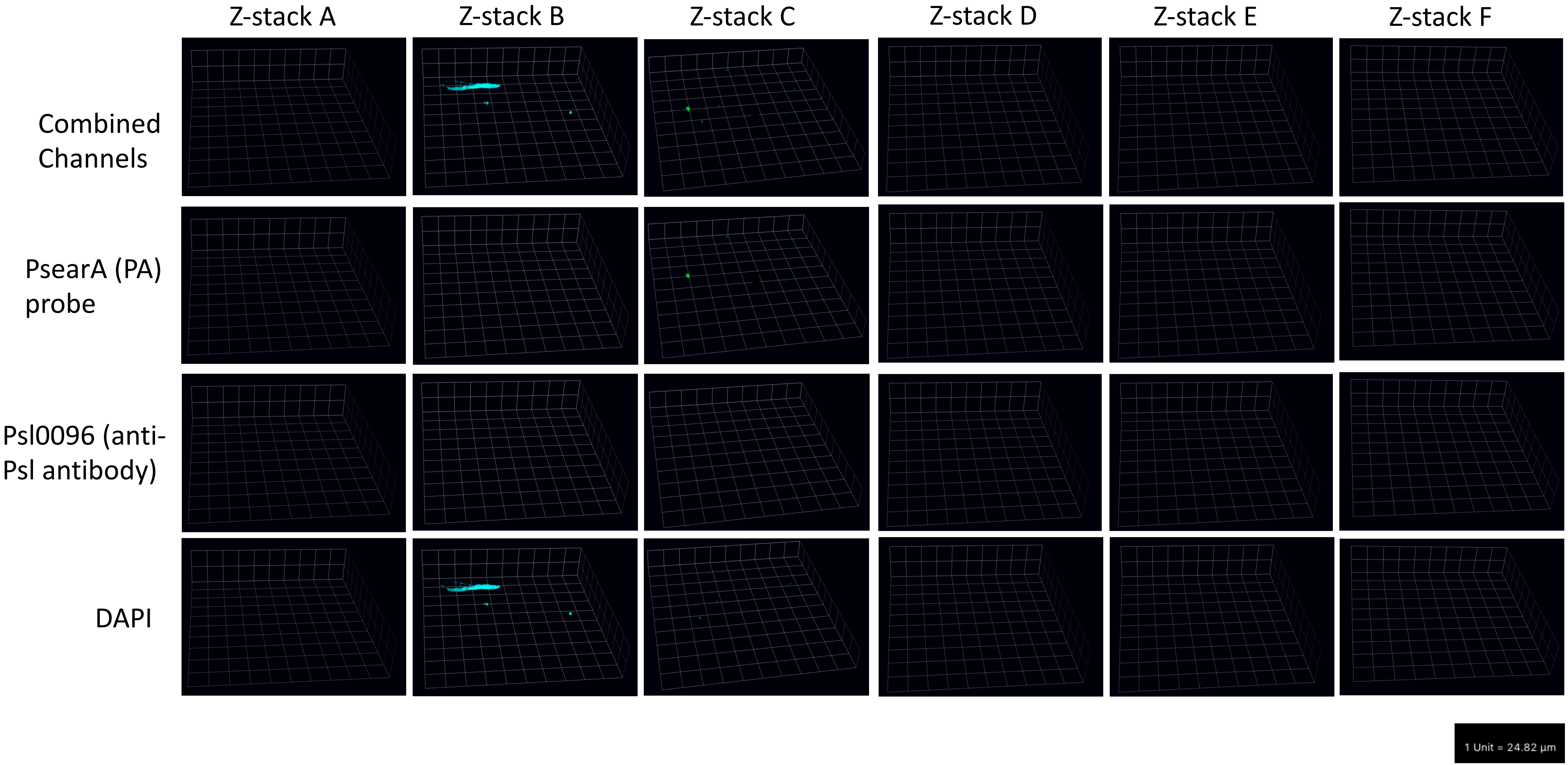

NC009 Slice #1 (Negative PA Control)

20X Objective

Z-stack A | Z-stack B | Z-stack C | Z-stack D | Z-stack E | Z-stack F

Rows: Combined Channels; PsearA (PA) probe; Psl0096 (anti-Psl antibody); DAPI

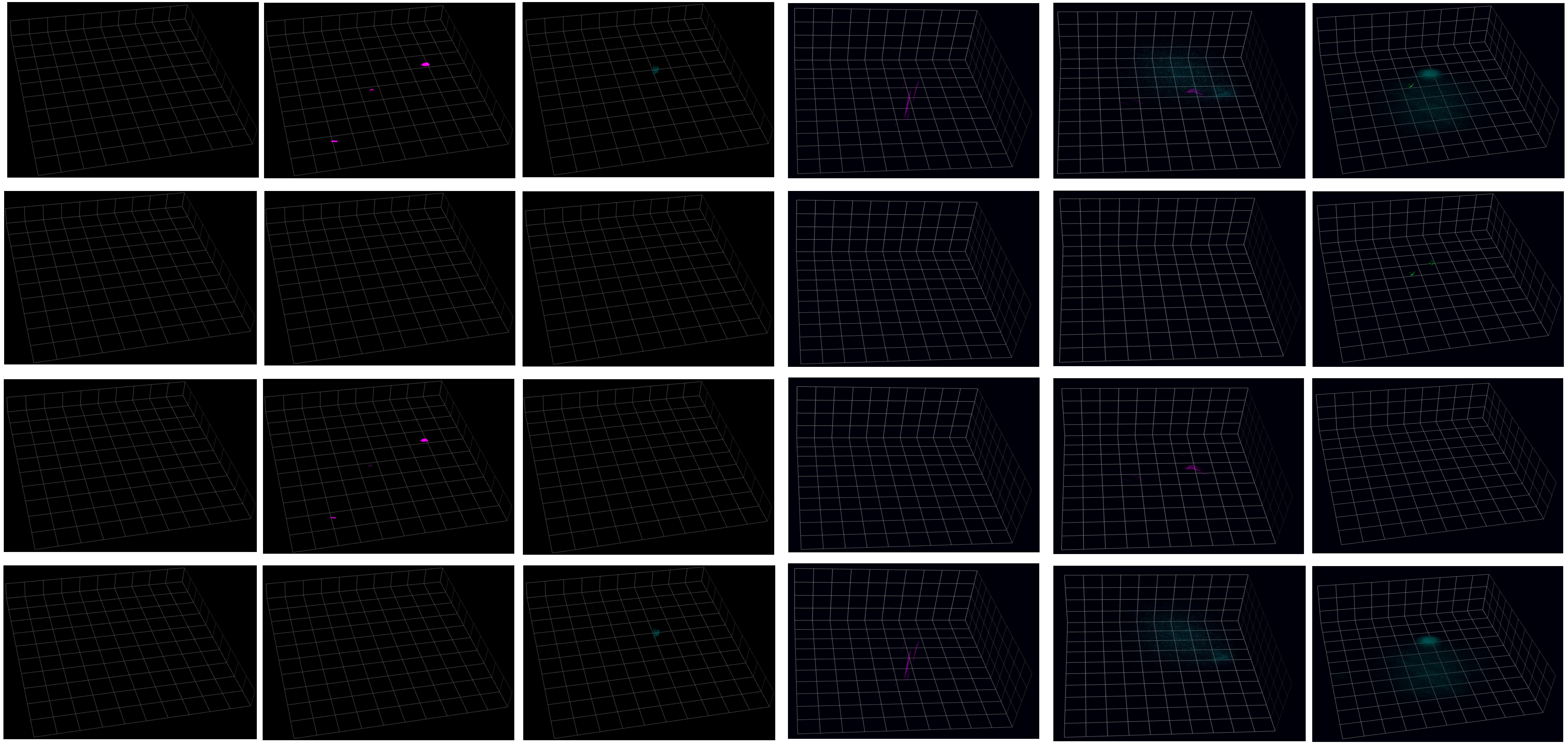

1 Unit = 24.82 µm

NC009 Slice #2 (Negative PA Control)

20X Objective

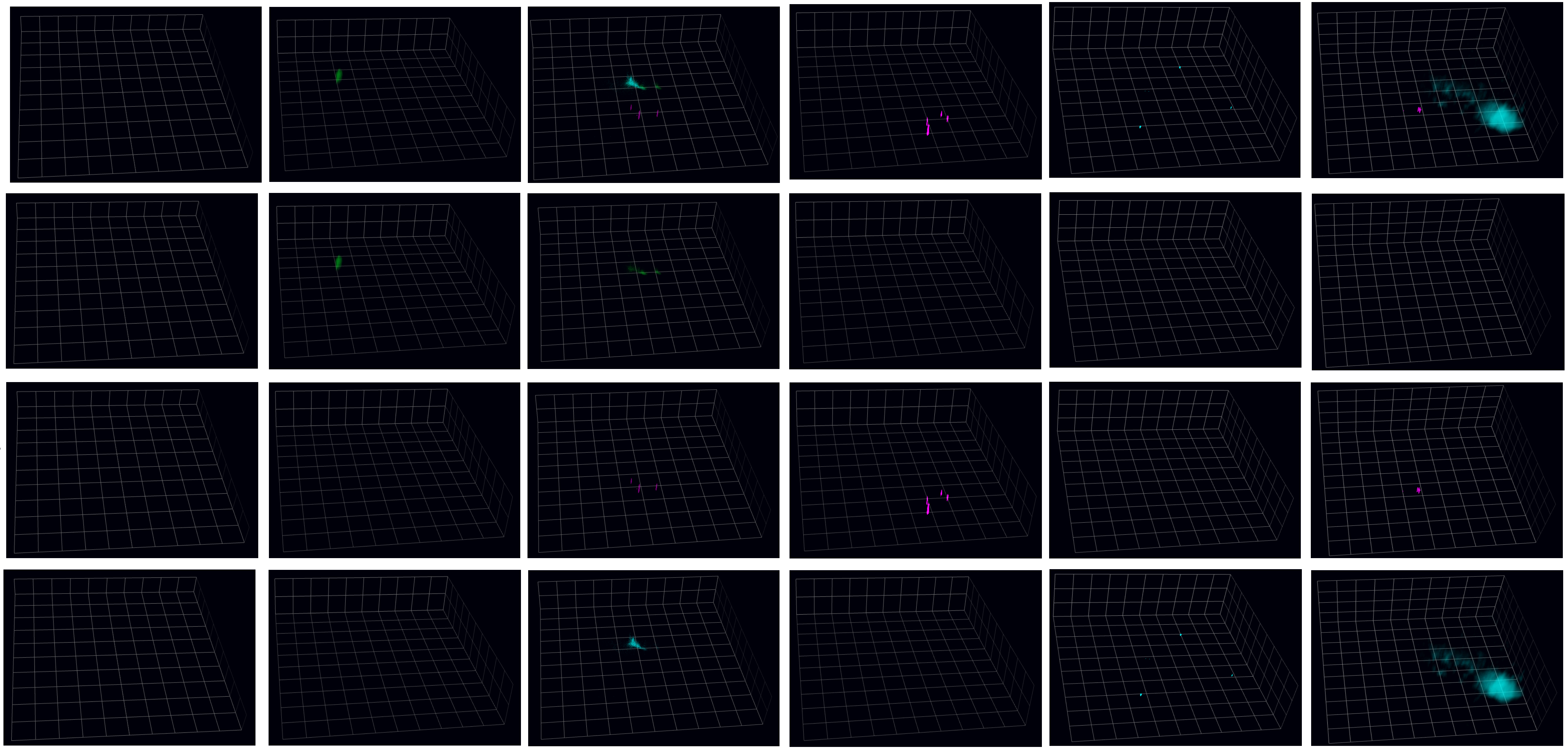

1 Unit = 24.82 µm

NC009 Slice #3 (Negative PA Control)

20X Objective

Z-stack A | Z-stack B | Z-stack C | Z-stack D | Z-stack E | Z-stack F

Combined Channels

PsearA (PA) probe

Psl0096 (anti-Psl antibody)

DAPI

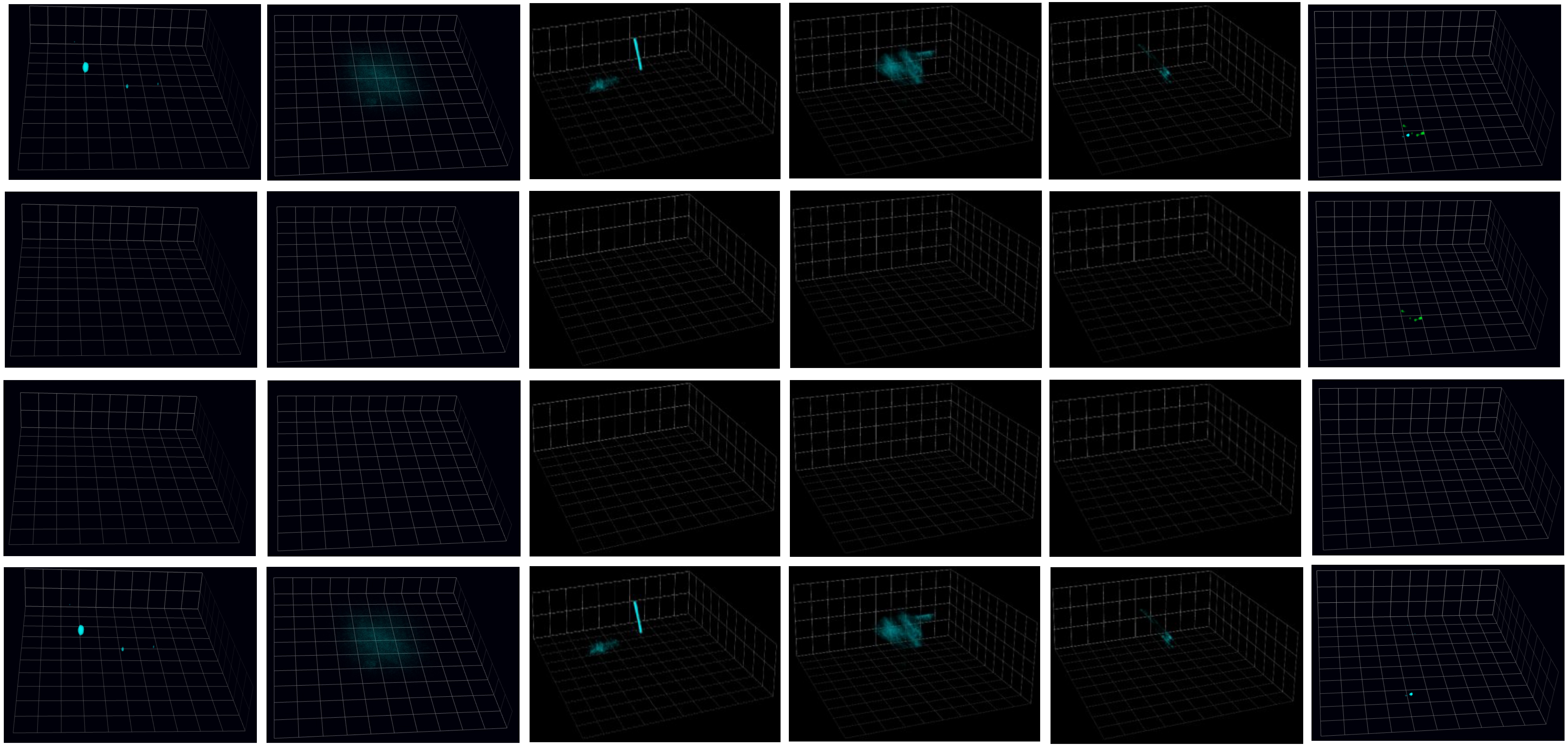

1 Unit = 24.82 µm

PAE001 Slice #1 (Persistent) 20X Objective

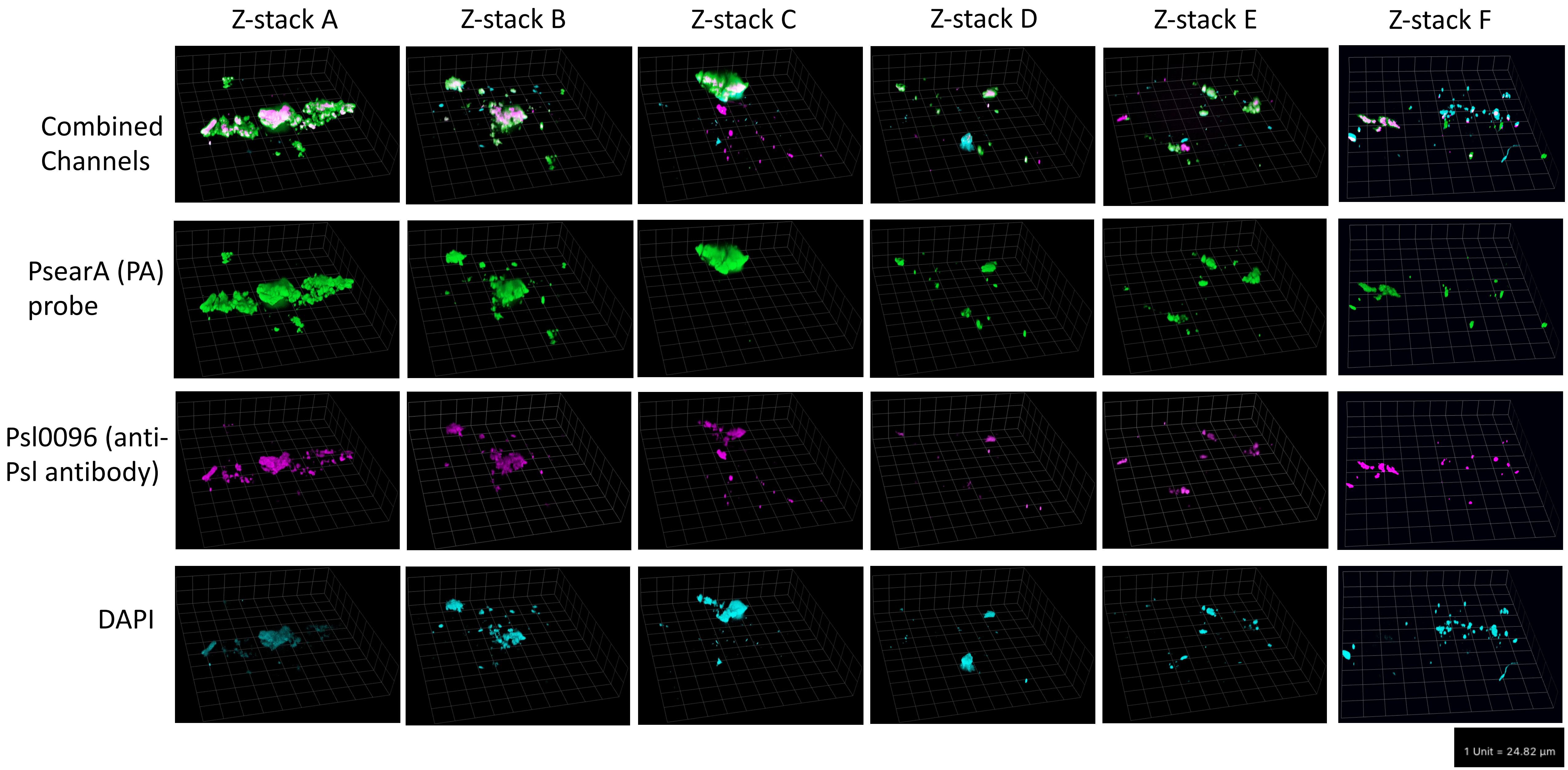

PAE001 Slice #2 (Persistent) 20X Objective

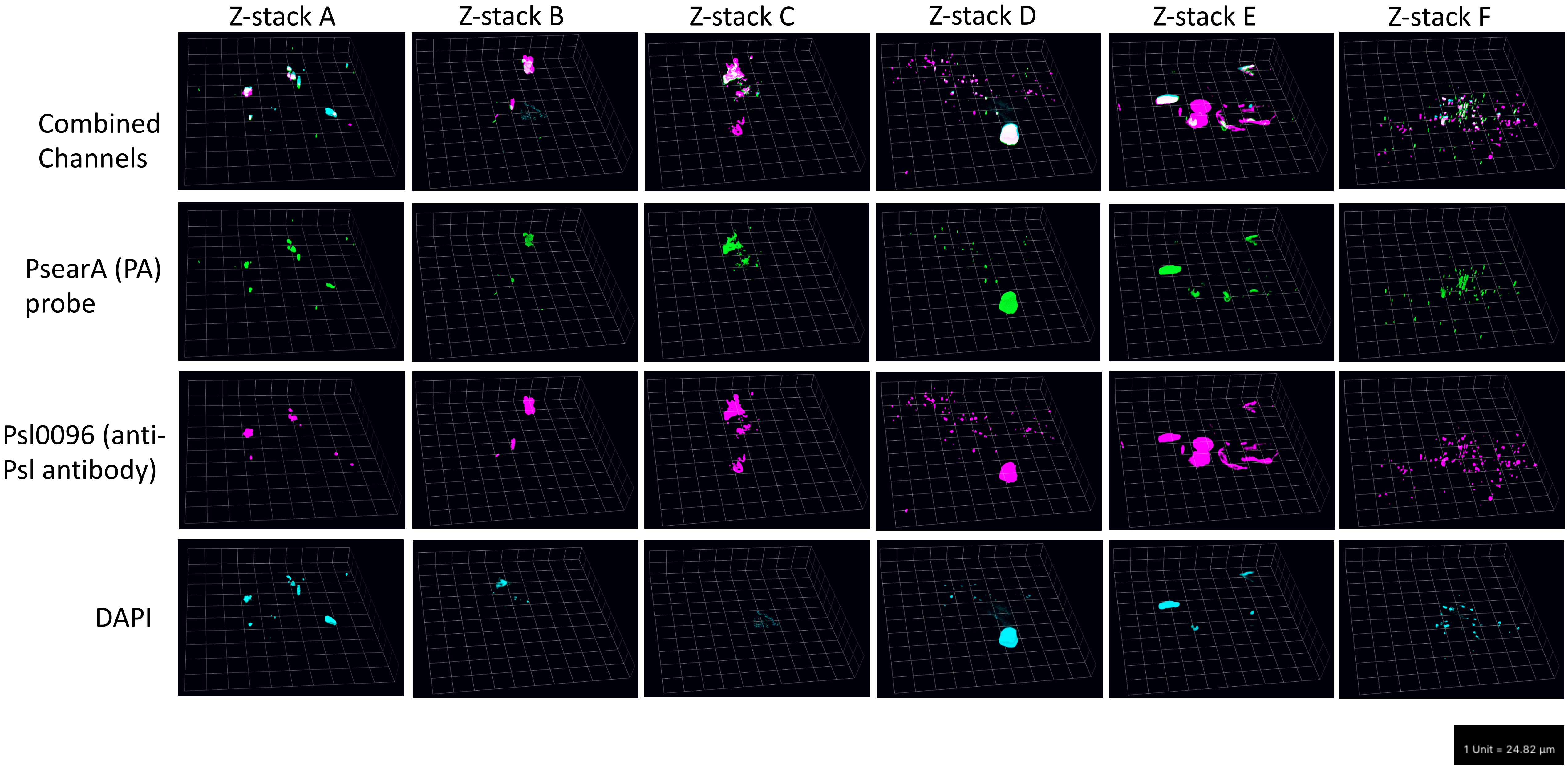

PAE001 Slice #3 (Persistent)

20X Objective

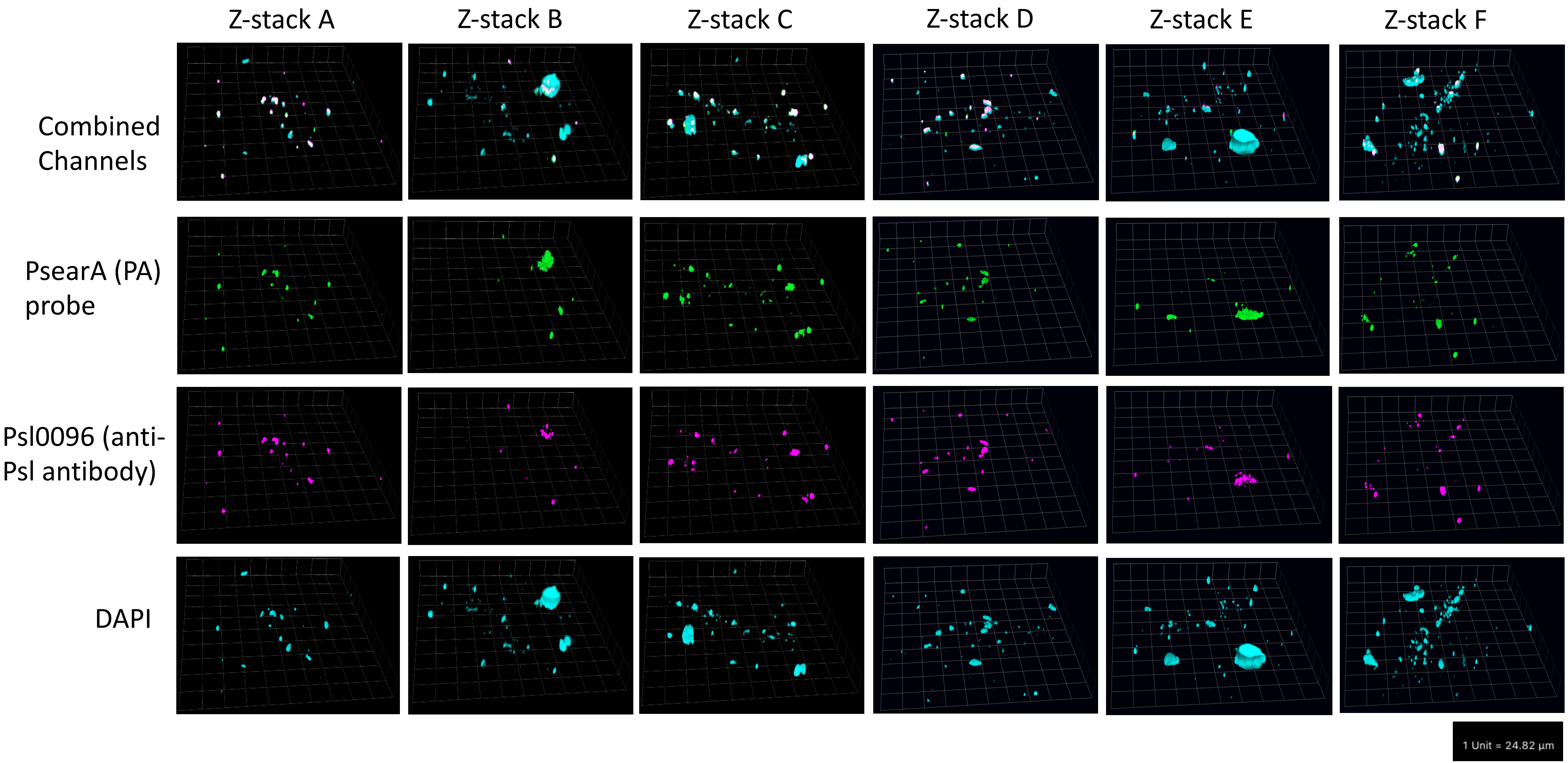

PAE005 Slice #1 (Persistent)

20X Objective

Z-stack A | Z-stack B | Z-stack C | Z-stack D | Z-stack E | Z-stack F

Combined Channels

PsearA (PA) probe

Psl0096 (anti-Psl antibody)

DAPI

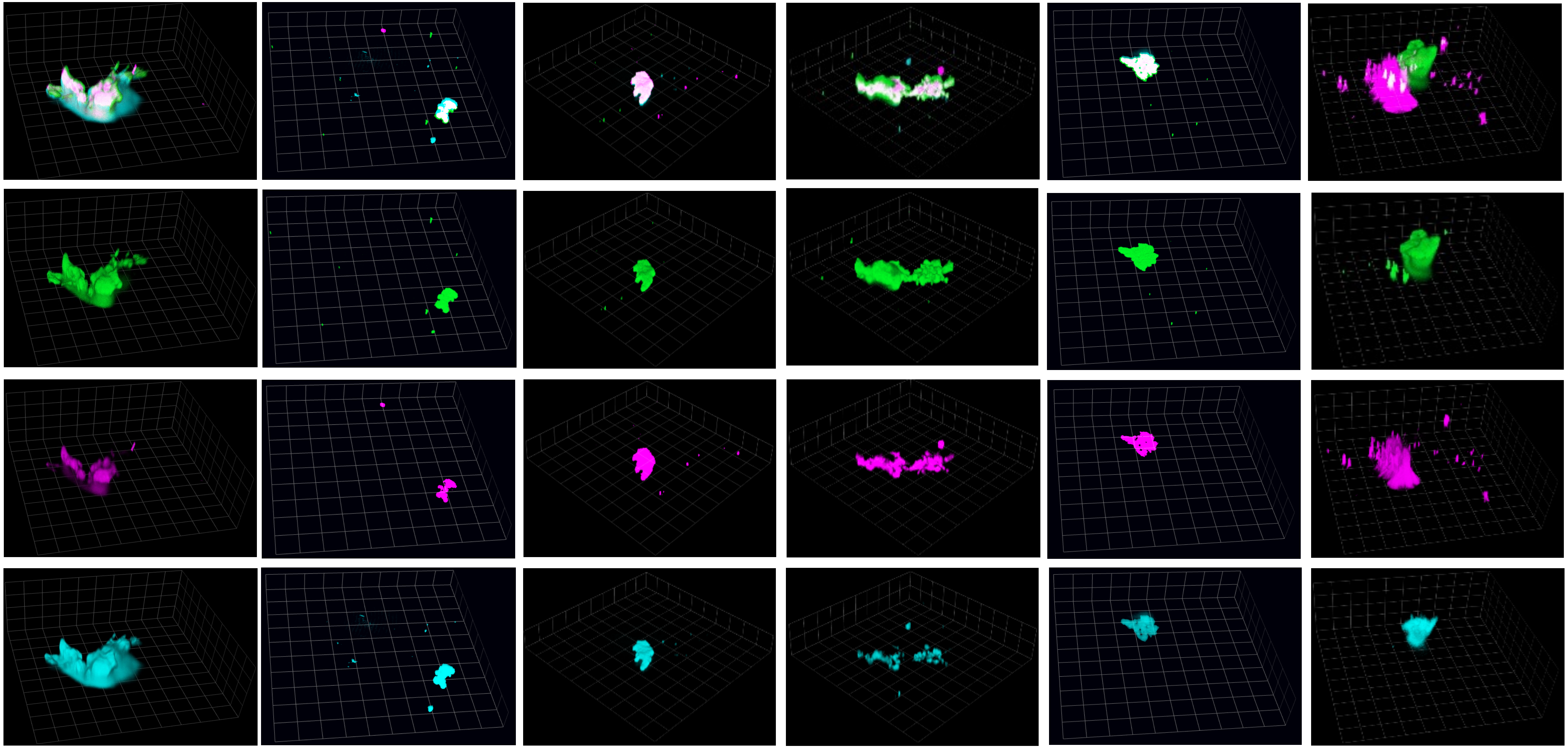

1 Unit = 24.82 µm

PAE005 Slice #2 (Persistent)

20X Objective

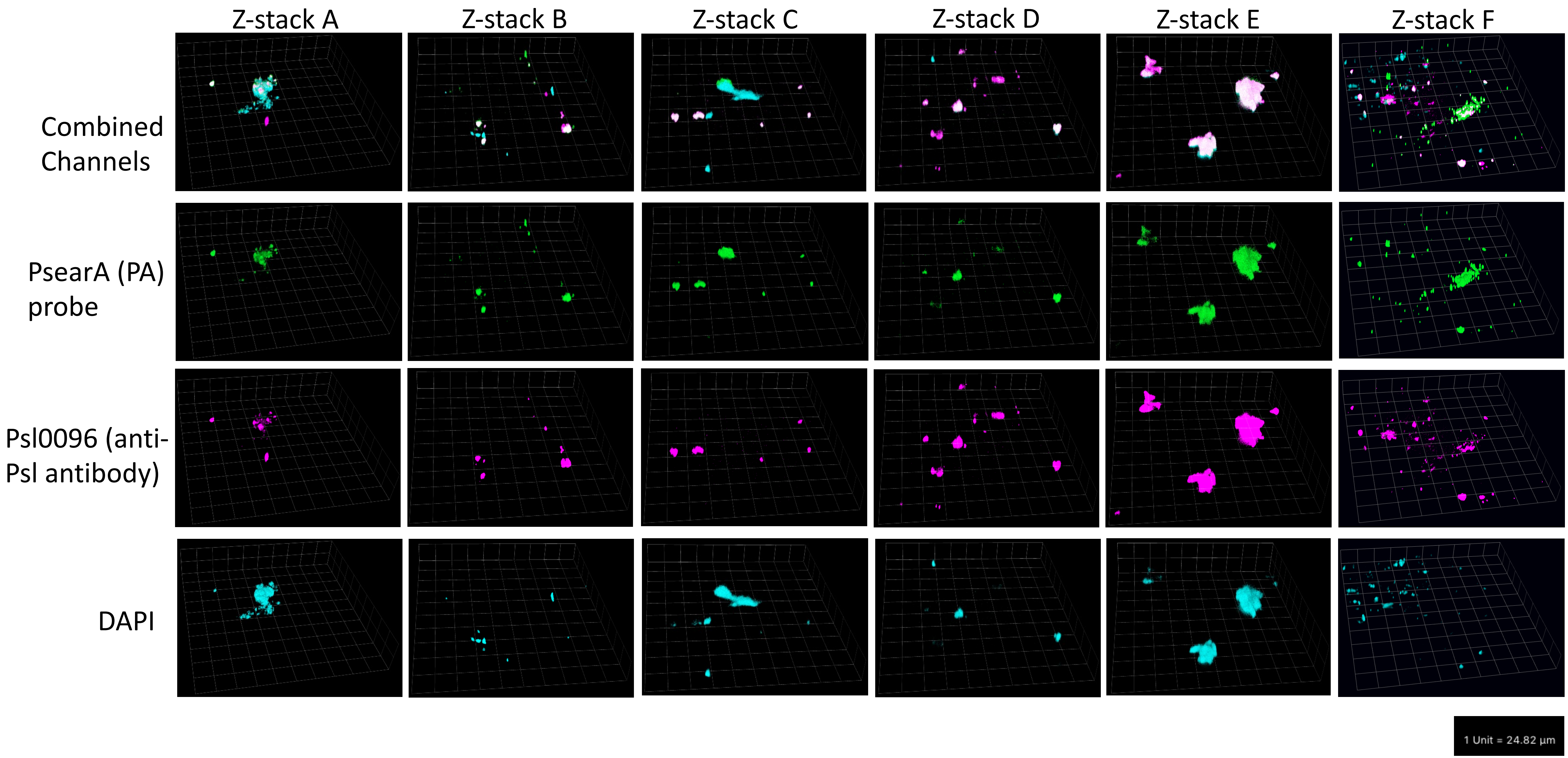

PAE005 Slice #3 (Persistent)

20X Objective

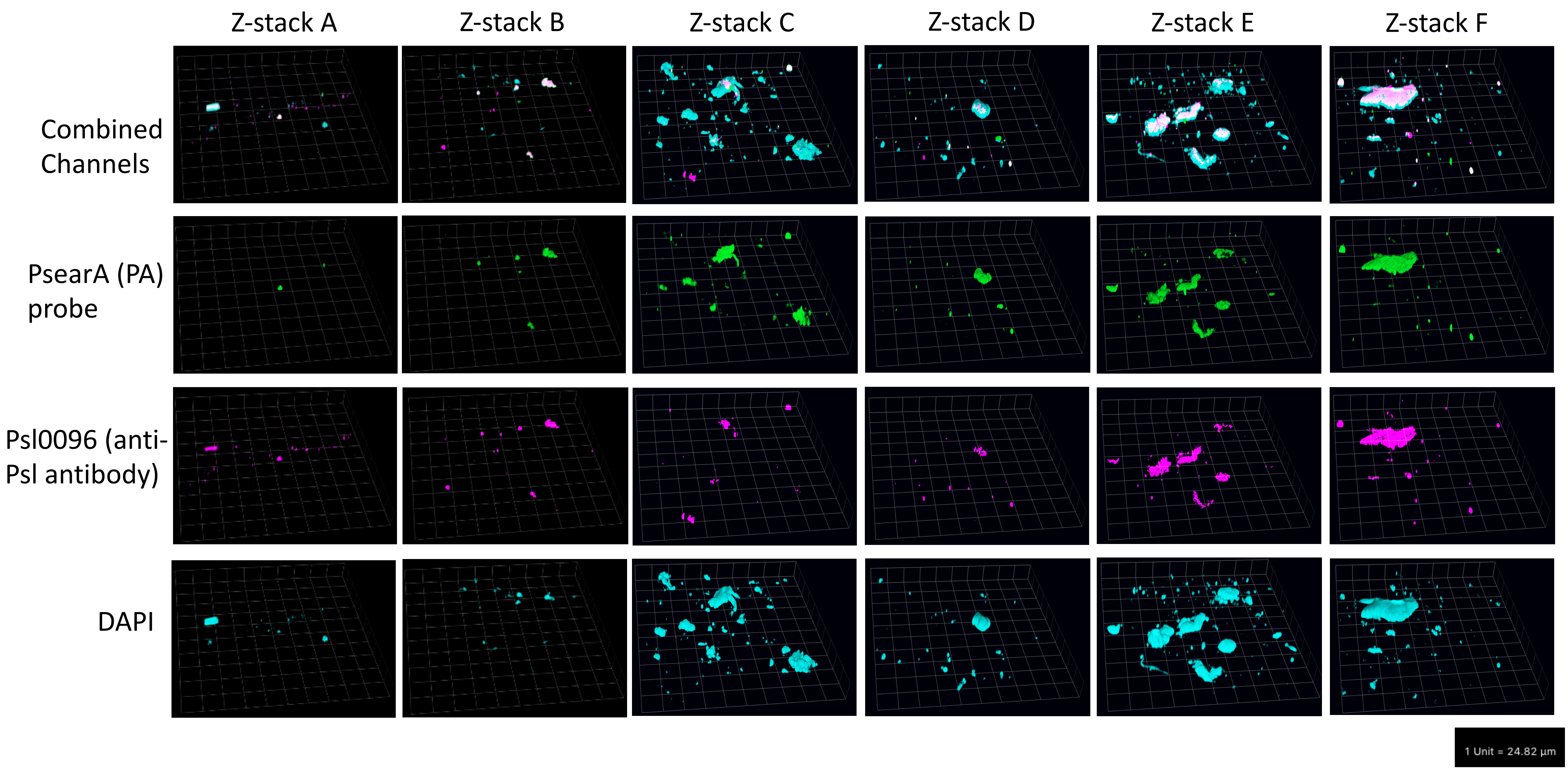

PAE008 Slice #1 (Persistent)

20X Objective

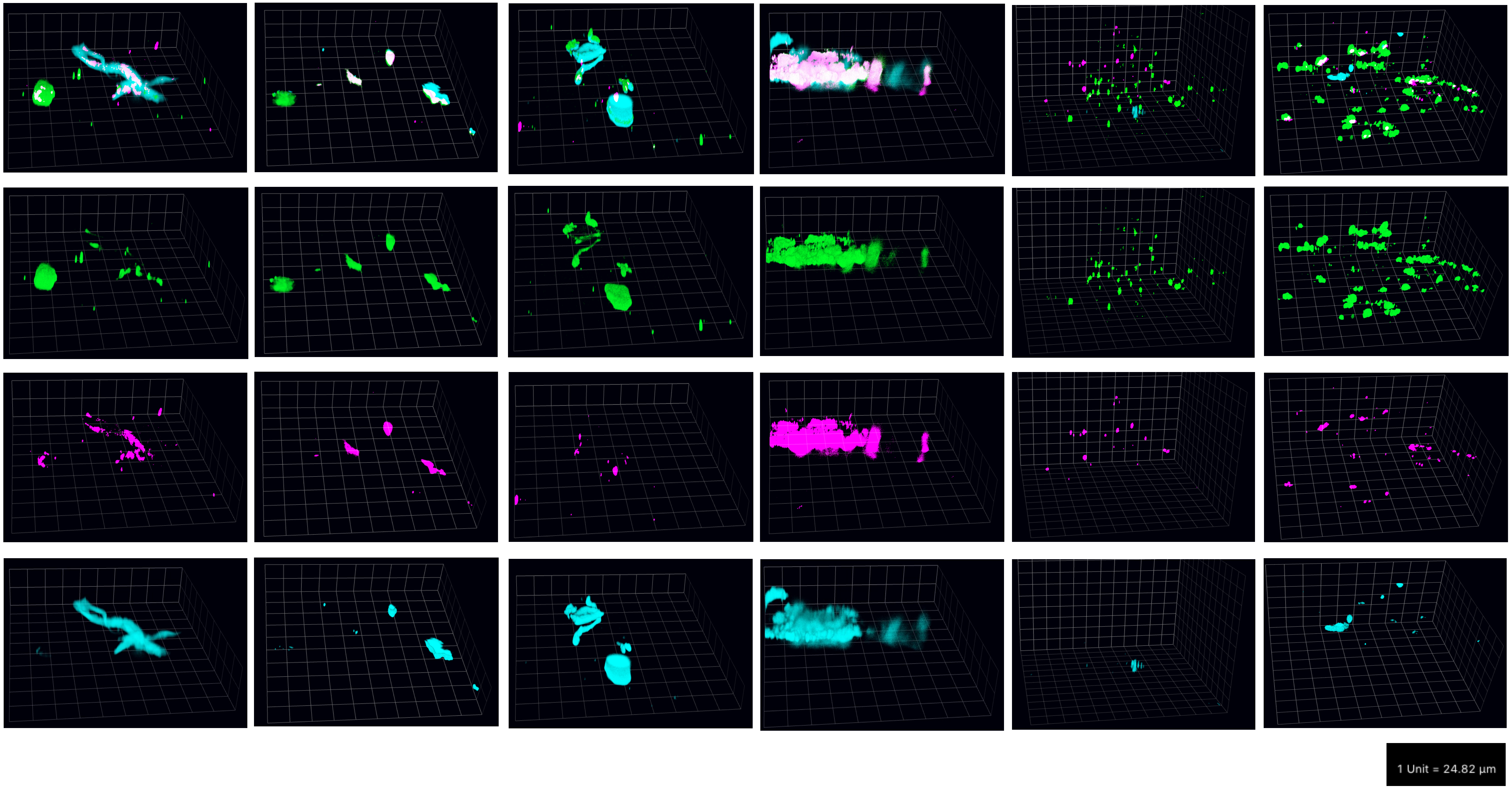

PAE008 Slice #2 (Persistent)

20X Objective

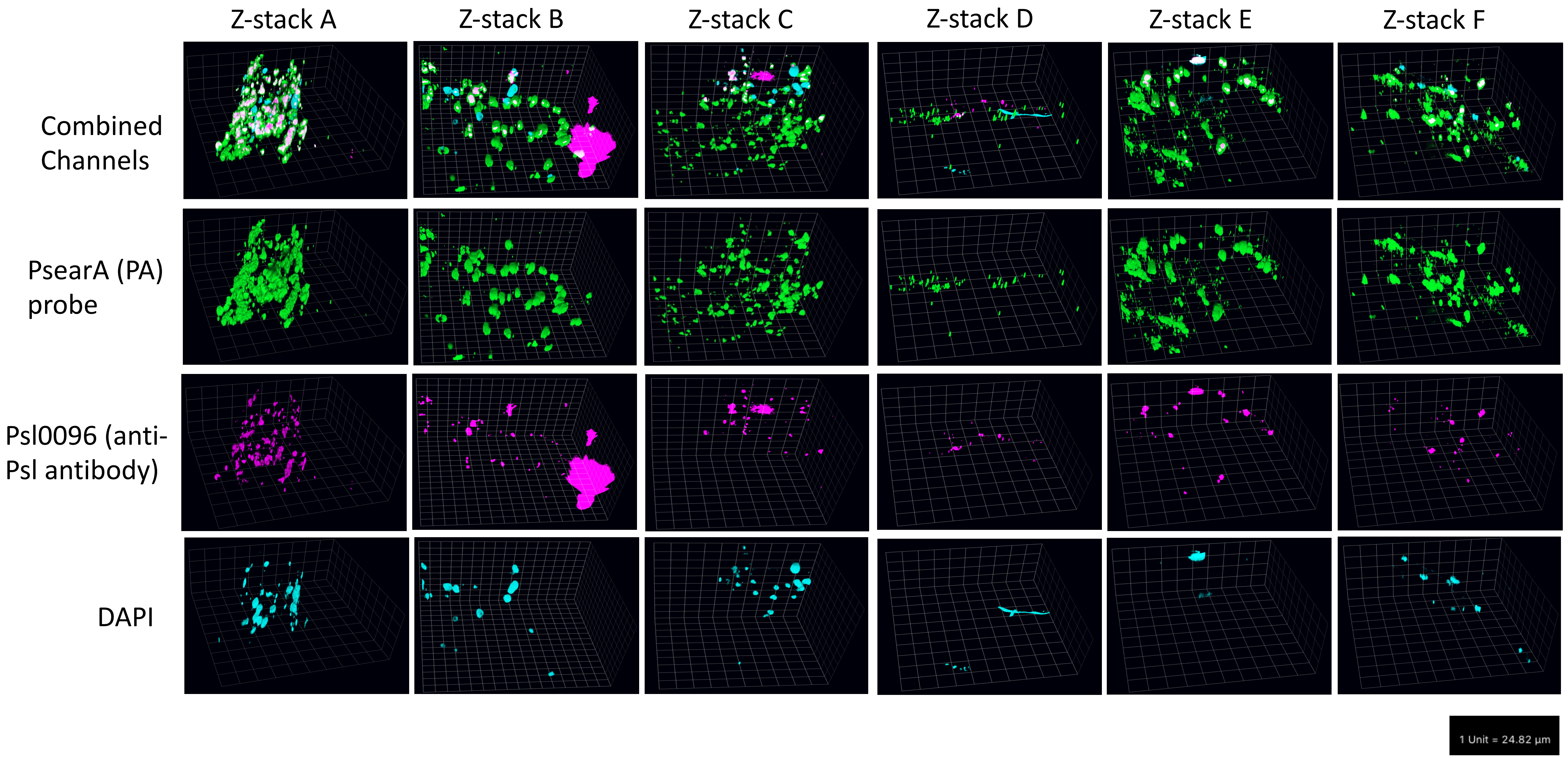

PAE008 Slice #3 (Persistent)

20X Objective

Z-stack A | Z-stack B | Z-stack C | Z-stack D | Z-stack E | Z-stack F

Combined Channels

PsearA (PA) probe

Psl0096 (anti-Psl antibody)

DAPI

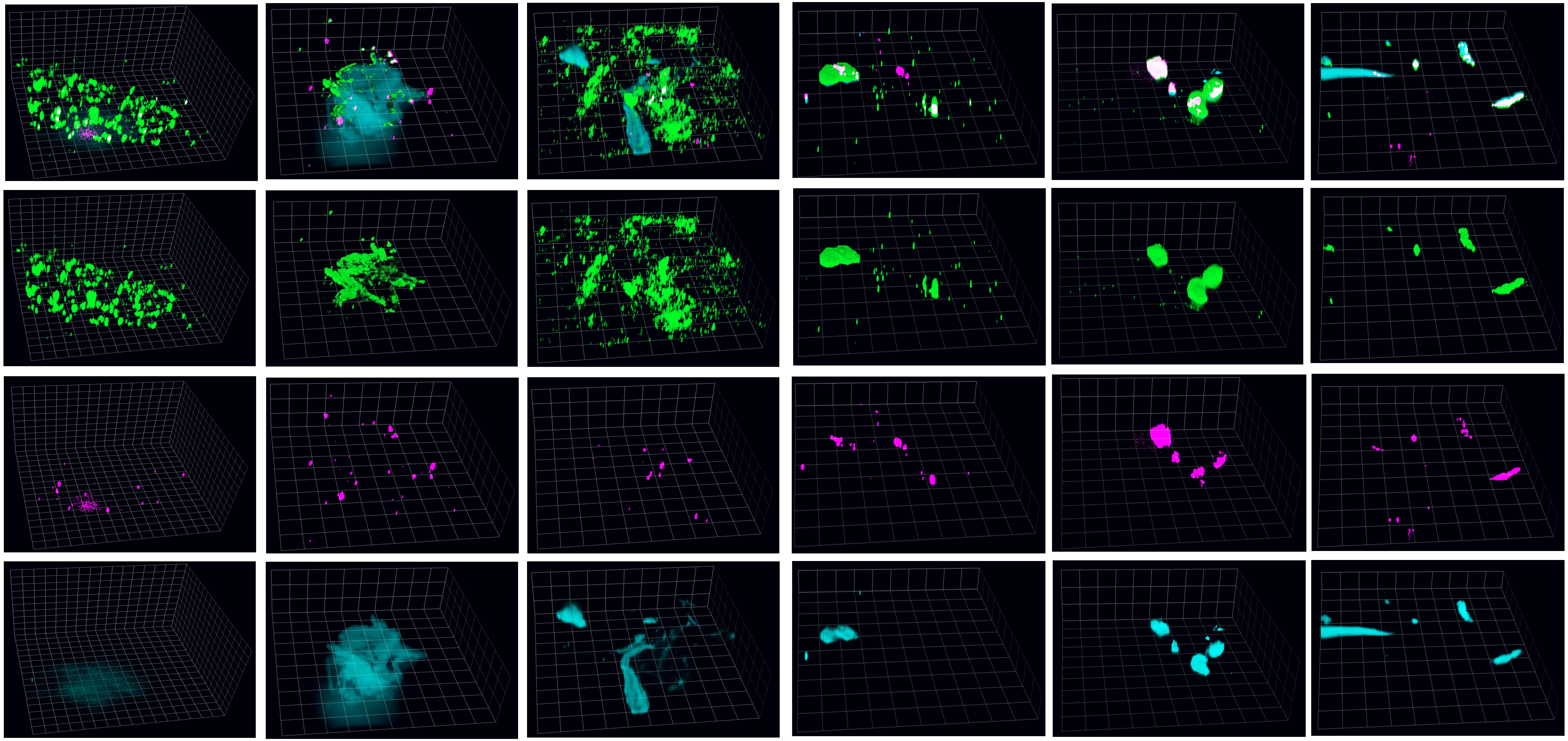

1 Unit = 24.82 µm

PAE011 Slice #1 (Persistent)

20X Objective

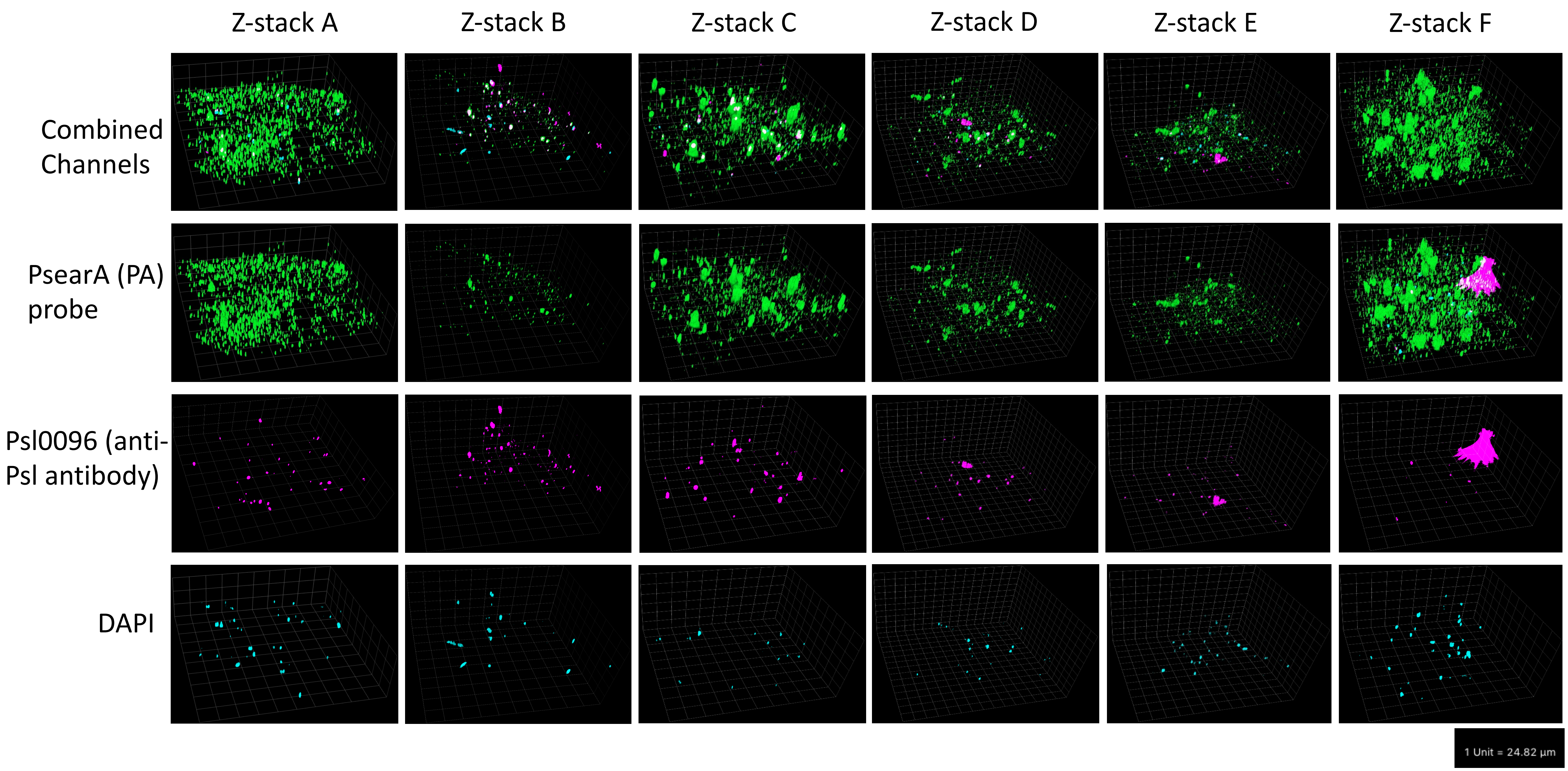

PAE011 Slice #2 (Persistent)

20X Objective

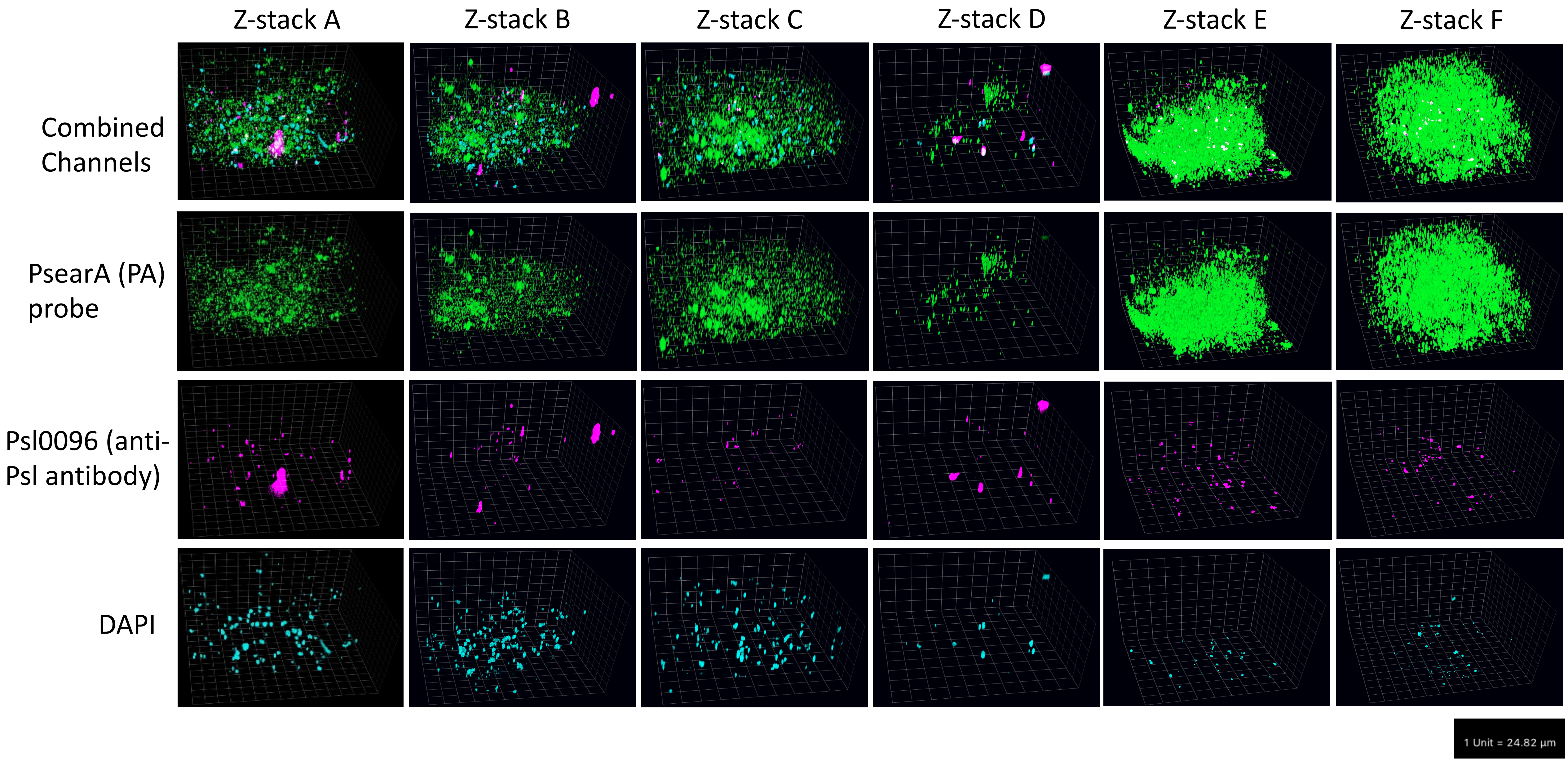

PAE011 Slice #3 (Persistent)

20X Objective

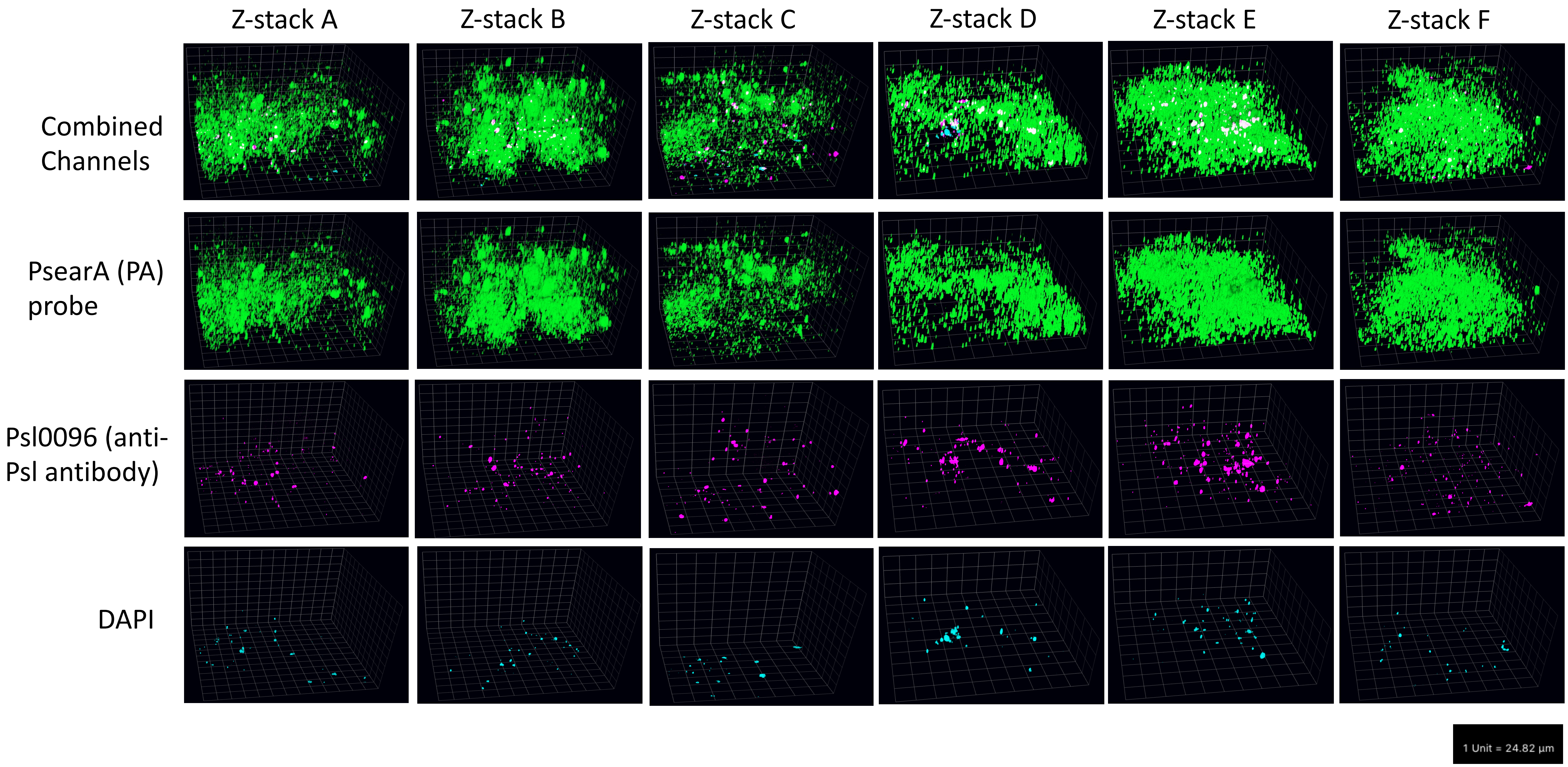

PAE002 Slice #1 (Eradicated) 20X Objective

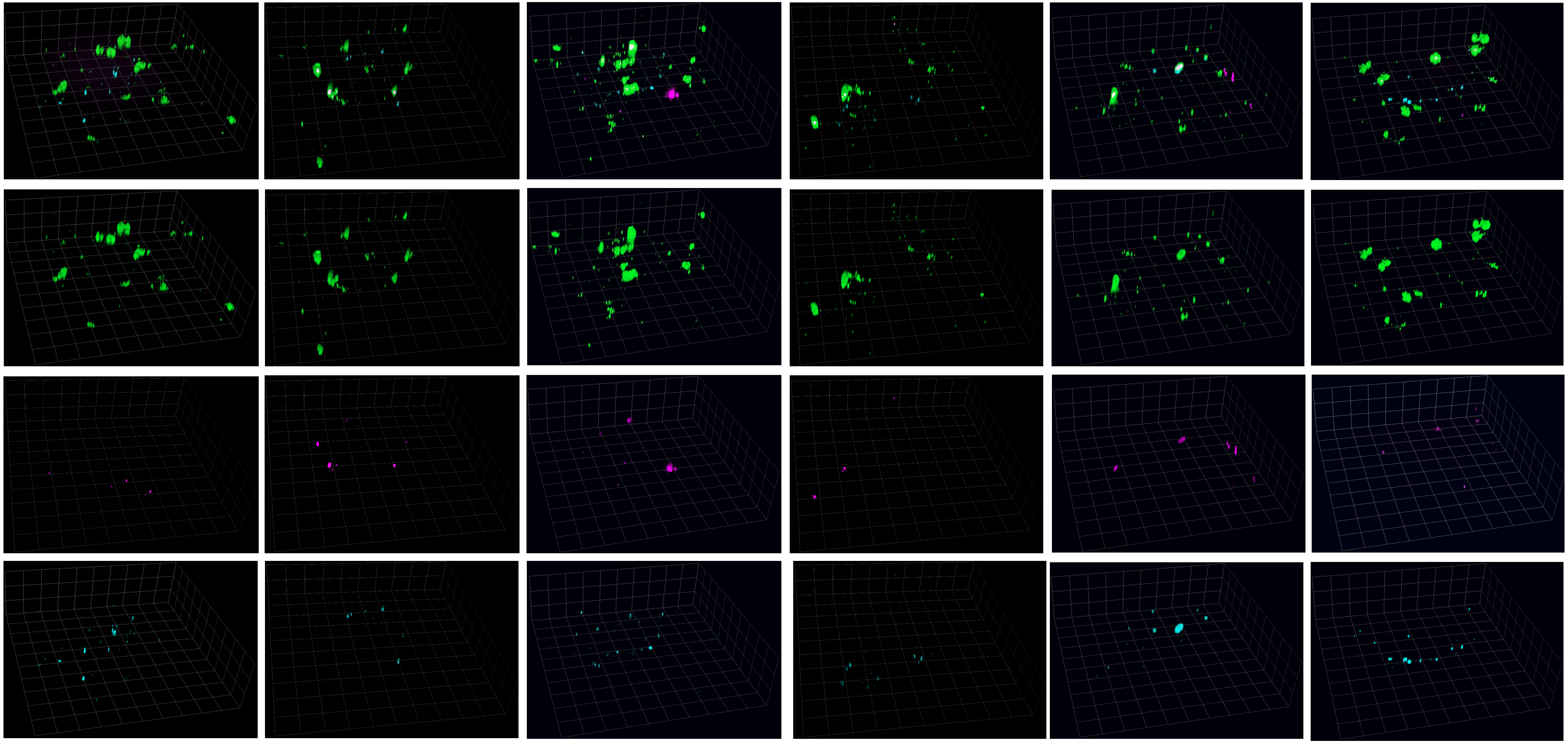

PAE002 Slice #2 (Eradicated)

20X Objective

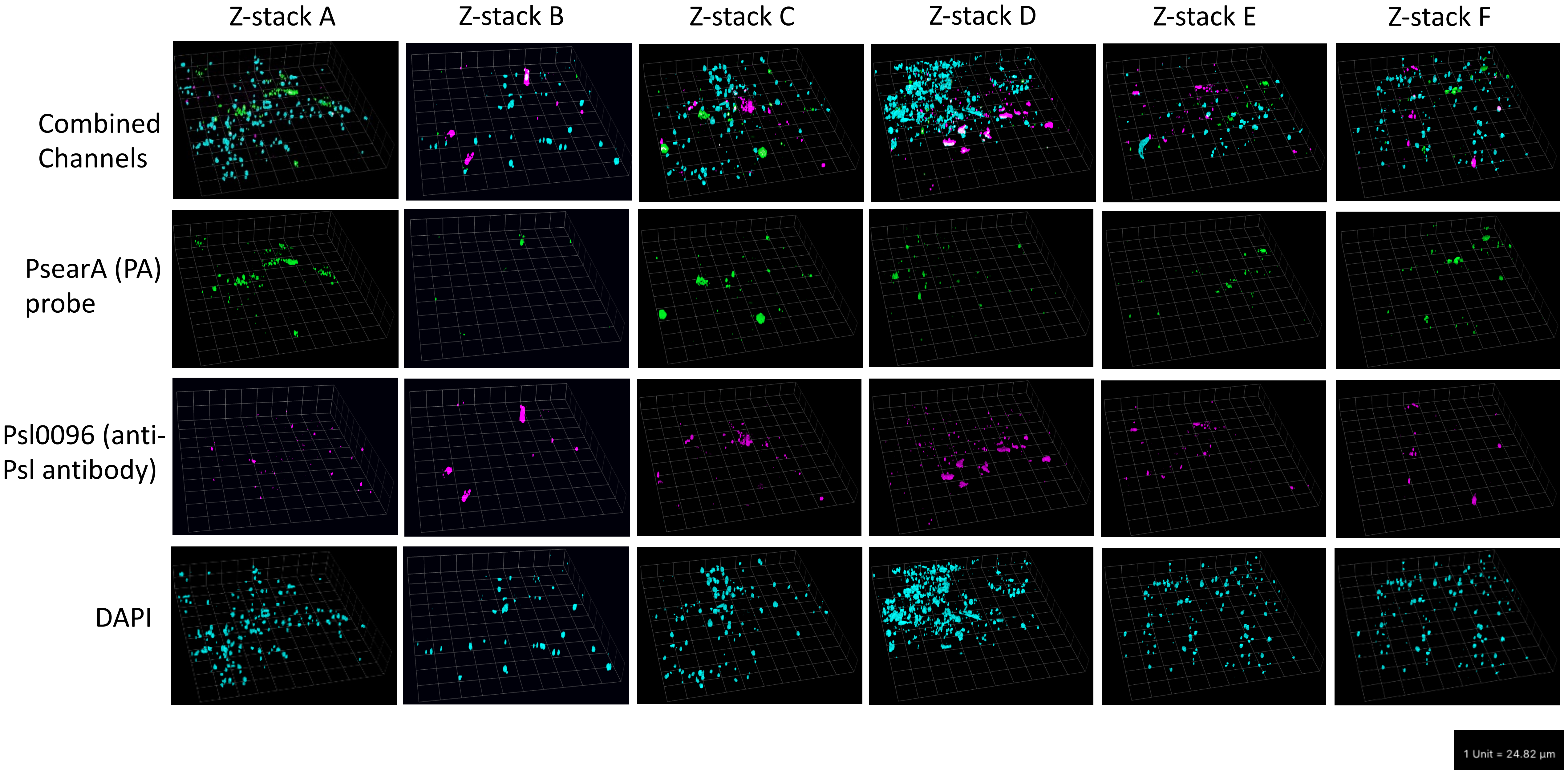

PAE002 Slice #3 (Eradicated)

20X Objective

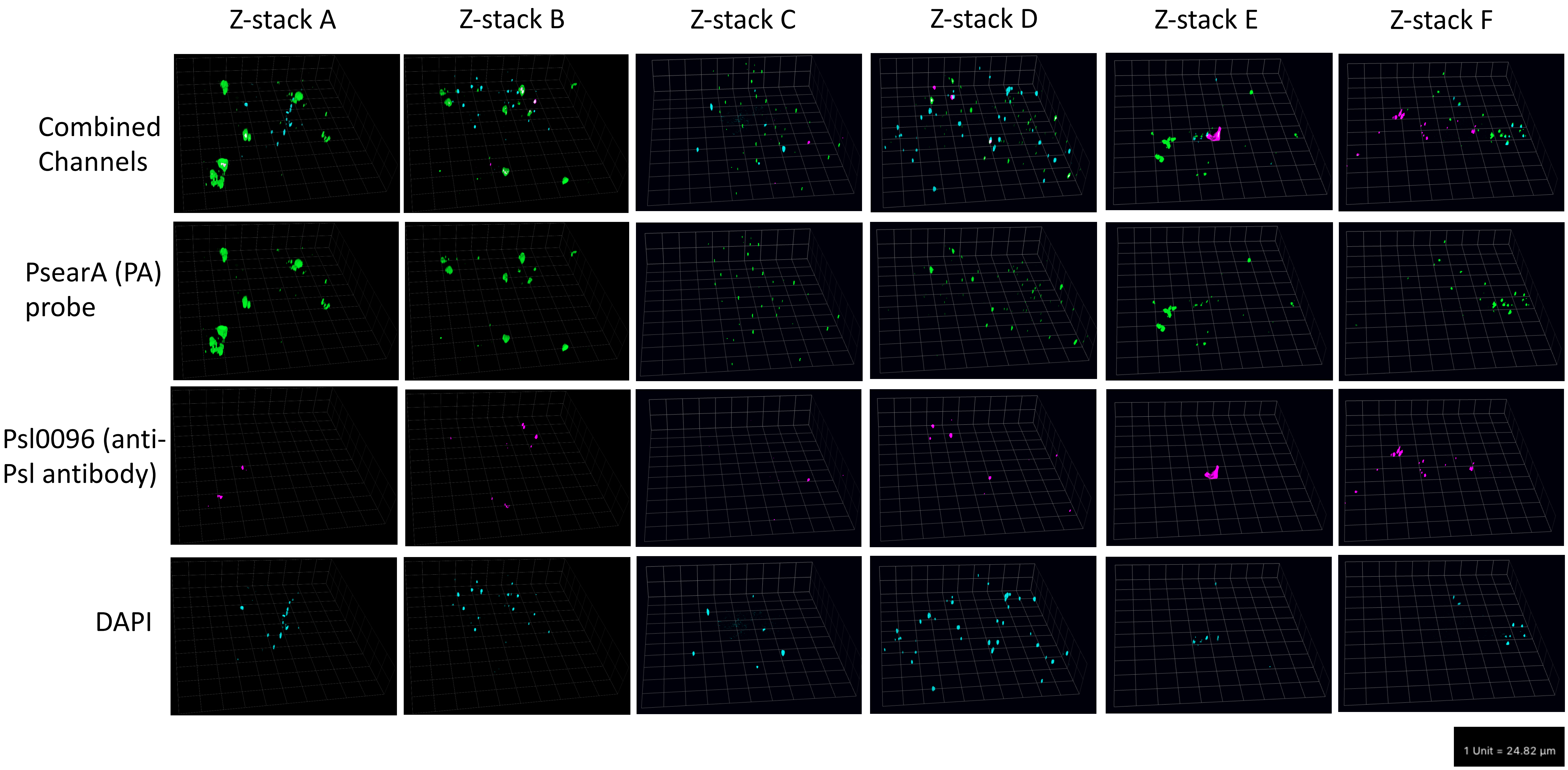

PAE003 Slice #1 (Eradicated)

20X Objective

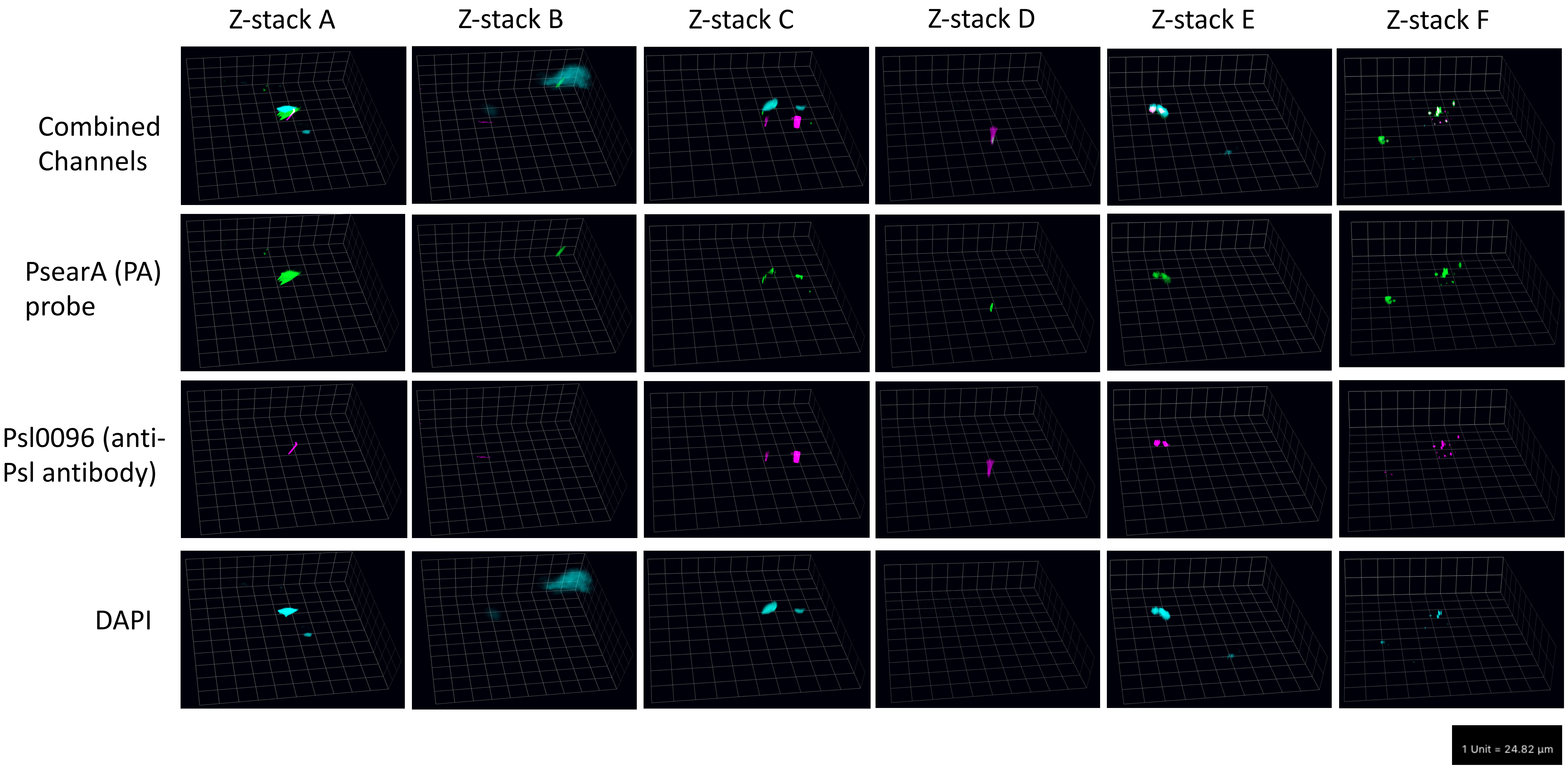

PAE003 Slice #2 (Eradicated)

20X Objective

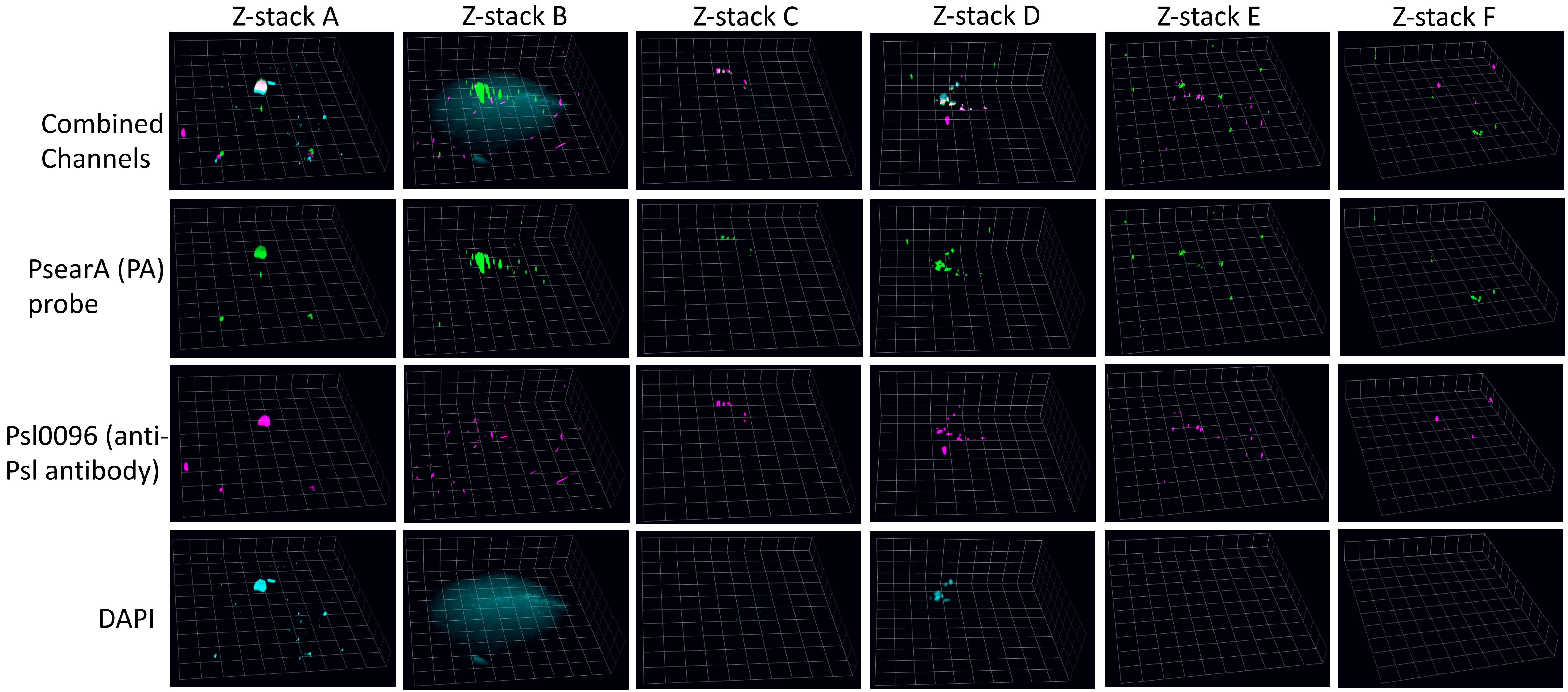

1 Unit = 24.82 µm

PAE003 Slice #3 (Eradicated)

20X Objective

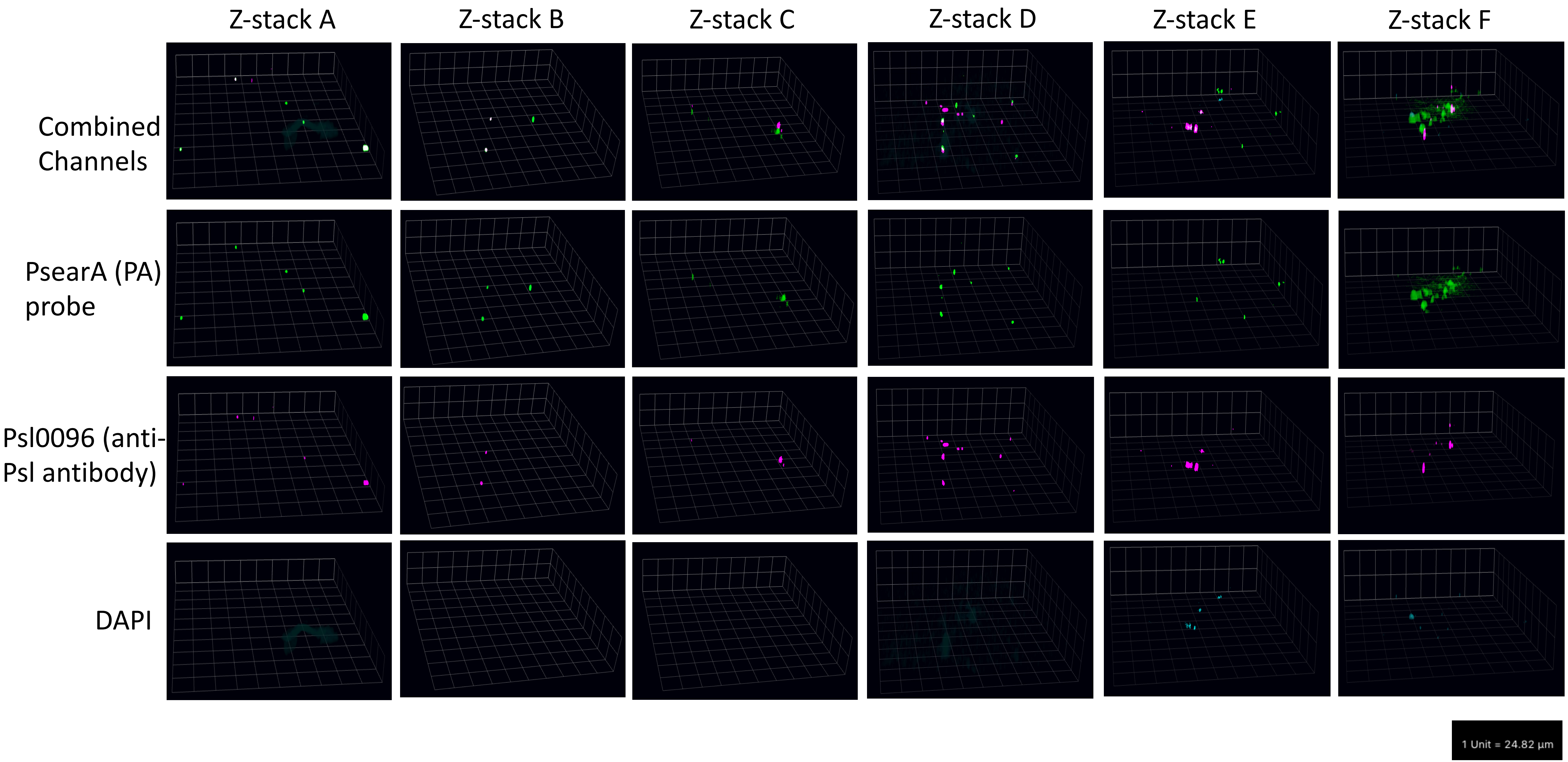

PAE004 Slice #1 (Eradicated)

20X Objective

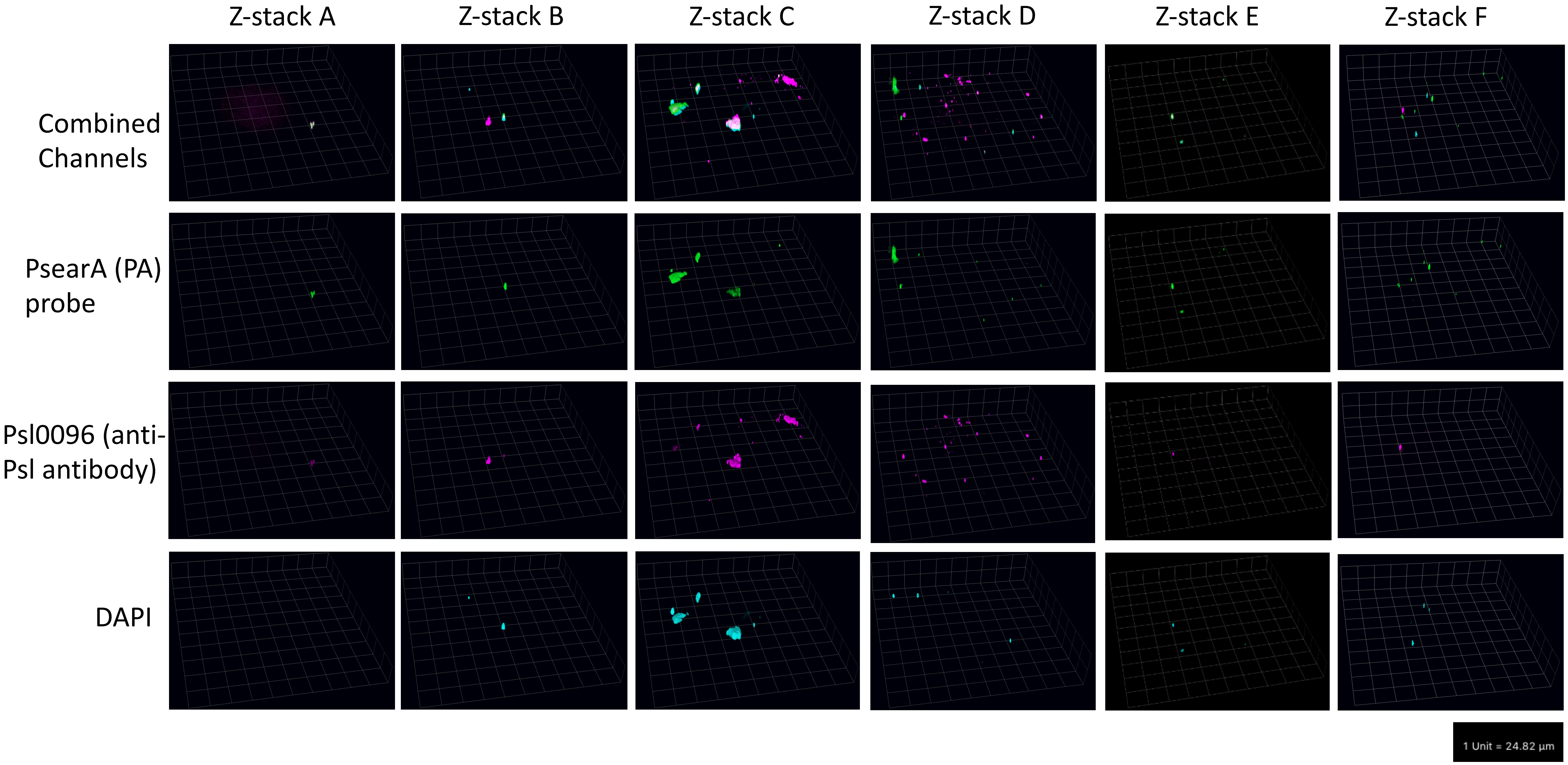

PAE004 Slice #2 (Eradicated)

20X Objective

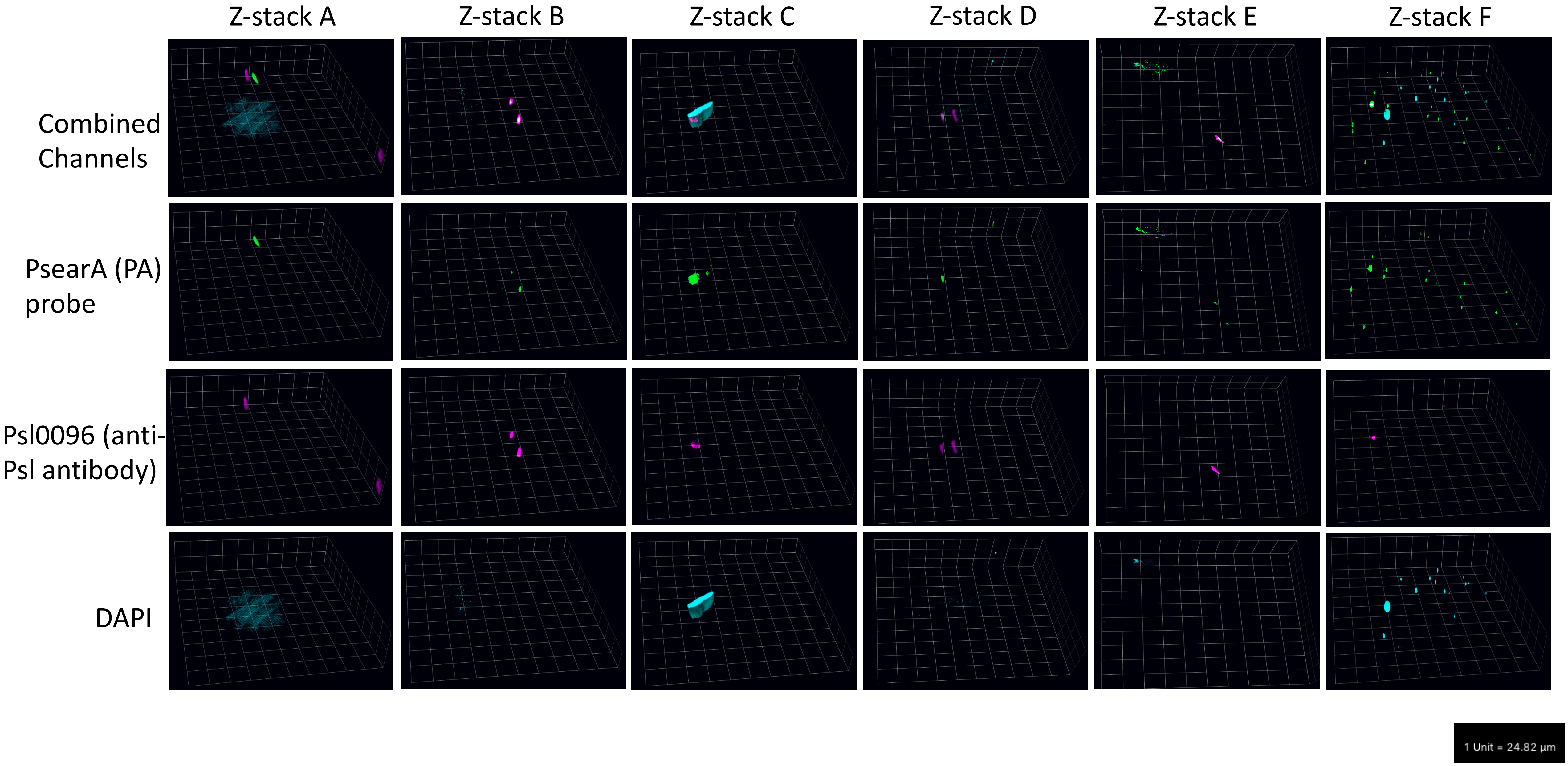

PAE004 Slice #3 (Eradicated)

20X Objective

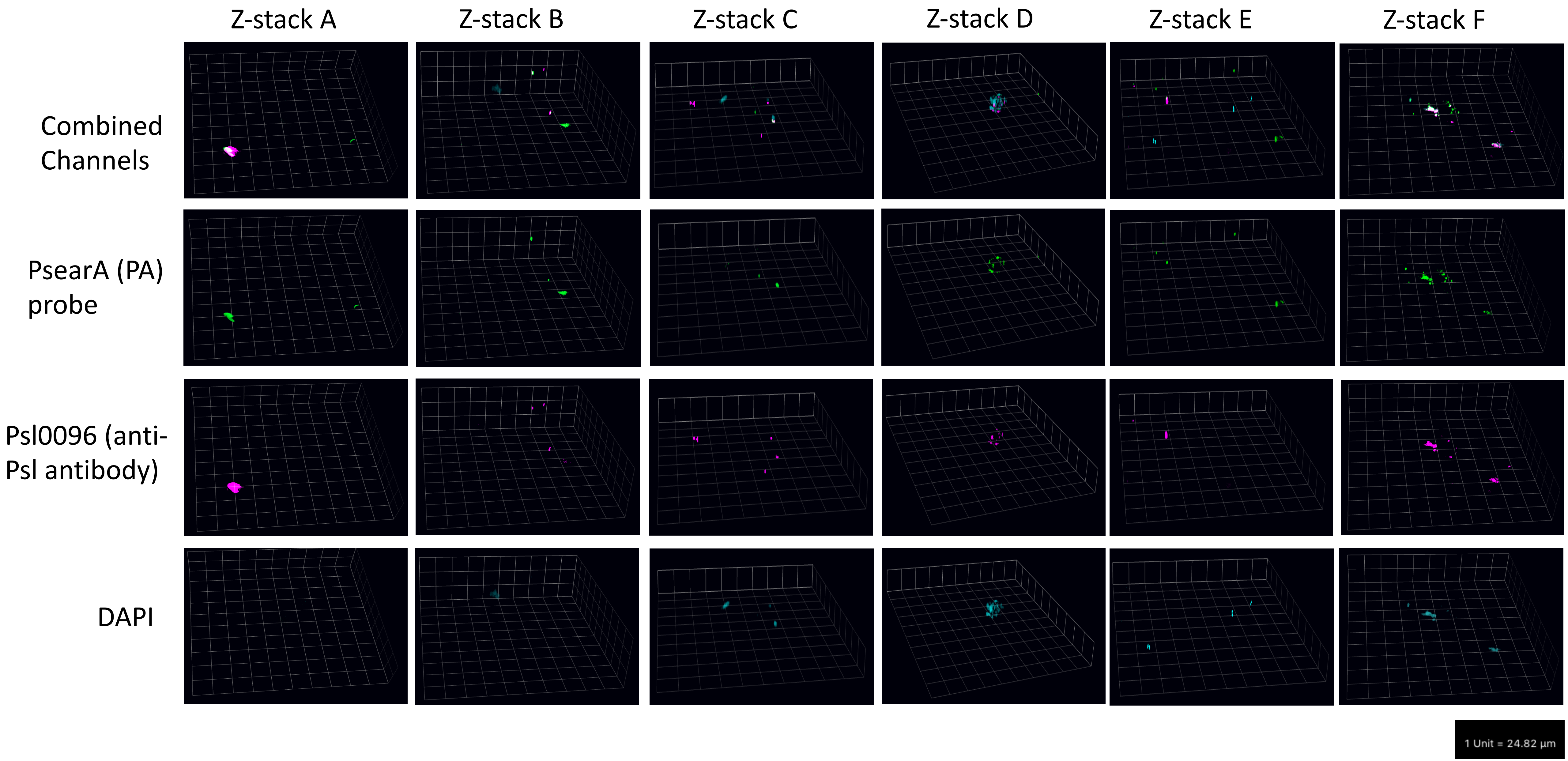

PAE006 Slice #1 (Eradicated) 20X Objective

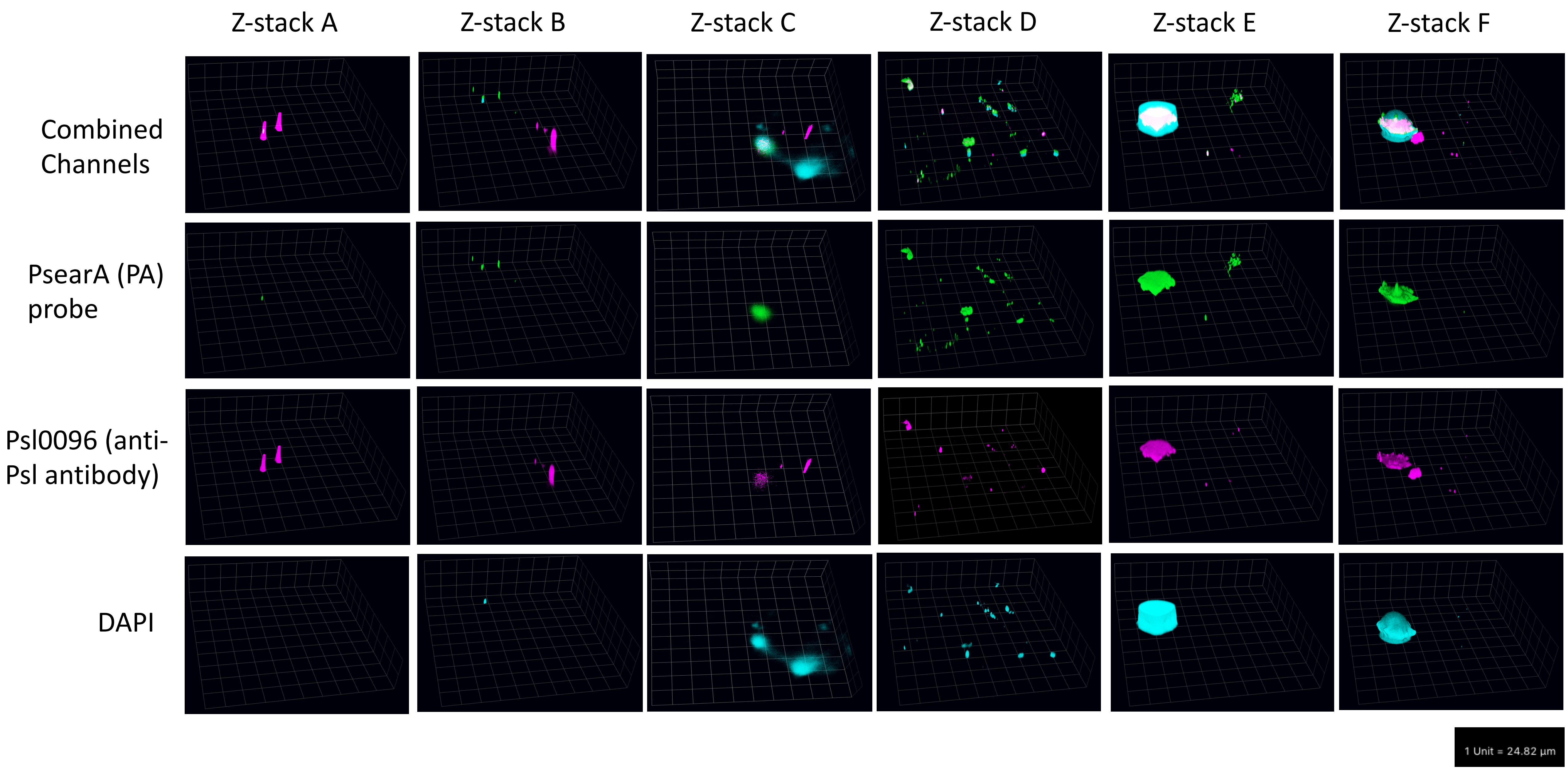

PAE006 Slice #2 (Eradicated) 20X Objective

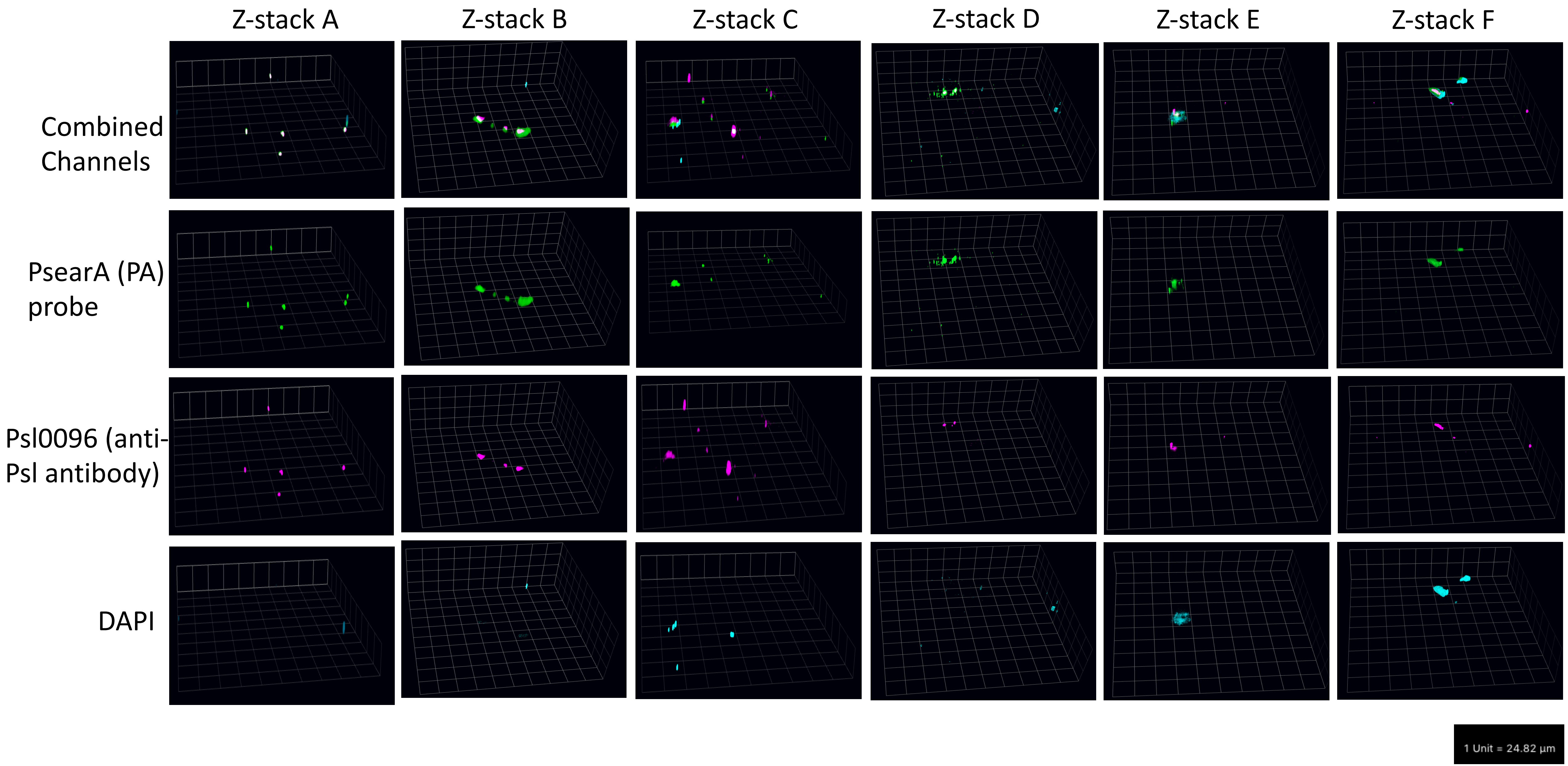

PAE006 Slice #3 (Eradicated)

20X Objective

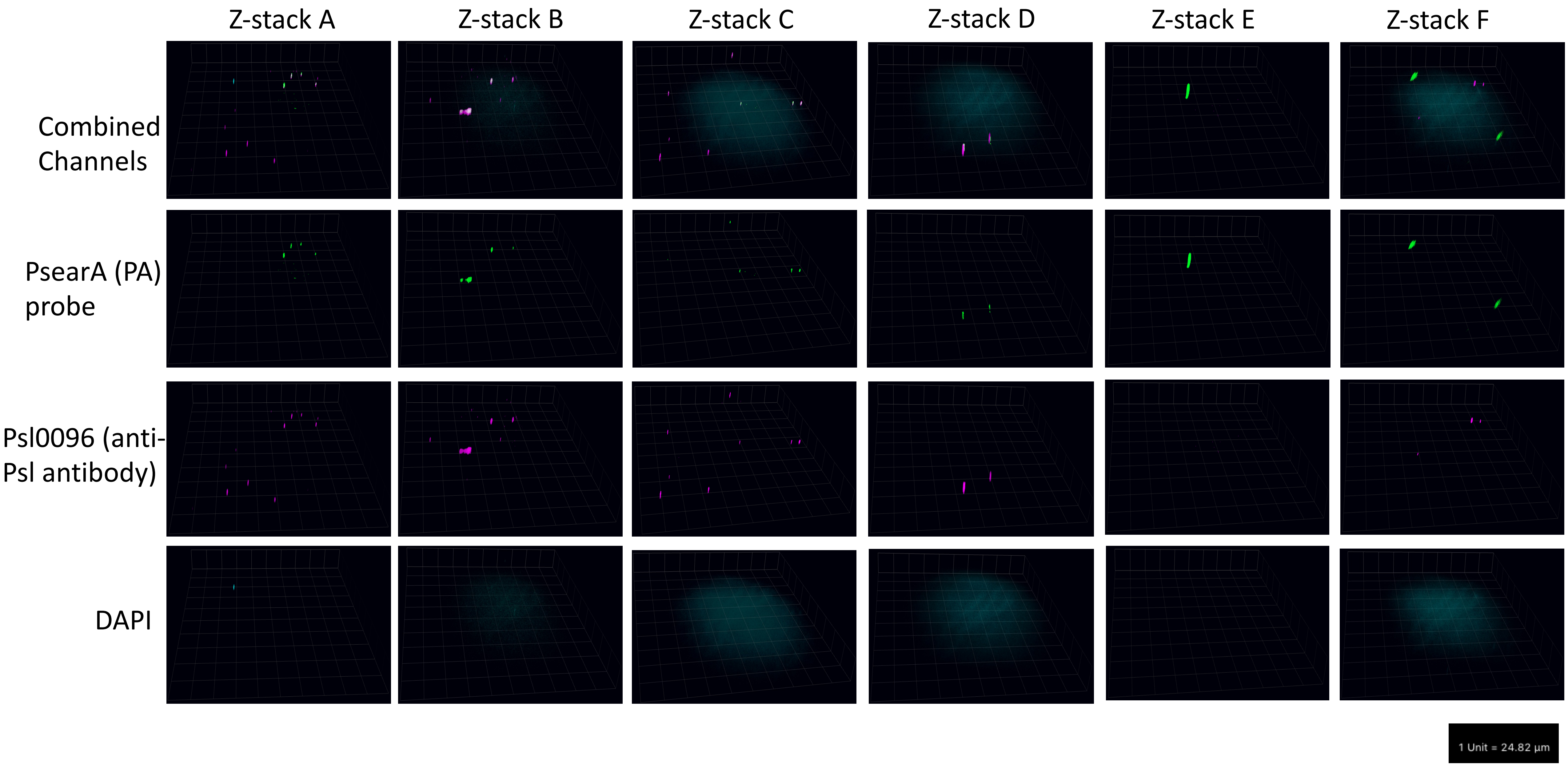

PAE007 Slice #1 (Eradicated)

20X Objective

Z-stack A | Z-stack B | Z-stack C | Z-stack D | Z-stack E | Z-stack F

Combined Channels

PsearA (PA) probe

Psl0096 (anti-Psl antibody)

DAPI

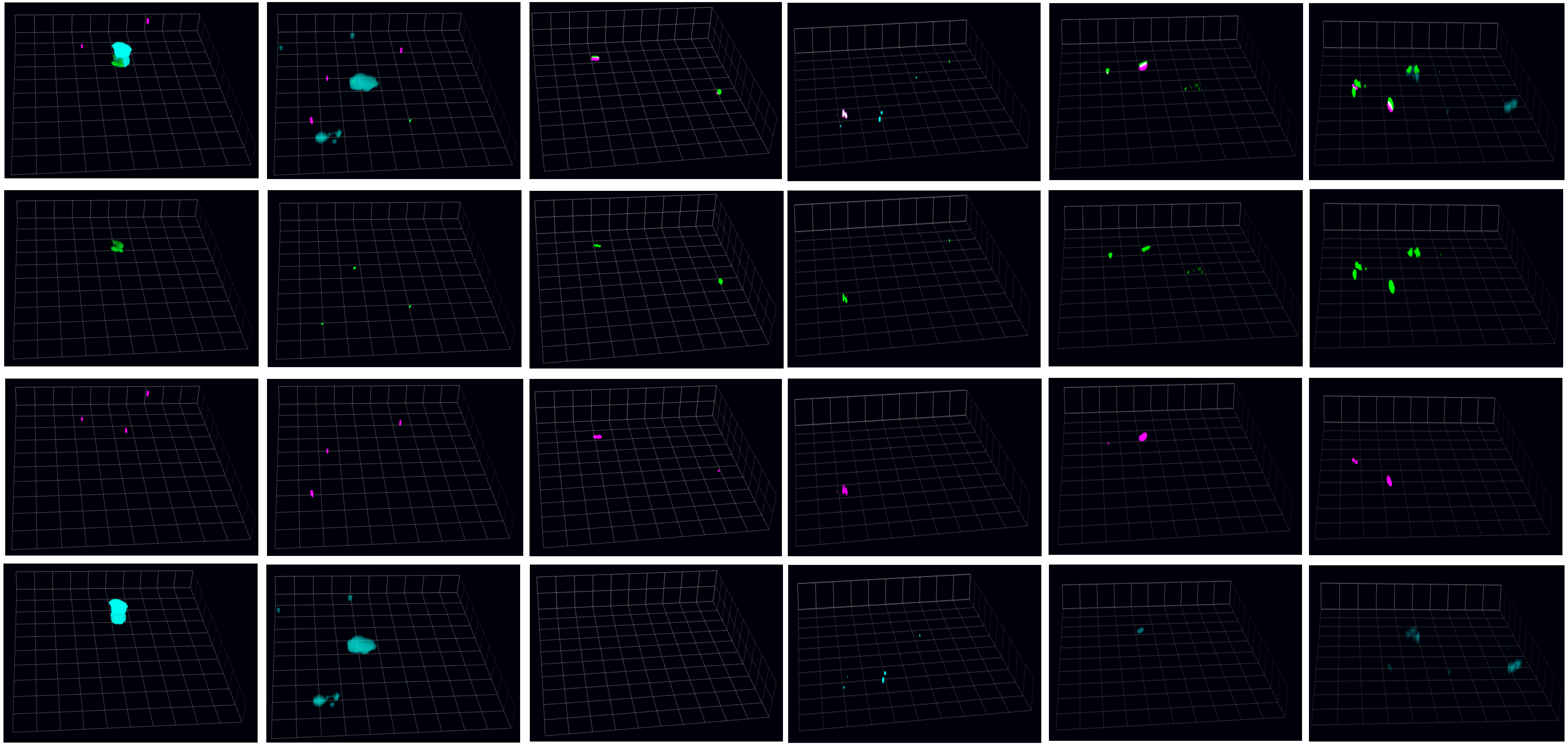

1 Unit = 24.82 µm

PAE007 Slice #2 (Eradicated) 20X Objective

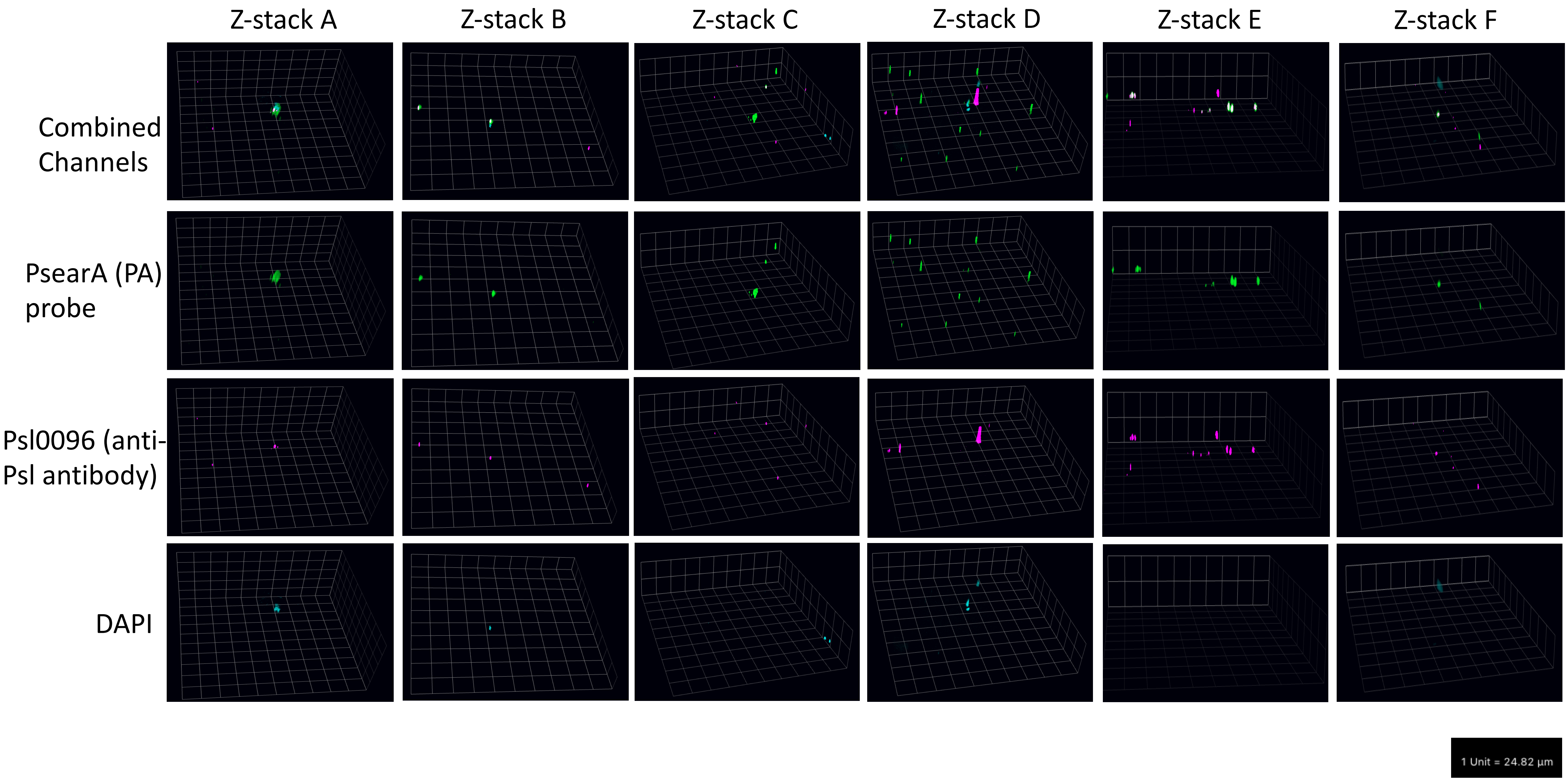

PAE007 Slice #3 (Eradicated)

20X Objective

Z-stack A | Z-stack B | Z-stack C | Z-stack D | Z-stack E | Z-stack F

Combined Channels

PsearA (PA) probe

Psl0096 (anti-Psl antibody)

DAPI

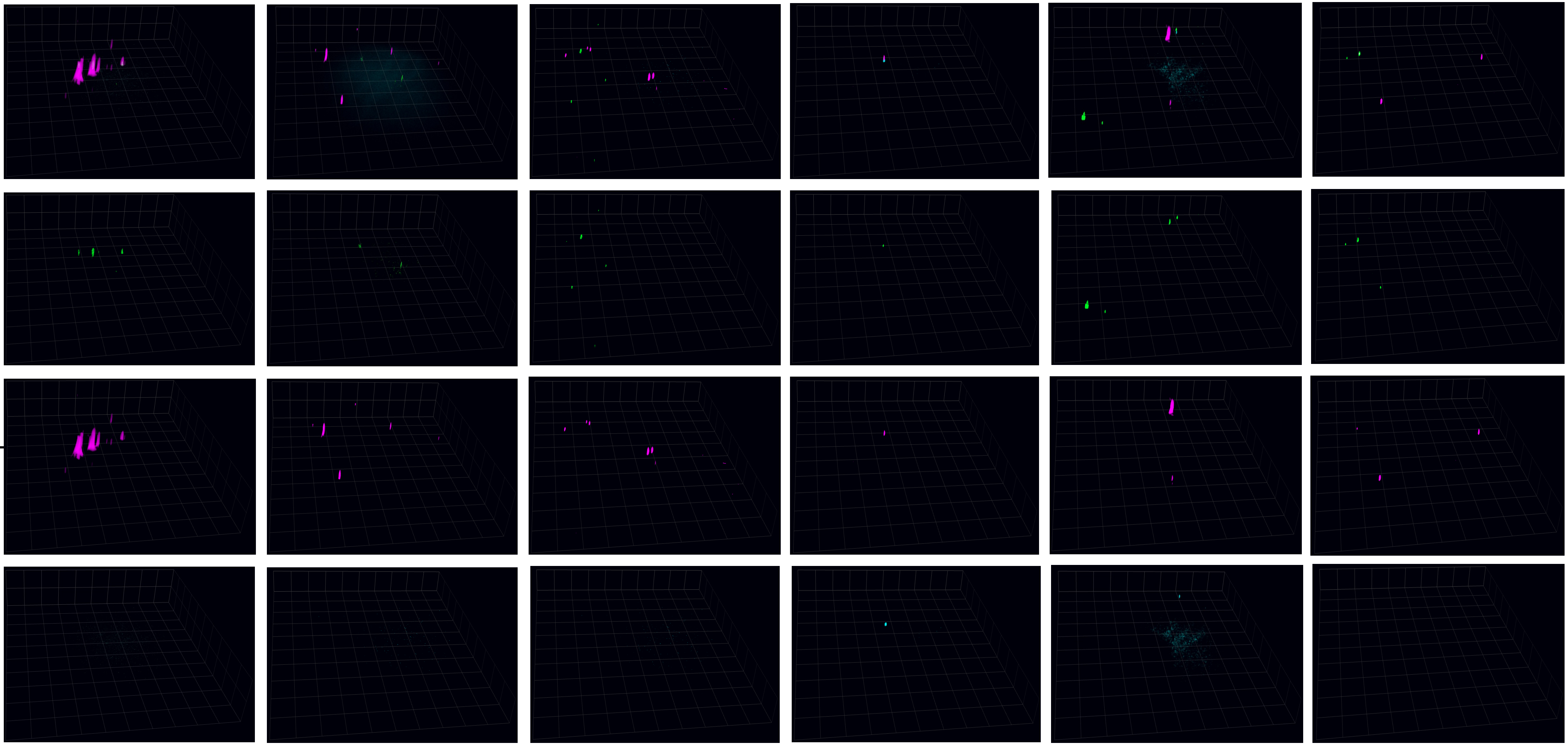

1 Unit = 24.82 µm

PAE010 Slice #1 (Eradicated)

20X Objective

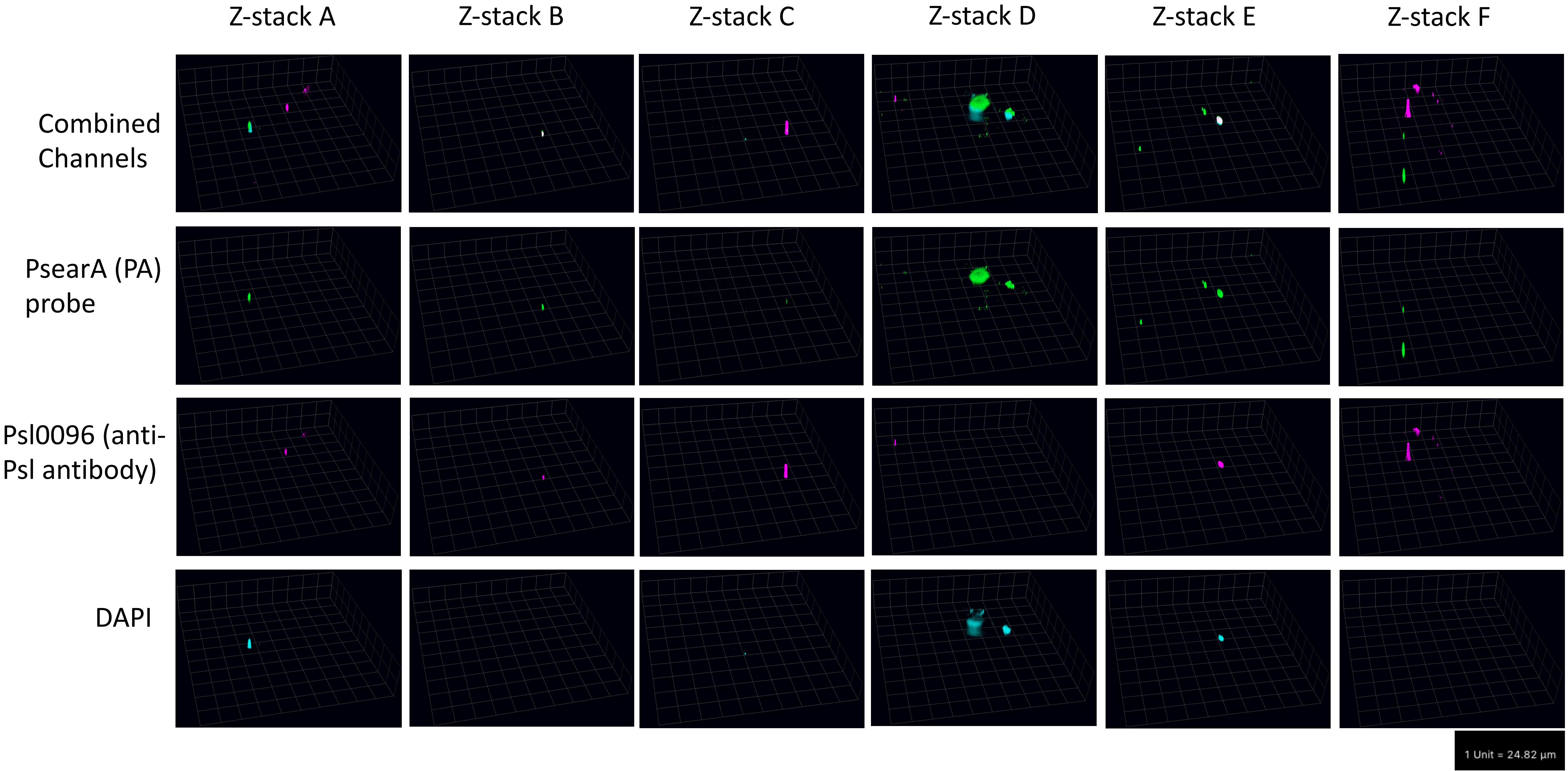

PAE010 Slice #2 (Eradicated) 20X Objective

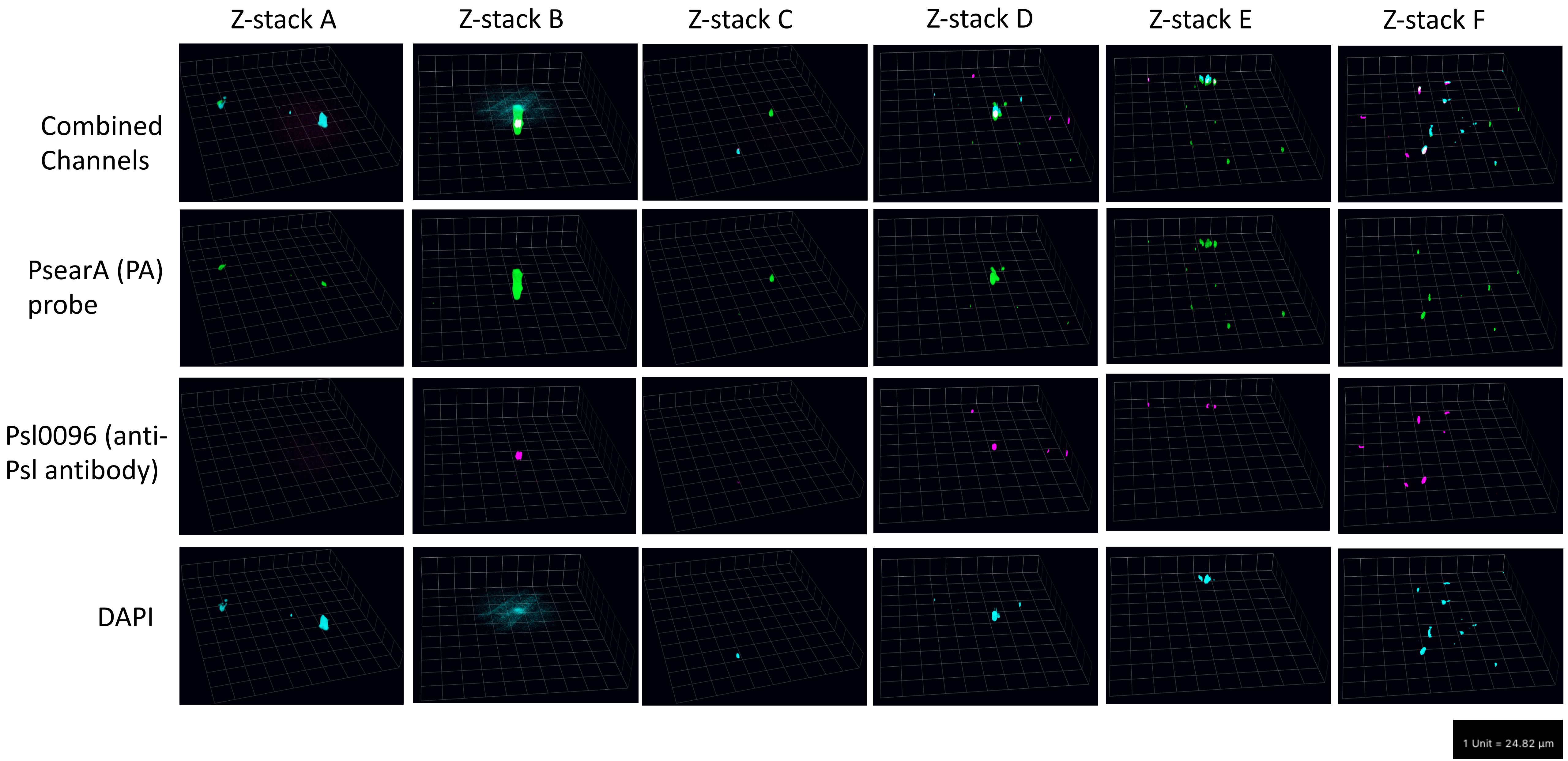

PAE010 Slice #3 (Eradicated)

20X Objective

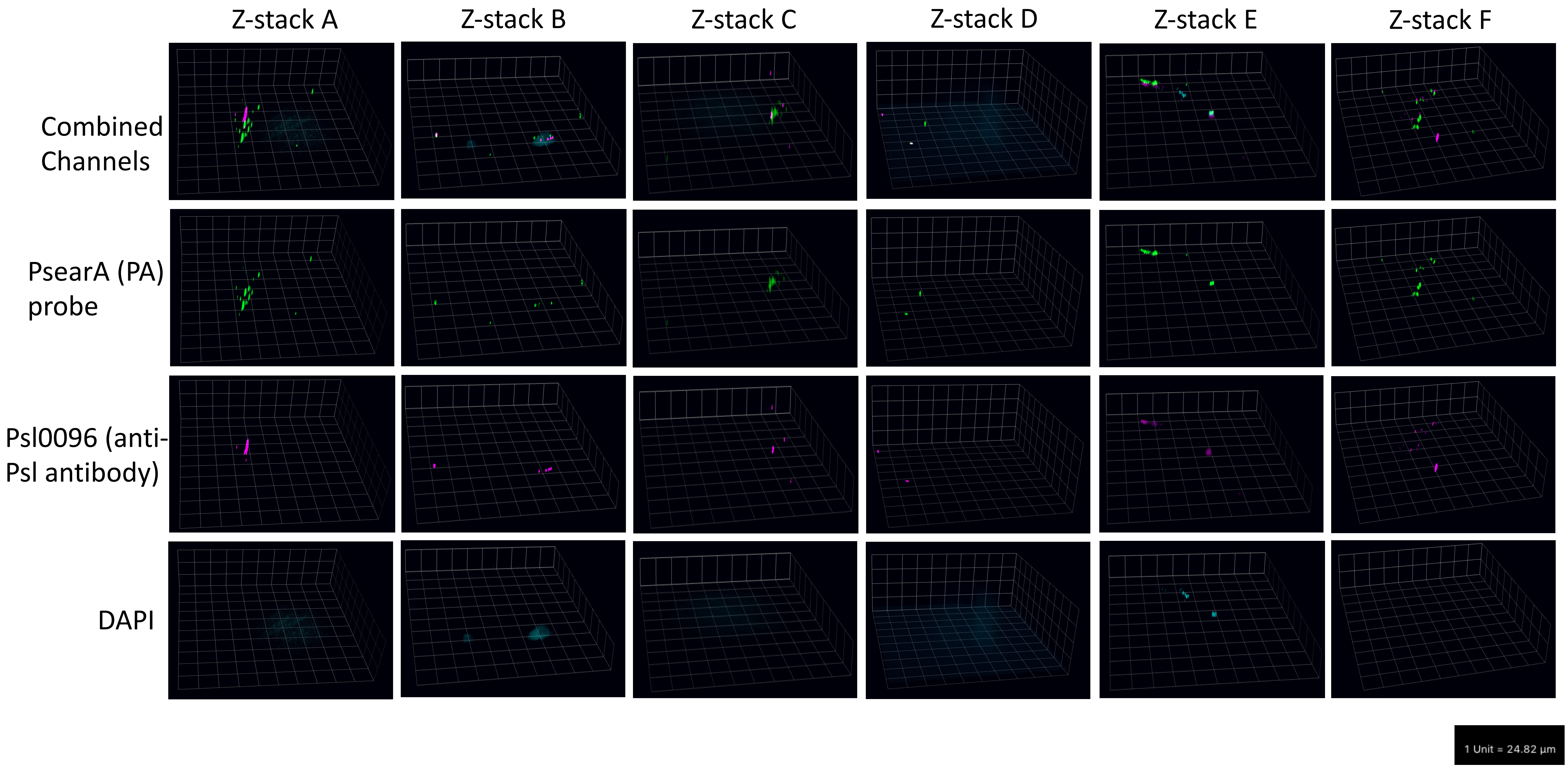

PAE012 Slice #1 (Eradicated) 20X Objective

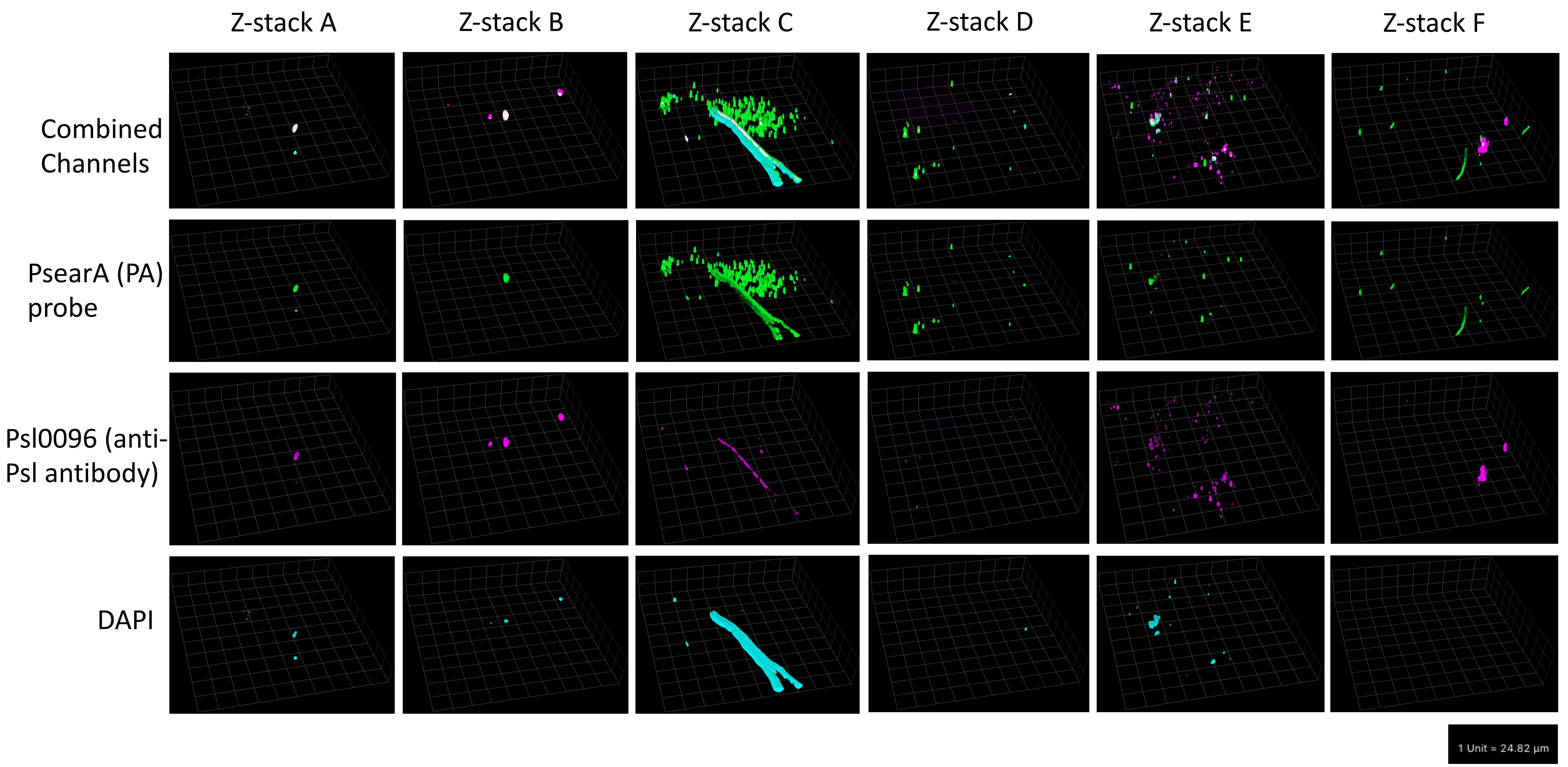

PAE012 Slice #2 (Eradicated)

20X Objective

Z-stack A | Z-stack B | Z-stack C | Z-stack D | Z-stack E | Z-stack F

Combined Channels

PsearA (PA) probe

Psl0096 (anti-Psl antibody)

DAPI

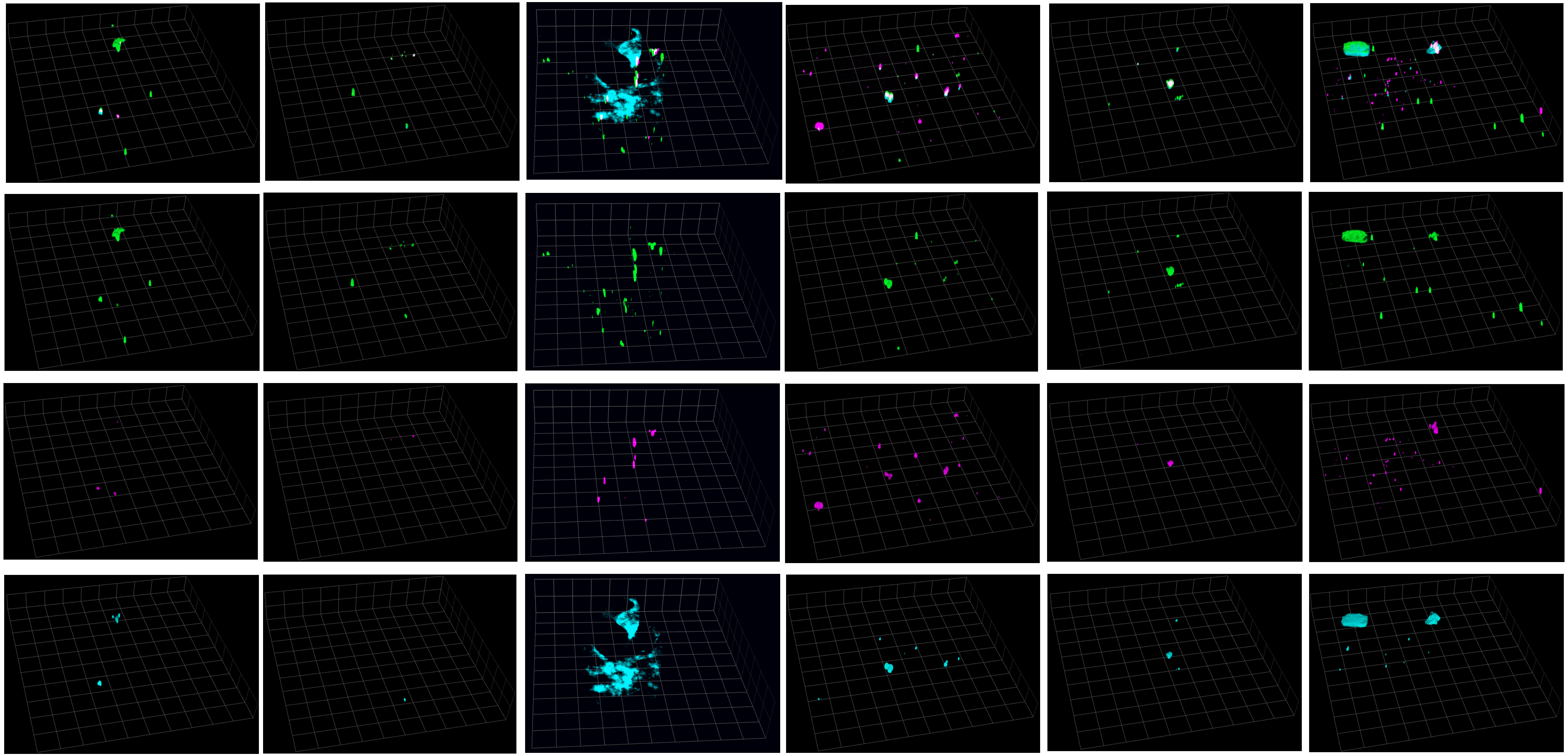

1 Unit = 24.82 µm

PAE012 Slice #3 (Eradicated) 20X Objective

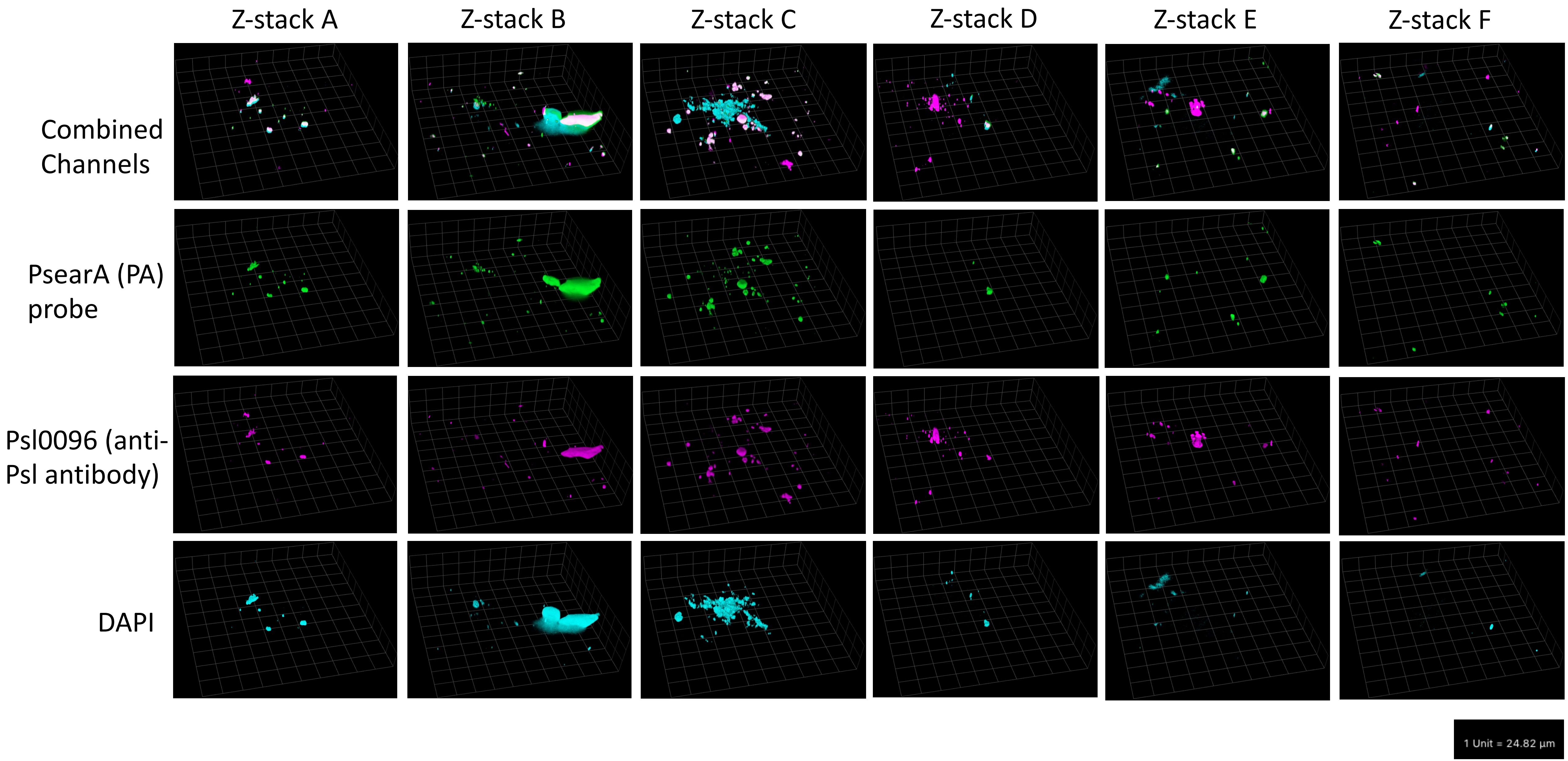

Supplement: Supplementary file 2 — Supplementary Figure 2. [file 41598_2022_25889_MOESM2_ESM.pdf]
